# Impact of Extraction Scale and Method on the Chemical Profile of Essential Oils: A Comparative Study Between Laboratory Hydrodistillation and Semi-Industrial Dry Steam Distillation

**DOI:** 10.3390/molecules31122105

**Published:** 2026-06-15

**Authors:** Norbert Léva, Emese Gál

**Affiliations:** Faculty of Chemistry and Chemical Engineering, Babeș-Bolyai University, Arany János No. 11, 400028 Cluj-Napoca, Romania; norbert.leva@ubbcluj.ro

**Keywords:** essential oils (EOs), steam distillation (SD), hydrodistillation (HD), chemical composition, GC-MS analysis, multivariate data analysis, comparative profile analysis

## Abstract

Essential oils are complex plant-derived volatile blends composed of a myriad of aromatic secondary metabolites. The volatile architecture of plant essential oils suggests a consistent trend under the experimental conditions evaluated, regardless of the distillation scale and methodology. This study presents a comparative chemometric evaluation of two integrated processing systems: laboratory-scale hydrodistillation (HD) of dried biomass versus semi-industrial-scale dry steam distillation (SD) of fresh biomass. Seven economically important botanical species spanning three families were analyzed: *Lavandula angustifolia*, *Salvia officinalis*, *Hyssopus officinalis*, *Mentha piperita*, *Mentha spicata*, *Achillea millefolium*, and *Picea abies*. Gas chromatography–mass spectrometry (GC-MS) profiling revealed that HD consistently yielded a more chemically diverse volatile profile than SD. Unsupervised Principal Component Analysis (PCA) and Hierarchical Cluster Analysis (HCA) achieved absolute binary segregation between the HD and SD fractions for every species. Supervised Partial Least Squares Discriminant Analysis (PLS-DA) established robust predictive models (Q^2^ cum > 0.98), isolating specific chemical markers responsible for the variance. The results prove a universal physical trend: HD significantly enriched low-boiling oxygenated derivatives (such as oxygenated monoterpene alcohols and oxides), while SD selectively preserved heavier, thermally sensitive hydrocarbon fractions across all taxonomic groups. Ultimately, combining GC-MS with multivariate chemometrics provides an objective, automated framework for quality control, authentication, and industrial process optimization in the essential oil sector.

## 1. Introduction

In recent decades, plant extracts and essential oils (EOs) have garnered significant attention in both academic research and the industrial sector, driven by a growing preference for natural substances over synthetic alternatives [1]. Extraction methods that utilize water-based, organic solvent-free processes align closely with the principles of green chemistry. Furthermore, these extracted secondary metabolites often possess diverse therapeutic properties, contributing to human well-being or serving as potential lead compounds for drug development [2]. EOs are complex liquid mixtures of biologically active volatile organic compounds (VOCs). These mixtures primarily comprise monoterpenes and sesquiterpenes, along with their oxygenated derivatives (terpenoids), as well as phenylpropanoids in varying proportions. These phytochemicals are responsible for a wide array of biological activities, including antimicrobial, antiviral, anti-inflammatory, and antioxidant effects [2,3]. Consequently, the global demand for high-quality EOs continues to rise across the aromatherapy, cosmetic, and pharmaceutical industries [4].

A vast majority of scientific literature focuses on laboratory-scale essential oil extraction, predominantly employing HD and SD due to their cost-effectiveness and operational simplicity. This trend is underscored by a recent bibliometric analysis, which revealed that 90% of the articles published in the Journal of Essential Oil Research between 2019 and 2021 reported HD or SD as the primary isolation technique, with HD accounting for 85% of these cases [5]. In stark contrast, SD remains the pre-eminent industrial-scale isolation method, accounting for approximately 93% of global EO production [6].

The fundamental differences between HD and SD reside in the physicochemical interactions between the steam and the plant matrix. In Clevenger-type HD, the prolonged contact of plant material with boiling water may promote hydrolysis or the thermal degradation of thermolabile compounds, such as esters and certain monoterpenes [7]. Conversely, semi-industrial dry SD utilizes pressurized steam, which may lead to different mass-transfer kinetics and diffusion rates of volatile constituents [8,9]. Understanding these variations through GC-MS—the gold standard for EO chemical fingerprinting—is essential for ensuring that industrial-scale production maintains the therapeutic and olfactory profiles established in laboratory pilot studies. Furthermore, bridging the gap between bench-top experiments and pilot-scale production is a key requirement for process optimization and energy efficiency within the framework of modern green extraction technologies.

The selectivity of the extraction process is fundamentally governed by the physicochemical properties of the volatile constituents, including their boiling points, vapor pressures, and water solubility. In HD, the plant material is immersed in boiling water, creating a system where polar and oxygenated compounds (such as alcohols and phenols) may exhibit higher solubility in the aqueous phase, potentially leading to their partial loss or delayed recovery. Furthermore, the constant presence of liquid water at 100 °C facilitates the re-arrangement of sensitive terpenes and the de-esterification of key aromatic molecules. In contrast, during dry SD, the absence of a surrounding liquid phase reduces the risk of hydrolysis and allows for a more efficient recovery of hydrophobic sesquiterpenes and high-boiling-point components through the mechanism of hydro-diffusion. This transition from laboratory HD to semi-industrial SD typically alters the hydrocarbon-to-oxygenated derivative ratio, creating a distinct ‘chemical signature’ for the resulting oil [10].

Although SD is by far the most cost-effective extraction method, large-scale, comprehensive studies characterizing the chemical profiles of EOs obtained via industrial SD are relatively scarce. To date, such comparative research has been limited to a few species, including *Citrus aurantiifolia* (lime) [11], *Citrus grandis* [12], *Pelargonium* sp. ‘Kelkar’ [13], *Mentha piperita* [14], and *Lavandula angustifolia* ‘Mill’ [15]. Furthermore, some reports on large-scale distillation focus only on major chemical constituents, often utilizing specialized equipment, such as conical distillation units with integrated homogenizers [16], which may not be representative of standard industrial practices.

The chemical profile of EOs is typically characterized by a few dominant major constituents, accompanied by numerous minor and trace compounds. Given that HD is the most extensively documented extraction method, the established major components for several key species are well-defined: lavender (*Lavandula angustifolia*) is characterized by β-ocimene, linalool, and linalyl acetate [17]; sage (*Salvia officinalis*) by α-thujone, β-thujone, camphor, and viridiflorol [18]; and hyssop (*Hyssopus officinalis*) by β-pinene, pinocamphone, isopinocamphone, and elemol [19]. Similarly, peppermint (*Mentha piperita*) is rich in menthol and menthone [20]; spearmint (*Mentha spicata*) is dominated by eucalyptol, carvone, and carveol [21] or the chamazulene chemotype of yarrow (*Achillea millefolium*)—the main constituents include sabinene, β-caryophyllene, α-farnesene, and chamazulene [22]; whereas spruce (*Picea* sp.) typically yields α-pinene, β-pinene, camphene, limonene, borneol, and bornyl acetate [23].

The primary objective of the present research was to evaluate and compare the chemical profiles of essential oils obtained via a semi-industrial-scale dry SD unit and a laboratory-scale Clevenger-type apparatus (HD). Consequently, rather than isolating extraction mechanics under perfectly equivalent parameters, this study evaluates the chemical shifts that occur during a realistic industrial transition, comparing integrated processing routes that encompass simultaneous variations in extraction scale, biomass moisture content, and matrix fragmentation.

The study investigated seven oil-bearing species representing three distinct botanical families: *Lamiaceae* (*Lavandula angustifolia*, *Salvia officinalis*, *Hyssopus officinalis*, *Mentha piperita*, *Mentha spicata*), *Asteraceae* (*Achillea millefolium*), and *Pinaceae* (*Picea abies*). To the best of our knowledge, this work represents the first systematic evaluation of the chemical composition of sage, hyssop, mints, yarrow, and spruce EOs produced using semi-industrial-scale distillation equipment. By correlating these findings with GC-MS analysis, this study aims to elucidate how scaling up from laboratory to semi-industrial conditions affects the volatile profile of these economically significant plants.

Given that essential oil volatile fractions comprise a complex matrix of major, minor, and trace components, conventional univariate evaluations often fail to capture systemic chemical shifts. Therefore, this study integrates unsupervised chemometric tools—specifically Principal Component Analysis (PCA) and Hierarchical Cluster Analysis (HCA)—to objectively map global compositional variances and uncover hidden extraction patterns without a priori grouping bias.

## 2. Results and Discussion

### 2.1. Yields of Essential Oils Extracted by Hydrodistillation and Steam Distillation

This study provides a comparative investigation of EOs obtained via two distinct extraction methods: semi-industrial-scale dry SD and laboratory-scale HD. Due to logistical constraints regarding the distance between the collection site and extraction facilities, HD of fresh botanical material could not be performed. Currently, the EO industry favors SD for several industrial-scale advantages [5]. The distillation of dried plants has several advantages: by removing the plants’ water content, the mass:EO ratio increases significantly, the oil glands become brittle and porous, and the yield is standardized depending on dry mass. While raw plant materials remain biochemically active and prone to enzymatic degradation, without proper stabilization, the drying process itself presents challenges; volatile ‘top-note’ compounds may be lost during dehydration. At an industrial magnitude the cost of drying is really challenging; moreover, some delicate top notes and aromatic compounds could be lost during the drying process. Consequently, industrial operations often prioritize rapid processing or mobile units over extensive drying. While pre-distillation drying is manageable at a laboratory scale, it becomes logistically and economically demanding at an industrial magnitude. The duration for all HD extractions was standardized at 90 min, whereas the SD process varied from 2 to 7 h. The comparative study revealed that laboratory-scale HD of dried biomass provided significantly higher EO yields (up to 4.5 times greater in *Mentha piperita*) and shorter extraction times (1.5 h) than semi-industrial SD of fresh material (2–7 h). This discrepancy is largely attributed to the removal of moisture during the drying process, which concentrates the secondary metabolites relative to the total mass. Furthermore, the pre-treatment (grinding) applied in HD increased the surface area and ruptured the secretory structures (e.g., glandular trichomes in *Lamiaceae*), facilitating faster oil release within the 1.5 h distillation window. In contrast, semi-industrial SD was conducted on fresh material to simulate standard industrial practices, where immediate processing is preferred to avoid post-harvest degradation. The extended distillation times in SD (up to 7 h for *Achillea millefolium*) were necessary to ensure the hydro-diffusion of heavier constituents from the intact or minimally processed fresh matrix. The higher yields and shorter extraction times recorded for laboratory HD compared to semi-industrial SD are deeply linked to the state of the biomass. Pre-distillation drying and grinding in the HD setup disrupt secretory structures and minimize mass-transfer resistance, whereas the semi-industrial SD system reflects real-world configurations using intact, fresh material where extraction kinetics are governed by slower hydro-diffusion.

These results underscore the impact of pre-distillation drying on yield standardization and emphasize the trade-off between the high efficiency of laboratory-scale HD and the logistical demands of industrial-scale SD processing of fresh botanical material. Comparative data regarding plant material characteristics, extraction parameters, and essential oil yields obtained via laboratory-scale HD and semi-industrial SD for the seven studied species are summarized in Table 1.

It must be emphasized that the chemical variations observed between the HEO and SEO fractions stem from a combination of overlapping processing variables, including extraction scale, biomass moisture content (dried vs. fresh), the degree of matrix fragmentation (ground vs. intact), and distillation kinetics over time. In real-world industrial settings, perfectly isolating the extraction method from the state of the biomass is often logistically unfeasible due to transport distances, storage capacities, and operational costs. Therefore, these results should be interpreted as a comparative evaluation of two complete, realistic processing pipelines rather than a strict isolation of hydrodistillation versus steam distillation mechanics under identical baseline conditions.

The qualitative analysis of the volatile profiles revealed a higher degree of chemical complexity in the essential oils obtained via hydrodistillation (HEOs) compared to those from steam distillation (SEOs). As summarized in Table 2, the number of identified constituents was generally higher in the HEO samples across most species, with the most pronounced difference observed in *Picea abies* (69 compounds in HEOs vs. 52 in SEOs). This trend was consistent for *Lavandula angustifolia*, *Hyssopus officinalis*, and *Achillea millefolium*, suggesting that the HD process facilitates a more comprehensive recovery of the plant’s secondary metabolites.

Interestingly, *Mentha piperita* was the only species where the SEO profile exhibited a slightly higher number of identified compounds (45) than its HEO counterpart (43). Overall, these results corroborate the hypothesis that the direct interaction between the boiling aqueous phase and the plant matrix in laboratory-scale HD promotes the elution of a broader spectrum of volatile constituents, including trace compounds that may remain unrecovered during semi-industrial SD.

### 2.2. Chemical Composition of Essential Oils Extracted by Hydrodistillation and Steam Distillation

The direct water–plant material contact in HD creates a harsher extraction environment that can promote the thermal degradation and hydrolysis of sensible volatile compounds, which are better preserved under the milder SD conditions. Due to this fact, SD is the preferred extraction procedure to obtain EOs industrially. Additionally, these results indicate the relative area% of the oxygenated monoterpenes (OMTs), sesquiterpenes (STs) and oxygenated sesquiterpenes (OSTs) in the HEOs were greater than in the SEOs in general; moreover, a substantial number of compounds were even absent in the SEOs. However, the opposite is true for the area% of the monoterpenes (MTs) (see Table 3, chemical composition of spruce SEO and HEO). In every EO analysis, roughly only the compounds with an area% greater than 0.05% are included, as well as the identified compounds which accounted for more than 95% of the total chromatographic area, respectively. The HD yields (see Table 1) were extrapolated to 1 kg of dried material. The overall HD yields are slightly higher than the SD yields. For the *Lamiaceae* species and yarrow, the extracted EO quantities demonstrate a strong correlation between fresh and dry plant materials, provided the typical 2.5:1 to 4:1 fresh-to-dry weight ratio is applied. In general, the yields of the HEOs from dried plant materials are around 1.5–4.5 times higher than the yields of the SEOs from fresh plant materials, which is a direct consequence of the inherent water content of the biomass. It must be noted that the compositional variations observed between the HEO and SEO fractions are influenced by the joint effects of the extraction method and the pre-distillation state of the biomass (dried vs. fresh). This setup reflects a realistic industrial scale-up scenario, where the loss of highly volatile top notes (such as monoterpene hydrocarbons) during the open-air drying phase of the HD biomass represents a characteristic baseline shift when transitioning from laboratory pilots to fresh-matrix industrial steam processing. The observable similarity of the fresh and dry spruce yields is only explainable by the fact that the spruce EO content has high sensitivity to desiccation and storage time, and the EO components are evaporating rapidly from the dried botanical material.

A superficial comparison based strictly on the absolute number of identified compounds indicates that HD provided a higher chemical richness (69 identified compounds) compared to SD (52 identified compounds). However, to establish a more robust evaluation of the extraction efficiencies regarding structural diversity and relative abundance uniformity, alpha diversity parameters were strictly assessed.

The volatile profile generated by HD exhibited a substantially higher Shannon Diversity Index (H′ = 3.02) than that generated by SD (H′ = 2.29). This clear divergence indicates that HD expands the complexity of the chemical landscape rather than merely extracting trace variations in identical structural groups. This finding is further corroborated by Pielou’s Evenness Index (J′), which rose markedly from 0.58 in the SD profile to 0.71 in the HD profile. This mathematical assessment reveals that while the SD matrix is heavily dominated and heavily skewed by a few prominent monoterpene hydrocarbons (such as α-pinene, β-pinene, and β-phellandrene), the thermal and aquatic environment of the HD process establishes a significantly more balanced, uniform, and distributed extraction yield. This shifting equilibrium structurally manifests as a profound enrichment of low-boiling oxygenated derivatives (e.g., oxygenated monoterpene alcohols and oxides) in the HD profile over the non-polar, pure hydrocarbon species preferred during SD. These combined findings demonstrate that the chosen extraction method modifies not only the sheer quantity of extractable components but fundamentally shifts the functional group distribution, structural class representation, and abundance uniformity of the resulting essential oil.

#### 2.2.1. *Picea abies*

European spruce is native to central, northern and eastern Europe, and is one of the most abundant forest-forming coniferous species in the Carpathians. As its needle-like leaves are covered with epicuticular wax and the EO is stored in the internal resin canals, during extraction the steam must first break down the waxy layer and penetrate the plant tissue to disengage the EO. Thus, the SD is prolonged to about 4–5 h compared to the HD, after which no substantial amount of EO was collected. In the SD process the main components from the EO were α-pinene, camphene, β-pinene, β-myrcene, β-phellandrene, D-limonene, borneol and bornyl acetate. The composition of the extracted volatile fraction with SD is consistent with data found in the literature [23]. These MT and OMT compounds had a combined chromatographic area% about 82%, which are also present in the HEO but with up to five-fold lower area% (see Table 3). In turn, the HEO contained STs, OMTs and OSTs in larger quantities than the SEO; this phenomenon can be observed all over in the results of this study. This outcome supports the observation that HD promotes the isolation of larger molecular structures—specifically STs, OSTs, and diterpenes—under the conditions analyzed. Interestingly, in spruce the number of identified compounds show the largest difference between the SEO and the HEO, which is given by the difference in identified STs and OSTs. The noteworthy STs and OSTs in the HEO are: longifolene, β-sesquiphellandrene, β-farnesene, α-amorphene, *δ*-cadinene, nerolidol, α-cadinol, and farnesol. The only monocyclic diterpene alcohol identified in both samples is thunbergol, which is present at 0.15% in the SEO and 5.09% in the HEO with a 33-fold difference. These results further indicate that HD facilitates the extraction of higher-molecular-mass STs, OSTs, and diterpenes under the evaluated experimental conditions (Supplementary Materials Figures S1 and S5).

Beyond these differences in chemical composition, notable organoleptic observations were made during the processing phase. At the distillation facility, other coniferous species had been processed, namely *Abies alba* and *Pinus sylvestris*. Based on observations, all three species presented more or less initial unpleasant, alkane-like top notes before the characteristic strong, fine, conifer scent, which progressively vanished over time. Determining the cause of this needs additional investigation.

#### 2.2.2. *Achillea millefolium*

Yarrow is native to Eurasia, a perennial plant. One of the major, most important, biologically active, anti-inflammatory components of the yarrow EO is chamazulene, which is highly influenced genetically. Some native types produce it only in trace amounts; however, some cultivars’ EO contains more than 50%, most of it focused in the inflorescence [24,25]. In this study, the selected cultivar was ‘Proa’, which is a chamazulene-rich chemotype. This blue, oil-like compound forms during the plant heat treatment due to the degradation of the naturally present matricin. Therefore, the distillation of yarrow is the most time-consuming procedure, which requires at least 5–7 h. The HEO contained 20.45% chamazulene and the SEO just 9.45%, which is a two-fold difference, although the duration of the SD was at least 3.5 times longer. There are three plausible explanations to this difference: 1—the operated SD installation did not create the optimal conditions for the matricin to decompose properly; 2—the steam temperature was not sufficiently high to promote the decomposition of matricin; 3—since the heat exchanger’s spiral tube was about 7–8 m long with a slope of about 10°, a significant quantity of the viscous, oily, blue chamazulene stuck on the tube’s inner surface and gravitationally could not flow down fast enough, thus a significant amount of it was absent in the collected oil. Considering these, it is evident that the SD procedure needs to be further optimized.

The identified major components of the SEO are sabinene, β-pinene, 2,4-dimethyl-octa-2,6-diene, germacrene D, β-caryophyllene, caryophyllene oxide and chamazulene; on the other hand, the HEO contains sabinene, β-pinene, 2,4-dimethyl-octa-2,6-diene, β-linalool, terpinen-4-ol, linalyl acetate, β-caryophyllene, germacrene D, zingiberene, caryophyllene oxide and chamazulene as main components. The obtained results are consistent with previously published data [22,24,26,27] (See Supplementary Materials Table S3).

#### 2.2.3. *Lavandula angustifolia*

Lavender is native to the Mediterranean region and possibly the most common EO-bearing plant cultivated for ornamental, therapeutic, aromatherapeutic and, not least, culinary purposes. The comparative analysis of the SEO and HEO resulted in minor quantities of MT hydrocarbons in both samples, except the β-ocimene isomers. The major components of the EOs for both extraction procedures are present in almost similar, comparable quantities (β-linalool, terpinen-4-ol, linalyl acetate, lavandulyl acetate, β-farnesene), or in a two-fold (lavandulol, β-caryophyllene, caryophyllene oxide) or higher difference (furanoid linalool oxide isomers, borneol, n-hexyl butanoate, α-terpineol, neryl acetate, geranyl acetate). Chromatographic analysis revealed over ten additional compounds in the HEO compared to the SEO (See Supplementary Materials Figure S2). Although detected at low relative abundances, their presence further demonstrates that HD facilitates the extraction of higher-molecular-weight substances. Attention should be drawn to the fact that these seemingly slight modifications of the EOs lead to a significant change in their organoleptic properties. The SEO had a full, pleasant, sweet lavender scent, while the HEO had a strong, fresh grassy smell alongside the lavender scent. The grassy smell faded over time in the lavender EO kept at 4 °C; after about 5 months this grassy smell almost disappeared, which suggests the necessity of a further investigation of the change in composition over time.

#### 2.2.4. *Hyssopus officinalis*

Hyssop is indigenous from the Caspian Sea region, through the Middle East to southern Europe. However, hyssop is native to warmer regions than the Curvature Carpathians, and it is hardy to USDA 4 to 9 [28]. The hyssop EO has two most important components: the two isomers of pinocamphone. In the analyzed hyssop SEO and HEO, isopinocamphone (*cis*-pinocamphone) presented a relative abundance of 39.57 ± 0.0495% and 39.58 ± 0.0424% respectively, and *trans*-pinocamphone 8.11 ± 0.1202% and 8.12 ± 0.1131%, consistent with the existing data [29,30]. Although in the case of these species-specific compounds no significative difference can be observed between the two extraction methods, the HEO repeatedly resulted in ten more identified compounds (See Supplementary Materials Figure S3 and Table S5). Considering the hedycaryol-elemol peak area ratios, meaningful conclusions can be drawn. In the SEO both hedycaryol and elemol are present with 2.17% and 0.12%, but in the HEO only the elemol is present with a peak area of 10.4%; an approximately 87-fold discrepancy (0.12% vs. 10.4%) was observed, representing a difference of nearly two orders of magnitude. The presence of both hedycaryol and elemol in the SEO indicates that only a portion of the naturally present hedycaryol is extracted and about 5.5% of the hedycaryol is thermally degraded to elemol [31]. On the other hand, the large amount of elemol signifies the total degradation of hedycaryol during the HD. Other identified substances with similar peak areas in both EOs are α-pinene, β-pinene, β-myrcene, β-phellandrene, perillyl methyl ether, β-caryophyllene, aromadendrene, germacrene D and γ-elemene, in agreement with reported data [19,32].

#### 2.2.5. *Salvia officinalis*

Sage, like hyssop, is native to the Mediterranean region and hardy to the Curvature Carpathians’ harsh winters, and therefore cultivable for EO production. The main concern about the application of sage EO comes from its occasionally high α-thujone and β-thujone content with neurotoxic activity [33]. The combined amount of these constituents can exceed 50%, hence these EOs with high ketone content must be used and applied cautiously. In our case, the analyzed sage SEO and HEO α-thujone and β-thujone amounts are relatively low: 12.95% and 4.73%, and 13.59% and 5.63%, respectively. These comparable relative abundances are persistent in the case of other OMTs like α-terpinolen, camphor, borneol and bornyl acetate: 0.21% and 0.245%; 12.93% and 14.61%; 2.99% and 2.94%; and 2.8% and 1.9% respectively. Repeatedly, it can be observed that the peak area% of MTs and STs are generally larger in the SEO than in the HEO, but the opposite is true for the peak area% of OMTs and OSTs, with notable exceptions in the case of eucalyptol (11.53% and 6.65%) and β-linalool (7.55% and 1.25%). The differences in abundance among the extracted STs were less pronounced than those observed for the OSTs, suggesting that the recovered STs more accurately reflect their total concentration in the plant material. For instance: β-caryophyllene 13.79% (SEO) and 7.38% (HEO) (two-fold discrepancy), viridiflorol 2.88% and 11.13% (4-fold discrepancy). Notably, the levels of epimanool, a labdane terpenoid alcohol, exhibited a substantial 10-fold variance, 0.82% in the SEO and 8.66% in the HEO, another proof of the efficiency of HD to extract large molecular mass substances (See Supplementary Materials Figures S3 and S6, and Table S1).

#### 2.2.6. *Mentha piperita*

Peppermint is a cross-hybrid between *Mentha aquatica* and *Mentha spicata*, native to Europe and the Middle East. Whereas peppermint has many cultivars, the variety of the composition of the distinct EOs is obvious; the main components are menthol, menthone, menthofuran and eucalyptol. The peppermint EO’s main quality indicator is the menthol:menthone ratio. According to ISO 856:2006 [34], the EO’s maximum menthol and menthone content is 49% and 28%; the max. menthofuran and pulegone content are both 8%, but desirably should be as low as possible in each case to be considered safe for human use (ISO 856:2006). Unfortunately, none of the EOs obtained by SD and HD meet the minimum standard criteria. Both unwanted OMT ketone—menthone and pulegone—quantities are higher in each EO than the recommended maximum in the standard; in the SEO and HEO the menthone and pulegone content were found to be 59.83% and 41.97%, and 5.49% and 11.02% respectively. Moreover, the obtained Eos’ menthol content reached just 17.11% and 21.38%, which is about 11% less than the minimum required for meeting the standard. Considering these findings, this high-menthone chemotype peppermint cultivar’s replacement is mandatory for the production of a safer EO (See Supplementary Materials Table S2).

#### 2.2.7. *Mentha spicata*

Spearmint is indigenous to Europe and Asia, and its EO is extensively utilized as a food additive and in aromatherapy. The most important component of this species’ EO is carvone, a terpenoid ketone, which also determines the quality of the EO, ideally up to 70–80 area%. The SDE and the HEO contained 79.12% and 74.96% carvone, respectively, both above the desired 70%. Other substances with an area% greater than 1% are β-myrcene, D-limonene, eucalyptol, *cis*-β-terpineol, terpinen-4-ol and β-caryophyllene [21,35] (See Supplementary Materials Table S4).

## 3. Materials and Methods

### 3.1. Plant Material

All the plants from the *Lamiaceae* and *Asteraceae* families were cultivated in Romania, Transylvania, Covasna County, in the peripheral area of Târgu Secuiesc, at 46°0′30″ N latitude and 26°8′40″ E longitude, an area corresponding to USDA Plant Hardiness Zone 6a [36]. The plantation was two years old in 2025 and the harvest for this study was performed in the same year. The harvest was realized at full bloom during the 10 a.m. to 2 p.m. time window in every case and the plants were transported to the SD facility immediately where the exactions were carried out as soon as possible. The shade-dried plant materials for HD were kept in proper condition until further use.

As for the spruce, the plant material originated from a nearby forest about 13–15 km away as forestry waste. The fresh branches were grinded before the SD while the dried branches were processed in the same manner as the *Lamiaceaes*.

The plant material for all seven species was harvested between June and August 2025. To ensure chemical standardization, specific plant organs were selectively gathered at their optimal phenological stages: fully expanded leaves for the mint species (*Mentha piperita* and *Mentha spicata*); fresh flowering tops for *Lavandula angustifolia*, *Salvia officinalis*, *Hyssopus officinalis*, and *Achillea millefolium*; and terminal twigs bearing fresh needles for *Picea abies*.

### 3.2. Data Processing

All multivariate chemometric computations, including unsupervised Principal Component Analysis (PCA), Hierarchical Cluster Analysis (HCA), and supervised Partial Least Squares Discriminant Analysis (PLS-DA), were performed using XLSTAT (version 2023.1, Lumivero, Denver, CO, USA) [37]. Prior to multivariate modeling, the raw GC-MS peak areas were normalized to relative percentages. The resulting dataset was then subjected to auto-scaling (unit variance scaling) within the chemometric platform to standardize the chemical variables and eliminate magnitude scale effects between high-abundance major constituents and lower-abundance trace compounds. The corresponding plots and projections were exported directly from the software.

### 3.3. Essential Oil Production and Extraction

#### 3.3.1. Steam Distillation—SD

The process of the SD was completed with a hand-operated non-automatic semi-industrial-scale dry steam distillation equipment made of food-grade stainless steel. The apparatus has four main components: 1—a 150 L steam generator fueled by wood; 2—a 600 L tank for the plant materials; 3—a 300 L condenser with approximately seven m long spiral condensation tube; and 4—Florentin-type EO separator for the organic phase and the floral water. Depending on the species, the fully filled 600 L tank of the plant material weighed around 140–170 kg. Since the equipment is fully manual, the steam flow rate can only be influenced by the burning wood quantity, which was about 9–10 L/h in every case.

Although this scale of equipment operates without integrated digital sensors for direct pressure and temperature logging, strict manual standardization protocols were implemented to guarantee batch-to-batch reproducibility. The thermal input was regulated by standardizing the mass of wood fuel supplied to the furnace per hour, and the boiler water level was kept static throughout the runs. Process stability was actively monitored and verified via the distillate output, with the operator adjusting the furnace draft to maintain a highly consistent condensation flow rate of 9.5 ± 0.5 L·h^−1^. This steady-state volumetric output served as a reliable physical proxy for a uniform steam flow rate and thermal equilibrium across all evaluated extractions.

The durations of distillation and the yields are highly influenced by the plant species (see Table 1), in each case the distillation runs were officially terminated based on an objective operational endpoint, defined as the moment the volume of the isolated organic phase (essential oil) in the Florentine flask did not increase by more than 0.1 mL over a continuous 30 min observation window.

#### 3.3.2. Hydrodistillation—HD

The HD of the seven plant species was performed in laboratory conditions. The HD of the fresh plant materials was not feasible as well as not recommended. In all instances, a Clevenger-type apparatus [38] was used to extract the essential oils. In total, 70 ± 1 g of dried and finely grinded plant samples were submerged in 1 L of distilled water and the distillations took 90 min, when the amount of obtained oil was constant. The collected essential oils were dried on anhydrous MgSO_4_ and filtered. The obtained EOs were kept at 4 °C until further analysis.

### 3.4. GS-MS Analysis and Identification

GC-MS analysis of the obtained essential oils was carried out using a Gas Chromatograph GC 2010, mass spectrometry MS-QP2010 Plus, and AOC-20i+s autosampler (Shimadzu, Kyoto, Japan). The GC was equipped with a capillary column ZB-5ms Plus (30 m × 0.25 mm, 2.5 µm film thickness; Phenomenex, Torrance, CA, USA). Helium 6.0 (Linde, Timisoara, Romania) was used as the carrier gas, at a constant flow rate of 32.8 cm/s. The GC oven temperature was programed as follows: initial temperature of 40 °C for 1 min, then heated up to 280 °C at 5 °C/min and held at this temperature for 5 min. The temperatures of injector and detector (transfer line and ionization source) were set at 220 °C. A total of 1 µL of diluted essential oil (200 times in CH_2_Cl_2_) was injected in split ratio 1:50. Mass spectrometry was operated in the electron ionization (EI) mode at 70 eV, with mass spectra acquired across a scan range of m/z 35–600.

Volatile constituents were identified by comparing their mass spectra with those stored in the NIST 14 and NIST 17 mass spectral libraries [39,40]. To confirm the identification, experimental linear retention indices (LRIs) were determined [41] utilizing an n-alkane standard solution (C8–C33, 100–200 μg·mL^−1^ in n-hexane, Restek, Bellefonte, PA, USA). The calculated LRI values were subsequently validated through comparison with previously published data from the literature [42].

## 4. Discrimination of Volatile Profiles via Chemometrics (PCA, HCA, and PLS-DA)

To move beyond simple individual compound identification and uncover hidden patterns within complex chemical datasets, unsupervised chemometric analyses—specifically Principal Component Analysis (PCA) and Hierarchical Cluster Analysis (HCA)—were employed. Crucially, PCA was applied uniformly to every plant extract generated in this study, serving as a comprehensive screening tool to map the entire dataset. These multivariate techniques are uniquely suited for exploratory data analysis because they operate without prior knowledge of sample categories or groupings. By evaluating the natural variance and mathematical distances within volatile profiles, these methods enable an objective, unbiased visualization of how different plant extractions cluster based solely on their inherent chemical fingerprints.

To evaluate the robust predictive power of the supervised PLS-DA model and rule out the risk of mathematical overfitting inherent to small sample sizes, a validation routine comprising a 200-iteration random permutation test and a Cross-Validation ANOVA (CV-ANOVA) was applied. The significance threshold for the CV-ANOVA model diagnostics was established at *p* < 0.05.

**Figure 1:**
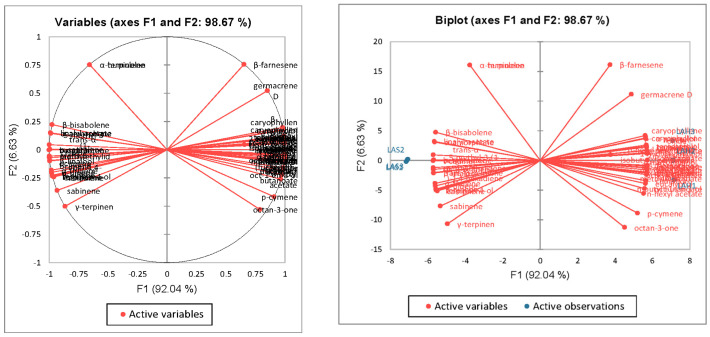
Principal Component Analysis (PCA) of lavender essential oil volatile profiles obtained via steam distillation (LAS) and hydrodistillation (LAH): (**left**) a variable correlation circle, and (**right**) a PCA biplot showing the distribution of active observations (samples) and active variables (volatile compounds) along axes F1 and F2.

To evaluate the impact of extraction methodologies—specifically steam distillation (LAS) and hydrodistillation (LAH)—on the volatile chemical profiles of the lavender essential oils, the GC-MS datasets were subjected to PCA. As the initial step in the chemometric workflow, PCA was deployed to reduce data dimensionality and visualize sample relationships. The structural quality of the PCA model was confirmed via a scree plot. Principal Component 1 (F1) accounted for the vast majority of the total dataset variance at 92.04%, while Principal Component 2 (F2) explained an additional 6.63%. Together, these first two dimensions captured an exceptionally high cumulative variability of 98.67%, ensuring that the subsequent 2D projections retain nearly all relevant chemical information without significant data loss (Figure 1).

**Figure 2:**
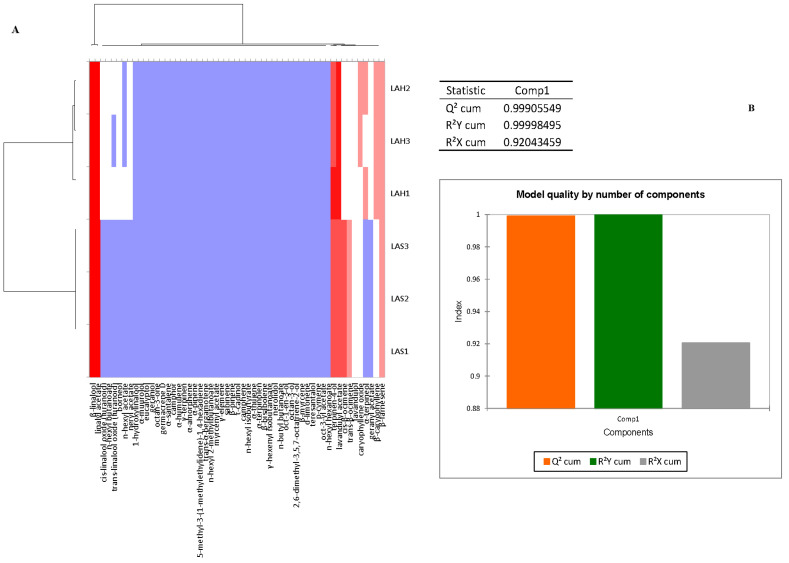
Chemometric evaluation of lavender essential oil datasets across extraction methodologies: (**A**) dual-dendrogram HCA heat map illustrating sample clustering (LAS vs. LAH) and chemical variable distribution, (**B**) cross-validation diagnostics (Q^2^ and R^2^ cumulative values) evaluating PLS model quality.

A definitive, absolute separation between the two extraction methods was observed along the horizontal F1 axis of the PCA biplot (Figure 1). The steam-distilled lavender oil replicates (LAS1, LAS2, and LAS3) clustered tightly on the far-left negative hemisphere (around F1 = −7). Conversely, the hydrodistilled samples (LAH1, LAH2, and LAH3) clustered as a uniform group on the far-right positive hemisphere (around F1 = +7). This stark spatial segregation highlights that water–matrix interactions and thermal dynamics during distillation fundamentally alter the volatile composition of the recovered essential oils. To validate these groupings independently without dimensional constraints, HCA was performed. The resulting dendrogram grouped the samples into two primary, highly isolated clusters separated at a large dissimilarity threshold of approximately 180 (Figure 2A). Cluster 1 exclusively contained the LAS replicates, while Cluster 2 comprised the LAH replicates. The near-zero dissimilarity within each individual branch underscores the high extraction reproducibility of both techniques, while the substantial gap between clusters confirms that the processing styles yield distinct volatile expressions. This global divergence is visually summarized in the two-way agglomerative heatmap (Figure 2A). The row-wise dendrogram perfectly segregates the samples by extraction type, mapping out intense shifts in relative chemical abundance (red/blue transitions) across major volatile compound blocks, such as the β-linalool and linalyl acetate matrices.

To identify the definitive volatile compounds responsible for driving the discrimination between the two processing styles, a supervised Partial Least Squares (PLS) model was established. The PLS model quality index demonstrated that an optimal mathematical fit was achieved using only a single component (Comp1). As shown in Figure 2B the model exhibited a near-flawless explanatory capacity for the treatment categories (R^2^Y cum = 0.9999) and a remarkable cross-validated predictive power (Q^2^ cum = 0.9990), successfully accounting for 92.04% of the underlying predictor variance (R^2^X cum= 0.9204).

**Figure 3:**
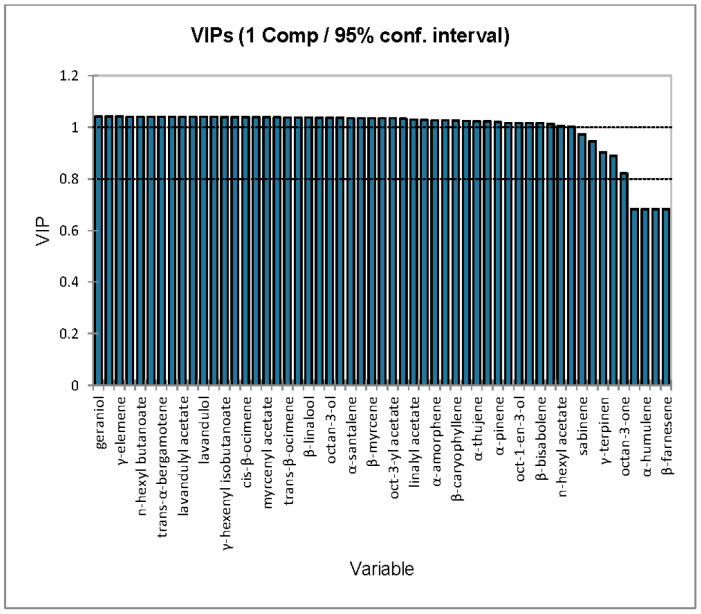
VIP score profile (1 component, 95% confidence interval) of volatile variables from lavender essential oil extractions.

The volatile variables were subsequently ranked according to their Variable Importance in Projection (VIP) scores to pinpoint true extraction biomarkers. Compounds yielding a VIP score greater than the critical threshold of 1.0 are considered statistically significant contributors to model discrimination. Geraniol, γ-elemene, n-hexyl butanoate, trans-α-bergamotene, lavandulyl acetate, and lavandulol emerged as the dominant chemometric markers, topping the VIP index with values exceeding 1.05 (Figure 3).

**Figure 4:**
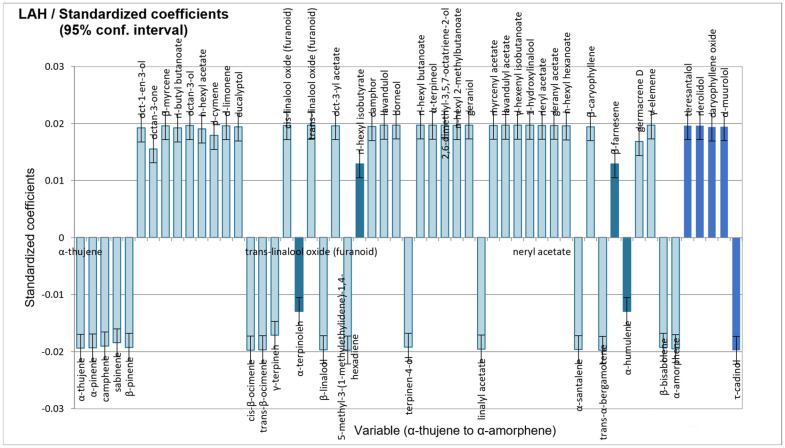
The standardized coefficient profile (95% confidence intervals) for the lavender volatile variables in relation to the hydrodistillation (LAH) group extraction profile.

The specific directional impact of these chemical alterations was elucidated via the PLS standardized coefficients plot (Figure 4). Volatiles exhibiting strong positive coefficients were significantly enriched by or uniquely recovered via hydrodistillation (LAH). This group was characterized by aliphatic structures and specific oxygenated monoterpenes, including oct-1-en-3-ol, octan-3-one, β-myrcene, and D-limonene. Univariate z-testing confirmed the high significance of these findings, with octan-3-one yielding a *p*-value well below 0.01. In contrast, molecules yielding strong negative standardized coefficients were preferentially retained by steam distillation (LAS). This profile was heavily defined by monoterpene hydrocarbons and major bicyclic compounds, including α-thujene, α-pinene, camphene, sabinene, β-pinene, and the core constituent β-linalool.

### Chemometric Meta-Analysis Across Varied Plant Taxa

To confirm whether the stark compositional divergence observed in lavender between steam distillation (LAS) and hydrodistillation (LAH) represents a universal processing trend, the identical chemometric workflow (PCA, HCA, and PLS-DA) was systematically applied to the rest of the plant matrices: *Mentha piperita*, *Achillea millefolium*, *Mentha spicata*, *Hyssopus officinalis*, *Picea abies*, and *Salvia officinalis*. Across all six botanical species, unsupervised HCA and PCA modeling achieved immediate, absolute binary segregation between the LAS and LAH processing fractions. The structural robustness of these independent models was exceptional, uniformly yielding cumulative dataset variance captures (R^2^X cum) exceeding 90%, near-flawless category fitting capacities (R^2^Y cum > 0.99), and high cross-validated predictability thresholds (Q^2^ cum > 0.98) (Table 4).

Due to the discrete sample size, the exceptionally high predictive capacity (Q^2^_cum_ > 0.98) was rigorously cross-validated to confirm model soundness. A 200-iteration random permutation test was performed, yielding a distinctively low R^2^ intercept (<0.30) and a negative Q^2^ intercept (<−0.05), which mathematically verifies that the original model parameters are driven by legitimate chemical variances rather than random data correlation artifacts. Furthermore, the model’s structural stability was confirmed by CV-ANOVA diagnostics, which revealed a highly significant distribution (*p* < 0.001), establishing the definitive validity of the chemometric classification.

Biplot and standardized coefficient interpretations revealed that while the volatile matrices are highly species-specific, a clear structural pattern governs distillation mechanics. Hydrodistillation consistently enriches low-boiling, highly volatile oxygenated structures and specific aliphatic compounds. Conversely, steam distillation selectively favored the preservation of thermally sensitive sesquiterpene fractions and specific monoterpene hydrocarbons across all species, confirming that physical distillation dynamics dictate volatile recovery independent of the underlying plant taxonomy. For a detailed visualization of the individual chemometric distributions, all statistical graphs—including multi-panel PCA, HCA, and PLS-DA projections for each of the six plant matrices—have been compiled in the Supplementary Information (See Supplementary Materials Multivariate Statistical Analysis for every extracted essential oil).

In conclusion, the application of complementary unsupervised (PCA, HCA) and supervised (PLS-DA) chemometric workflows successfully decoded the complex volatile profiles of the studied plant extractions. Across all evaluated botanical matrices, the processing methodology emerged as the dominant factor driving chemical divergence, consistently yielding distinct, highly reproducible volatile expressions. By establishing robust mathematical fits (Q^2^ > 0.98) and identifying definitive VIP extraction biomarkers, these findings demonstrate that chemometric modeling is an indispensable tool for objective quality control, chemical tracking, and the optimization of essential oil distillation processes.

## 5. Conclusions

To the authors’ knowledge, no previous studies have detailed the volatile profiles of these specific aromatic and coniferous matrices harvested within the unique microclimatic conditions of the Târgu Secuiesc Depression, situated at the base of the Curvature Carpathians.

This study provides a comprehensive, comparative evaluation of how extraction scale and methodology—specifically laboratory-scale HD and semi-industrial-scale dry SD—fundamentally shape the volatile chemical profiles of essential oils across multiple plant taxa. By utilizing a highly systematic approach, this work demonstrates that while the baseline volatile matrix is highly species-specific, the underlying physical mechanisms of distillation exert a predictable, systemic effect on volatile recovery.

Unsupervised chemometric modeling (PCA and HCA) achieved flawless binary separation between processing fractions across all seven investigated plant matrices, proving exceptional extraction reproducibility. Furthermore, supervised PLS-DA modeling successfully mapped and isolated key volatile biomarkers, confirming that hydrodistillation preferentially enriches low-boiling, oxygenated structures, whereas steam distillation selectively preserves thermally sensitive sesquiterpene fractions. Ultimately, these findings underscore that multivariate chemometric processing coupled with GC-MS characterization is an indispensable tool for objective quality control, chemical standardization, and industrial process optimization in the essential oil and phytopharmaceutical sectors. Based on our chemometric data, we provide two actionable industrial recommendations: (1) For high-value fragrances (e.g., *Lavandula angustifolia*), fresh biomass SD lines should be selected to preserve delicate, volatile monoterpene hydrocarbons and avoid the thermal degradation off-notes caused by water immersion (HD). (2) For phytopharmaceuticals targeting heavier bioactives (e.g., thunbergol in spruce or viridiflorol in sage), setups mimicking HD or utilizing extended steam processing of dehydrated biomass are preferred to maximize the recovery of less volatile sesquiterpene alcohols and diterpenes.

## Figures and Tables

**Table 1:** Comparison of plant material characteristics, distillation parameters, and EO yields obtained via laboratory-scale hydrodistillation and semi-industrial-scale steam distillation.

Species	*Picea abies*	*Achillea* *millefolium*	*Lavandula* *angustifolia*	*Hyssopus* *officinalis*	*Salvia* *officinalis*	*Mentha piperita*	*Mentha* *spicata*
**Plant** **family**	*Pinaceae*	*Asteraceae*	*Lamiaceae*	*Lamiaceae*	*Lamiaceae*	*Lamiaceae*	*Lamiaceae*
**Laboratory-scale HD**							
Plant material	ground dried needles and twigs	dried ground flowers	dried ground aerial parts	dried ground aerial parts	dried ground aerial parts	dried ground aerial parts	dried ground aerial parts
Distillation time	1.5 h	1.5 h	1.5 h	1.5 h	1.5 h	1.5 h	1.5 h
EO yield mL·kg^−1^ dried material *	3.71	4.29	15.71	4.29	11.14	21.43	14.29
**Semi-industrial-scale SD**							
Plant material	freshly ground needles and small branches	flowers and ca. 20 cm stems	fresh aerial parts	fresh aerial parts	fresh aerial parts	fresh aerial parts	fresh aerial parts
Distillation time	4–5 h	5–7 h	2 h	2 h	2 h	2 h	2 h
EO yield mL·kg^−1^ fresh material **	3.26	0.92	10.31	1.27	2.93	4.76	3.32

* EO yields were calculated for dried and ground raw plant material; ** EO yields were calculated for fresh raw plant material.

**Table 2:** The number of identified chemical compounds across the investigated plant species according to the extraction method.

Species	Steam Distillation ^1^	Hydrodistillation ^1^
*Lavandula angustifolia*	43	53
*Salvia officinalis*	46	48
*Hyssopus officinalis*	41	51
*Mentha piperita*	45	43
*Mentha spicata*	36	42
*Achillea millefolium*	51	58
*Picea abies*	52	69

^1^ The compounds identified by GC-MS analysis using the NIST mass spectral library.

**Table 3:** Chemical composition of *Picea abies* SEO and HEO.

RT (min)	Compound	Steam Distillation	Hydro- Distillation	RI^exp^	RI^lit^	Organoleptic Properties
7.911	tricyclene	0.64 ± 0.0000	-	934	932	
8.326	α-pinene	10.505 ± 0.0212	2.03 ± 0.0000	948	953	sharp warm resinous fresh pine
8.861	camphene	9.305 ± 0.0071	2.77 ± 0.0849	964	960	camphoraceous, cooling, pine woody with terpy nuances
9.377	sabinene	-	0.305 ± 0.0071	979	979	
9.709	β-pinene	10.405 ± 0.0919	2.645 ± 0.0495	988	987	dry woody resinous pine hay green eucalyptus camphoreous
10.032	β-myrcene	4.51 ± 0.0141	1.985 ± 0.0354	996	997	peppery terpene spicy balsam plastic
10.717	α-terpinen	-	0.1 ± 0.0141	1012	1011	
10.972	*p*-cymene	-	0.095 ± 0.0071	1017	1017	
11.330	β-phellandrene	9.59 ± 0.0566	2.445 ± 0.0778	1011	1011	mint terpentine;
11.415	D-limonene	5.635 ± 0.4172	3.935 ± 0.0778	1025	1025	citrus orange fresh sweet
11.463	eucalyptol	0.535 ± 0.1909	2.11 ± 0.0283	1026	1025	
11.688	*trans*-β-ocimene	0.11 ± 0.0000	0.12 ± 0.0000	1031	1031	
12.058	γ-terpinen	0.27 ± 0.0000	0.225 ± 0.0071	1037	1039	
12.745	α-terpinolen	0.025 ± 0.0071	0.465 ± 0.0071	1049	1050	
12.995	fenchone	0.065 ± 0.0071	-	1054	1052	
13.350	β-linalool	0.18 ± 0.0141	0.705 ± 0.0071	1059	1052	
13.582	3-methyl-3-butenyl isovalerate	-	0.145 ± 0.0071	1063	1062	
14.096	α-campholenal	-	0.18 ± 0.0141	1071	1072	
14.653	*trans*-pinocarveol	0.03 ± 0.0000	0.395 ± 0.0071	1079	1083	
14.842	camphor	3.4 ± 0.0000	3.905 ± 0.0071	1082	1083	
15.060	camphene hydrate	1.265 ± 0.0212	2.785 ± 0.0212	1085	1085	
15.169	pinocarvone	-	0.055 ± 0.0071	1086	1087	
15.227	linalool oxide (pyranoid)	-	0.185 ± 0.0071	1087	1087	
15.267	isoborneol	0.18 ± 0.0141	-	1088	1088	
15.637	borneol	4.27 ± 0.0000	4.595 ± 0.0212	1093	1091	pine woody camphoreous peppery
15.813	terpinen-4-ol	0.91 ± 0.0141	0.83 ± 0.0283	1095	1096	
16.053	*p*-cymen-8-ol	0.12 ± 0.0000	-	1098	1099	
16.287	α-terpineol	2.415 ± 0.0071	3.315 ± 0.0212	1202	1204	pine terpenic lilac citrus woody floral
17.029	nerol	-	4.85 ± 0.0424	1217	1217	
17.130	β-citronellol	0.38 ± 0.0141	-	1219	1219	
17.297	neral	-	0.225 ± 0.0071	1223	1219	
17.611	myrtanyl acetate	-	0.265 ± 0.0071	1229	1229	
17.649	carvone	0.05 ± 0.0000	-	1230	1230	
17.798	geraniol	-	7.515 ± 0.0212	1233	1231	
17.923	1-acetoxy-*p*-menth-3-one	0.13 ± 0.0000	-	1235	1236	
18.827	bornyl acetate	19.675 ± 0.1626	11.49 ± 0.1273	1252	1253	sweet balsamic woody fresh fir needle herbal
19.984	γ-pyronene	0.03 ± 0.0000	0.06 ± 0.0000	1272	1271	
20.149	2-hydroxycineole acetate	0.07 ± 0.0000	0.085 ± 0.0071	1275	1274	
20.381	α-terpenyl acetate	1.025 ± 0.0071	0.975 ± 0.0071	1279	1277	
20.503	α-longipinene	0.345 ± 0.0071	0.295 ± 0.0071	1281	1282	
20.832	dodecan-2-one	0.05 ± 0.0000	0.105 ± 0.0071	1286	1286	
21.000	ylangene	0.115 ± 0.0071	0.125 ± 0.0071	1289	1289	
21.110	cycloisosativene	-	0.465 ± 0.0212	1291	1292	
21.522	β-elemene	0.095 ± 0.0071	0.485 ± 0.0071	1297	1297	
21.846	β-guaiene	-	0.29 ± 0.0000	1403	1403	
21.907	α-gurjunene	0.305 ± 0.0071	-	1404	1404	
22.126	longifolene	2.48 ± 0.0000	2.22 ± 0.0141	1409	1410	sweet woody rose medicinal fir needle
22.340	β-caryophyllene	0.73 ± 0.0000	0.84 ± 0.0000	1414	1415	
22.622	*trans*-α-bergamotene	0.14 ± 0.0000	0.155 ± 0.0071	1420	1420	
22.816	β-sesquiphellandrene	0.12 ± 0.0000	0.165 ± 0.0071	1424	1420	
23.099	β-farnesene	0.935 ± 0.0071	1.17 ± 0.0141	1430	1430	
23.249	α-humulene	0.28 ± 0.0000	0.54 ± 0.0000	1434	1435	
23.408	β-cubebene	0.03 ± 0.0000	-	1437	1437	
23.460	bornyl butyrate	0.015 ± 0.0071	0.285 ± 0.0071	1438	1437	
23.770	plinol C	0.03 ± 0.0000	0.195 ± 0.0071	1444	1440	
23.847	α-curcumene	0.215 ± 0.0071	0.38 ± 0.0000	1446	1440	
24.206	γ-terpineol		0.25 ± 0.0141	1453	1453	
24.304	α-muurolene	0.275 ± 0.0071	-	1455	1455	
24.407	α-farnesene	0.545 ± 0.0071	0.66 ± 0.0000	1457	1458	
24.527	α-cedrene		0.415 ± 0.0071	1453	1453	
24.683	α-amorphene	0.375 ± 0.0071	1.78 ± 0.0000	1463	1462	
24.810	δ-cadinene	1.745 ± 0.0071	3.63 ± 0.0141	1465	1464	thyme herbal woody dry
25.103	cadina-1,4-diene		0.08 ± 0.0000	1471	1473	
25.803	nerolidol	0.065 ± 0.0071	1.085 ± 0.0071	1484	1485	
25.887	dodec-11-en-2-one	-	0.14 ± 0.0000	1486	1485	
26.256	(−)-spathulenol	0.105 ± 0.0071	1.005 ± 0.0071	1493	1493	
26.346	caryophyllene oxide	-	0.41 ± 0.0000	1494	1494	
26.847	*trans*-α-bisabolene epoxide	-	0.175 ± 0.0071	1605	1607	
27.000	tridec-3-ene	-	0.13 ± 0.0000	1609	1609	
27.103	cubenol	-	0.125 ± 0.0071	1611	1612	
27.390	di-epi-α-cedrene	-	0.27 ± 0.0000	1618	1612	
27.503	γ-eudesmol	-	0.95 ± 0.0141	1621	1619	
28.073	α-cadinol	0.415 ± 0.0071	6.68 ± 0.0990	1634	1633	
28.282	tetradec-11-en-1-ol acetate	-	0.335 ± 0.0212	1639	1639	
28.400	hexadec-3-ene	-	0.27 ± 0.0141	1641	1641	
28.619	phytol	-	0.13 ± 0.0000	1646	1646	
29.334	farnesol	-	1.47 ± 0.0141	1662	1658	
33.729	4-methylene-1-methyl-2-(2-methyl-1-propen-1-yl)-1-vinylcyclopheptane	0.715 ± 0.0071	1.575 ± 0.0071	1867	1867	
36.299	thunbergol	0.155 ± 0.0071	5.09 ± 0.0283	2033	2032	
	**Total**	96.01	96.16			
	Total Monoterpene Hydrocarbons	51.025	17.18			
	Total Sesquiterpene Hydrocarbons	8.73	13.965			
	Total Oxygenated Monoterpene Oxides	0.535	2.295			
	Total Oxygenated Monoterpene Alcohols	9.78	25.435			
	Total Oxygenated Monoterpene Ketones	3.515	3.96			
	Total Oxygenated Monoterpene Aldehydes	0	0.405			
	Total Oxygenated Monoterpene Esters	20.915	13.1			
	Total Oxygenated Sesquiterpene Alcohols	0.585	11.315			
	Total Oxygenated Sesquiterpene Oxides	0	0.585			
	Total Diterpene Alcohols	0.16	5.09			
	Other compounds	0.765	2.7			
	Shannon Diversity Index (H′)	2.29	3.02			
	Pielou’s Evenness (J′)	0.58	0.71			

**Table 4:** The comparative chemometric diagnostic metrics (R^2^X, R^2^Y, Q^2^) and primary VIP biomarker indicators for the six botanical volatile matrices under the LAS and LAH processing styles.

Plant Material	Total Variance (R^2^X %)	Explanatory Capacity (R^2^Y %)	Model Predictability (Q^2^ %)
*Mentha piperita*	0.881	0.997	0.994
*Achillea millefolium*	0.942	0.999	0.999
*Mentha spicata*	0.956	0.999	0.999
*Hyssopus officinalis*	0.880	0.999	0.997
*Picea abies*	0.988	0.999	0.999
*Salvia officinalis*	0.857	0.993	0.988

## Data Availability

The data presented in this study are available within the article and its associated Supplementary Materials.

## Supplementary Materials

The following supporting information can be downloaded at: https://www.mdpi.com/article/10.3390/molecules31122105/s1, Figures S1. GC-MS total ion chromatogram (TIC) of the chemical profile from *Picea abies* obtained by HD extraction. Figure S2. GC-MS total ion chromatogram (TIC) of the chemical profile from *Lavandula angustifolia* obtained by HD extraction. Figure S3. GC-MS total ion chromatogram (TIC) of the chemical profile from *Hyssopus officinalis* obtained by HD extraction. Figure S4. GC-MS total ion chromatogram (TIC) of the chemical profile from *Salvia officinalis* obtained by HD extraction. Figure S5. GC-MS total ion chromatogram (TIC) of the chemical profile from *Picea abies* obtained by SD extraction. Figure S6. GC-MS total ion chromatogram (TIC) of the chemical profile from *Salvia officinalis* obtained by SD extraction; Table S1. Chemical composition of *Salvia officinalis* SEO and HEO. Table S2. Chemical composition of *Mentha piperita* SEO and HEO. Table S3. Chemical composition of *Achillea millefolium* SEO and HEO. Table S4. Chemical composition of *Mentha spicata* SEO and HEO. Table S5. Chemical composition of *Hyssopus officinalis* SEO and HEO.

## Abbreviations

The following abbreviations are used in this manuscript:

EO	Essential oil
HD	Hydrodistillation
SD	Steam distillation
HEO	Essential oils obtained by hydrodistillation
SEO	Essential oils obtained by steam distillation
MT	Monoterpene
OMT	Oxygenated monoterpene
ST	Sesquiterpene
OST	Oxygenated sesquiterpene
