# Supplementary material for: Impact of Extraction Scale and Method on the Chemical Profile of Essential Oils: A Comparative Study Between Laboratory Hydrodistillation and Semi-Industrial Dry Steam Distillation
Source: Molecules. 2026 Jun 15;31(12):2105. doi: 10.3390/molecules31122105 (PMC13305851; doi:10.3390/molecules31122105)
Supplement: Supplementary file 1 [file molecules-31-02105-s001.zip › molecules-4374042-supplementary.pdf]

Impact of Extraction Scale and Method on the Chemical Profile of Essential Oils: A Comparative Study between Laboratory Hydrodistillation and Semi-Industrial Dry Steam Distillation

Norbert Léva [1], Emese Gál[1,*]

[1]Faculty of Chemistry and Chemical Engineering, Babeș-Bolyai University, Arany János No. 11, 400028 Cluj-Napoca, Romania;
*Correspondence: emese.gal@ubbcluj.ro

Table of Content:

Fig.S1. GC-MS total ion chromatogram (TIC) of the chemical profile from *Picea abies* obtained by HD extraction ... 2
Fig.S2. GC-MS total ion chromatogram (TIC) of the chemical profile from *Lavandula angustifolia* obtained by HD extraction ... 4
Fig.S3. GC-MS total ion chromatogram (TIC) of the chemical profile from *Hyssopus officinalis* obtained by HD extraction ... 6
Fig.S4. GC-MS total ion chromatogram (TIC) of the chemical profile from *Salvia officinalis* obtained by HD extraction ... 8
Fig.S5. GC-MS total ion chromatogram (TIC) of the chemical profile from *Picea abies* obtained by SD extraction ... 10
Fig.S6. GC-MS total ion chromatogram (TIC) of the chemical profile from *Salvia officinalis* obtained by SD extraction ... 11
Table S1. Chemical composition of *Salvia officinalis* SEO and HEO ... 13
Table S2. Chemical composition of *Mentha piperita* SEO and HEO ... 16
Table S3. Chemical composition of *Achillea millefolium* SEO and HEO ... 18
Table S4. Chemical composition of *Mentha spicata* SEO and HEO ... 21
Table S5. Chemical composition of *Hyssopus officinalis* SEO and HEO ... 23
Multivariate Statistical Analysis for *Mentha piperita* ... 24
Multivariate Statistical Analysis for *Achillea millefolium* ... 31
Multivariate Statistical Analysis for *Mentha spicata* ... 38
Multivariate Statistical Analysis for *Hyssopus officinalis* ... 45
Multivariate Statistical Analysis for *Picea abies* ... 52
Multivariate Statistical Analysis for *Salvia officinalis* ... 59

Figure S1: GC-MS total ion chromatogram (TIC) of the chemical profile from *Picea abies* obtained by HD extraction.

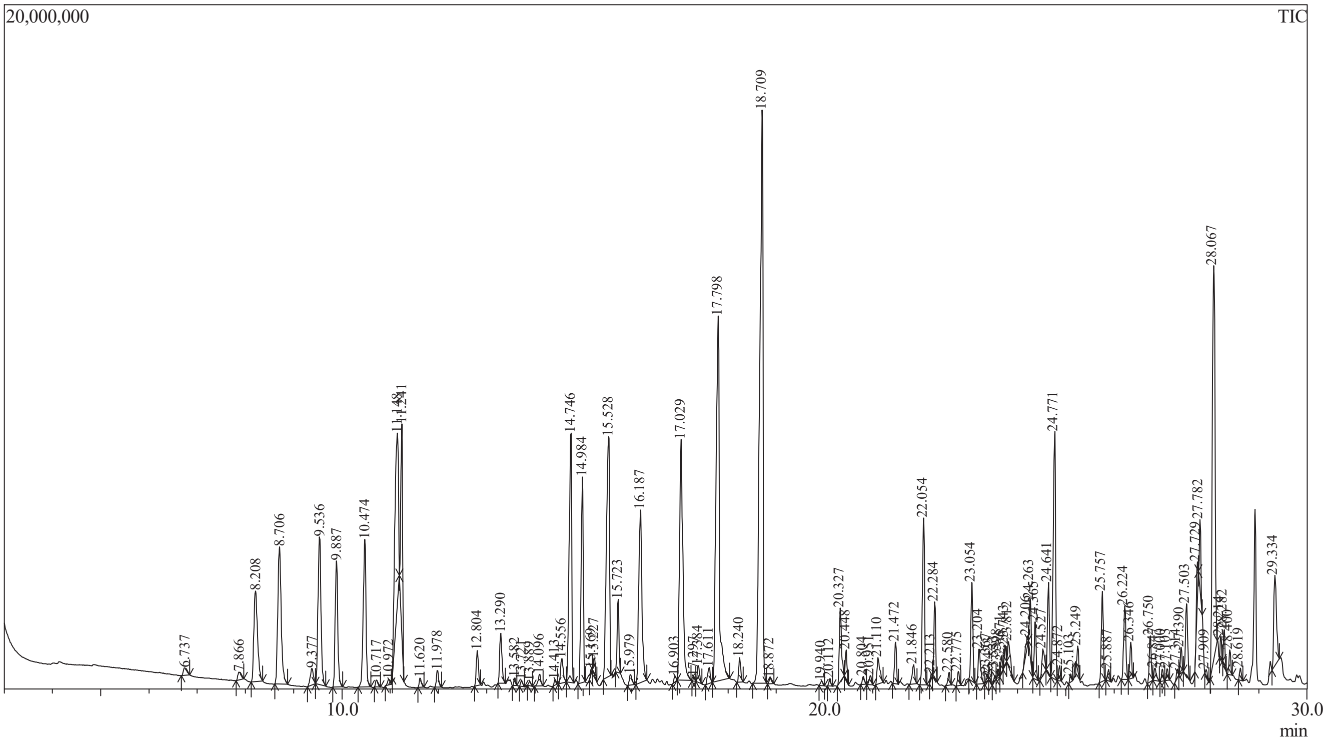

Peak Report TIC

Peak#	R.Time	Area	Area%	Height	Height%	A/H	Name
1	6.737	1043393	0.16	219636	0.13	4.75	
2	7.866	1032306	0.16	228728	0.13	4.51	
3	8.208	14094993	2.14	2644346	1.51	5.33	(1S)-2,6,6-Trimethylbicyclo[3.1.1]hept-2-ene
4	8.706	19632185	2.98	4006916	2.30	4.90	Camphene
5	9.377	2176371	0.33	481118	0.28	4.52	(1S)-2,6,6-Trimethylbicyclo[3.1.1]hept-2-ene
6	9.536	18604363	2.83	4321484	2.48	4.31	.beta.-Pinene
7	9.887	13951962	2.12	3681277	2.11	3.79	.beta.-Myrcene
8	10.474	17317621	2.63	4313529	2.47	4.01	1,3,7-Octatriene, 3,7-dimethyl-
9	10.717	642995	0.10	198709	0.11	3.24	1,3-Cyclohexadiene, 1-methyl-4-(1-methylethyl)-
10	10.972	603017	0.09	160254	0.09	3.76	Benzene, 1-methyl-4-(1-methylethyl)-
11	11.148	27641300	4.20	5028375	2.88	5.50	Limonene
12	11.241	14750090	2.24	6086605	3.49	2.42	Eucalyptol
13	11.620	851409	0.13	267034	0.15	3.19	1,3,7-Octatriene, 3,7-dimethyl-
14	11.978	1590606	0.24	487663	0.28	3.26	1,4-Cyclohexadiene, 1-methyl-4-(1-methylethyl)-
15	12.804	3189587	0.48	1019674	0.58	3.13	Cyclohexene, 1-methyl-4-(1-methylethylidene)-
16	13.290	4927082	0.75	1461327	0.84	3.37	1,6-Octadien-3-ol, 3,7-dimethyl-
17	13.582	545398	0.08	218534	0.13	2.50	Butanoic acid, 3-methyl-, 3-methyl-3-butenyl ester
18	13.721	508901	0.08	178375	0.10	2.85	Butanoic acid, 3-methyl-, 3-methyl-3-butenyl ester
19	13.889	370860	0.06	119088	0.07	3.11	Bicyclo[2.2.1]heptan-2-ol, 1,3,3-trimethyl-, (1R-endo)-

Peak#	R.Time	Area	Area%	Height	Height%	A/H	Name
20	14.096	1297804	0.20	337624	0.19	3.84	3-Cyclopentene-1-acetaldehyde, 2,2,3-trimethyl-
21	14.413	415812	0.06	158126	0.09	2.63	
22	14.556	2765189	0.42	720882	0.41	3.84	Bicyclo[3.1.1]heptan-3-ol, 6,6-dimethyl-2-methylene-, [1S-(1.alpha.,3.alpha.,5.alp
23	14.746	27092623	4.12	7284539	4.17	3.72	Bicyclo[2.2.1]heptan-2-one, 1,7,7-trimethyl-, (1S)-
24	14.984	19236769	2.92	6003544	3.44	3.20	Bicyclo[2.2.1]heptan-2-ol, 2,3,3-trimethyl-
25	15.169	378631	0.06	150874	0.09	2.51	2(10)-Pinen-3-one, (.+/-.)-
26	15.227	1277175	0.19	482226	0.28	2.65	2H-Pyran-3-ol, 6-ethenyltetrahydro-2,2,6-trimethyl-
27	15.528	31726459	4.82	7014503	4.02	4.52	Borneol
28	15.723	5635689	0.86	2137062	1.22	2.64	3-Cyclohexen-1-ol, 4-methyl-1-(1-methylethyl)-
29	15.979	1184112	0.18	300739	0.17	3.94	(-)-trans-Pinocarvyl acetate
30	16.187	23086695	3.51	5041914	2.89	4.58	p-menth-1-en-8-ol
31	16.903	514206	0.08	172099	0.10	2.99	2-Cyclohexen-1-ol, 2-methyl-5-(1-methylethenyl)-, cis-
32	17.029	33426331	5.08	7036188	4.03	4.75	2,6-Octadien-1-ol, 3,7-dimethyl-, (E)-
33	17.297	285317	0.04	124020	0.07	2.30	1,7,7-Trimethylbicyclo[2.2.1]hept-5-en-2-ol
34	17.384	1594858	0.24	489874	0.28	3.26	2,6-Octadienal, 3,7-dimethyl-, (Z)-
35	17.611	1795513	0.27	460264	0.26	3.90	Bicyclo[3.1.1]heptane-2-methanol, 6,6-dimethyl-, acetate
36	17.798	51976399	7.90	10661258	6.11	4.88	2,6-Octadien-1-ol, 3,7-dimethyl-, (E)-
37	18.240	2110463	0.32	719327	0.41	2.93	2,6-Octadienal, 3,7-dimethyl-, (E)-
38	18.709	78998500	12.01	16756670	9.60	4.71	Acetic acid, 1,7,7-trimethyl-bicyclo[2.2.1]hept-2-yl ester
39	18.872	538288	0.08	162785	0.09	3.31	2-Undecanone
40	19.940	410318	0.06	124880	0.07	3.29	1,5,5-Trimethyl-6-methylene-cyclohexene
41	20.112	587243	0.09	210951	0.12	2.78	2-Oxabicyclo[2.2.2]octan-6-ol, 1,3,3-trimethyl-, acetate
42	20.327	6795187	1.03	2149636	1.23	3.16	3-Cyclohexene-1-methanol, .alpha.,.alpha.,4-trimethyl-, acetate
43	20.448	2084194	0.32	866745	0.50	2.40	Tricyclo[5.4.0.0(2,8)]undec-9-ene, 2,6,6,9-tetramethyl-
44	20.804	710643	0.11	228860	0.13	3.11	2-Dodecanone
45	20.951	843443	0.13	262529	0.15	3.21	Ylangene
46	21.110	3153329	0.48	767545	0.44	4.11	(+)-Cycloisosativene
47	21.472	3361734	0.51	1213410	0.70	2.77	Cyclohexane, 1-ethenyl-1-methyl-2,4-bis(1-methylethenyl)-
48	21.846	2026219	0.31	559284	0.32	3.62	Azulene, 1,2,3,4,5,6,7,8-octahydro-1,4-dimethyl-7-(1-methylethylidene)-, (1S-cis)
49	22.054	15309873	2.33	4875131	2.79	3.14	1,4-Methanoazulene, decahydro-4,8,8-trimethyl-9-methylene-, [1S-(1.alpha.,3a.be
50	22.213	347941	0.05	140876	0.08	2.47	1H-3a,7-Methanoazulene, 2,3,4,7,8,8a-hexahydro-3,6,8,8-tetramethyl-, [3R-(3.alp
51	22.284	5847862	0.89	2252810	1.29	2.60	Bicyclo[7.2.0]undec-4-ene, 4,11,11-trimethyl-8-methylene-,[1R-(1R@,4Z,9S@)]-
52	22.580	1054794	0.16	371583	0.21	2.84	trans-.alpha.-Bergamotene
53	22.775	1108730	0.17	417030	0.24	2.66	Cyclohexene, 3-(1,5-dimethyl-4-hexenyl)-6-methylene-, [S-(R@,S@)]-
54	23.054	8183841	1.24	2888617	1.65	2.83	1,6,10-Dodecatriene, 7,11-dimethyl-3-methylene-, (E)-
55	23.204	3720103	0.57	1018237	0.58	3.65	.alpha.-Caryophyllene
56	23.367	554739	0.08	223108	0.13	2.49	
57	23.423	165281	0.03	100931	0.06	1.64	
58	23.533	379334	0.06	149159	0.09	2.54	
59	23.598	520214	0.08	258345	0.15	2.01	
60	23.671	1155635	0.18	505440	0.29	2.29	Naphthalene, 1,2,4a,5,6,8a-hexahydro-4,7-dimethyl-1-(1-methylethyl)-
61	23.743	1338307	0.20	581084	0.33	2.30	Cyclopentanol, 1,2-dimethyl-3-(1-methylethenyl)-, [1R-(1.alpha.,2.alpha.,3.alpha.)
62	23.812	2650533	0.40	724583	0.42	3.66	Benzene, 1-(1,5-dimethyl-4-hexenyl)-4-methyl-
63	24.206	1653419	0.25	297928	0.17	5.55	Cyclohexanol, 1-methyl-4-(1-methylethylidene)-
64	24.263	3931241	0.60	1694850	0.97	2.32	Naphthalene, 1,2,4a,5,6,8a-hexahydro-4,7-dimethyl-1-(1-methylethyl)-
65	24.365	4601617	0.70	1678427	0.96	2.74	.alpha.-Farnesene
66	24.527	2912740	0.44	773091	0.44	3.77	1H-3a,7-Methanoazulene, 2,3,4,7,8,8a-hexahydro-3,6,8,8-tetramethyl-, [3R-(3.alp
67	24.641	7188806	1.09	2666073	1.53	2.70	Naphthalene, 1,2,4a,5,6,8a-hexahydro-4,7-dimethyl-1-(1-methylethyl)-
68	24.771	25076203	3.81	7241155	4.15	3.46	Naphthalene, 1,2,3,5,6,8a-hexahydro-4,7-dimethyl-1-(1-methylethyl)-, (1S-cis)-
69	24.872	1084850	0.16	443465	0.25	2.45	1,6-Cyclodecadiene, 1-methyl-5-methylene-8-(1-methylethyl)-, [s-(E,E)]-
70	25.103	568553	0.09	233740	0.13	2.43	Naphthalene, 1,2,3,4,4a,7-hexahydro-1,6-dimethyl-4-(1-methylethyl)-
71	25.249	1540324	0.23	727302	0.42	2.12	.alpha.-Caryophyllene
72	25.757	7482392	1.14	2629680	1.51	2.85	1,6,10-Dodecatrien-3-ol, 3,7,11-trimethyl-, (E)-
73	25.887	941332	0.14	333042	0.19	2.83	11-Dodecen-2-one
74	26.224	6994979	1.06	2145044	1.23	3.26	(-)-Spathulenol
75	26.346	2816969	0.43	1059348	0.61	2.66	Caryophyllene oxide
76	26.750	3735260	0.57	1341524	0.77	2.78	Hydroxy-.alpha.-terpenyl acetate
77	26.847	1256249	0.19	359305	0.21	3.50	trans-Z-.alpha.-Bisabolene epoxide
78	27.000	893209	0.14	351072	0.20	2.54	3-Tridecene
79	27.103	925505	0.14	335974	0.19	2.75	Cubenol
80	27.314	661084	0.10	252097	0.14	2.62	Butanoic acid, 1,7,7-trimethylbicyclo[2.2.1]hept-2-yl ester, endo-
81	27.390	1871683	0.28	752368	0.43	2.49	Di-epi-.alpha.-cedrene-(I)
82	27.503	6649290	1.01	2118321	1.21	3.14	2-Naphthalenemethanol, 1,2,3,4,4a,5,6,7-octahydro-.alpha.,.alpha.,4a,8-tetramethy
83	27.729	3463594	0.53	1108897	0.64	3.12	Naphthalene, 1,2,3,5,6,8a-hexahydro-4,7-dimethyl-1-(1-methylethyl)-, (1S-cis)-
84	27.782	4001786	0.61	2057701	1.18	1.94	
85	27.909	382572	0.06	192693	0.11	1.99	
86	28.067	45808493	6.96	11507985	6.59	3.98	.alpha.-Cadinol
87	28.214	1290963	0.20	669726	0.38	1.93	Butanoic acid, 1,7,7-trimethylbicyclo[2.2.1]hept-2-yl ester, endo-
88	28.282	2229399	0.34	1034237	0.59	2.16	11-Tetradecen-1-ol, acetate, (Z)-
89	28.400	1907785	0.29	731339	0.42	2.61	3-Hexadecene, (Z)-
90	28.619	896986	0.14	349325	0.20	2.57	Phytol
91	29.334	10244518	1.56	2643502	1.51	3.88	2,6,10-Dodecatrien-1-ol, 3,7,11-trimethyl-, (E,E)-
92	33.700	10937301	1.66	3684287	2.11	2.97	Cycloheptane, 4-methylene-1-methyl-2-(2-methyl-1-propen-1-yl)-1-vinyl-
93	33.987	2725000	0.41	914204	0.52	2.98	1H-Cycloprop[e]azulene, decahydro-1,1,7-trimethyl-4-methylene-, [1aR-(1a.alpha
		657794871	100.00	174555666			

Figure S2: GC-MS total ion chromatogram (TIC) of the chemical profile from *Lavandula angustifolia* obtained by HD extraction

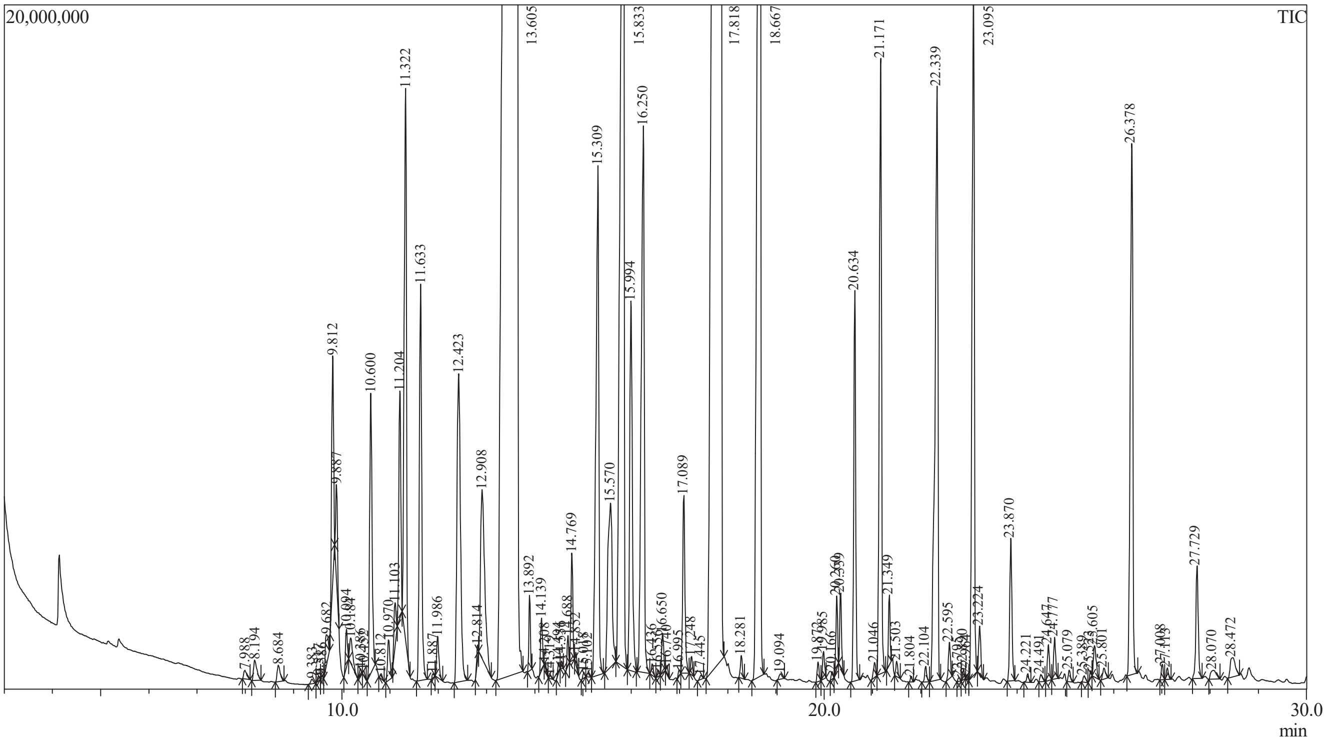

Peak Report TIC

Peak#	R.Time	Area	Area%	Height	Height%	A/H	Name
1	7.988	1063122	0.04	246126	0.06	4.32	Bicyclo[3.1.0]hex-2-ene, 2-methyl-5-(1-methylethyl)-
2	8.194	2846536	0.11	591785	0.15	4.81	Bicyclo[3.1.1]hept-2-ene, 2,6,6-trimethyl-, (.+/-.)-
3	8.684	2074847	0.08	478162	0.12	4.34	Camphene
4	9.383	523397	0.02	134896	0.03	3.88	Bicyclo[3.1.0]hexane, 4-methylene-1-(1-methylethyl)-
5	9.517	408326	0.02	143554	0.04	2.84	.beta.-Pinene
6	9.586	233987	0.01	106397	0.03	2.20	1-Octen-3-one
7	9.682	3728557	0.15	886973	0.22	4.20	1-Octen-3-ol
8	9.812	20020963	0.79	6620383	1.63	3.02	3-Octanone
9	9.887	7211262	0.28	2753995	0.68	2.62	.beta.-Myrcene
10	10.094	3123543	0.12	1121351	0.28	2.79	Butanoic acid, butyl ester
11	10.184	3079820	0.12	733115	0.18	4.20	3-Octanol
12	10.386	556447	0.02	196474	0.05	2.83	3-Undecyne
13	10.452	339193	0.01	141484	0.03	2.40	1,3,6-Octatriene, 3,7-dimethyl-, (Z)-
14	10.600	28513898	1.12	8187957	2.02	3.48	Acetic acid, hexyl ester
15	10.812	405617	0.02	143856	0.04	2.82	Benzene, 1-methyl-2-(1-methylethyl)-
16	10.970	4085246	0.16	1180062	0.29	3.46	Benzene, 1-methyl-4-(1-methylethyl)-
17	11.103	4311361	0.17	1303910	0.32	3.31	Limonene
18	11.204	19401627	0.76	6640760	1.64	2.92	Eucalyptol
19	11.322	52786353	2.07	16118481	3.98	3.27	(1S)-2,6,6-Trimethylbicyclo[3.1.1]hept-2-ene

Peak#	R.Time	Area	Area%	Height	Height%	A/H	Name
20	11.633	40282677	1.58	11587002	2.86	3.48	1,3,7-Octatriene, 3,7-dimethyl-
21	11.887	317685	0.01	124430	0.03	2.55	
22	11.986	3977397	0.16	1203087	0.30	3.31	1,4-Cyclohexadiene, 1-methyl-4-(1-methylethyl)-
23	12.423	45419677	1.78	9001941	2.22	5.05	2-Furanmethanol, 5-ethenyltetrahydro-.alpha.,.alpha.,5-trimethyl-, cis-
24	12.814	1099753	0.04	426741	0.11	2.58	Cyclohexene, 1-methyl-4-(1-methylethylidene)-
25	12.908	29396352	1.15	4942479	1.22	5.95	2-Furanmethanol, 5-ethenyltetrahydro-.alpha.,.alpha.,5-trimethyl-, trans-
26	13.605	805792303	31.62	50518597	12.46	15.95	1,6-Octadien-3-ol, 3,7-dimethyl-
27	13.892	4911072	0.19	2203895	0.54	2.23	3-Octanol, acetate
28	14.139	4102008	0.16	1532610	0.38	2.68	1,4-Hexadiene, 5-methyl-3-(1-methylethylidene)-
29	14.208	259908	0.01	120799	0.03	2.15	2,7-Octadien-4-ol, 2-methyl-6-methylene-, (S)-
30	14.319	219511	0.01	77746	0.02	2.82	
31	14.494	753275	0.03	310025	0.08	2.43	1,4-Pentadiene, 3-ethenyl-
32	14.556	433620	0.02	219126	0.05	1.98	Lilac aldehyde B
33	14.688	1954302	0.08	884114	0.22	2.21	Propanoic acid, 2-methyl-, hexyl ester
34	14.769	8426048	0.33	3145833	0.78	2.68	Bicyclo[2.2.1]heptan-2-one, 1,7,7-trimethyl-, (1S)-
35	14.852	690495	0.03	350886	0.09	1.97	2H-Pyran, 3,6-dihydro-4-methyl-2-(2-methyl-1-propenyl)-
36	15.018	455905	0.02	170302	0.04	2.68	Cyclohexanone, 5-methyl-2-(1-methylethyl)-
37	15.102	327375	0.01	110847	0.03	2.95	
38	15.309	62103811	2.44	14894568	3.67	4.17	4-Hexen-1-ol, 5-methyl-2-(1-methylethenyl)-, (R)-
39	15.570	30350903	1.19	4798236	1.18	6.33	Borneol
40	15.833	142240626	5.58	25191314	6.21	5.65	3-Cyclohexen-1-ol, 4-methyl-1-(1-methylethyl)-
41	15.994	40823234	1.60	10807564	2.67	3.78	Butanoic acid, hexyl ester
42	16.250	69256508	2.72	15967030	3.94	4.34	p-menth-1-en-8-ol
43	16.436	957419	0.04	330108	0.08	2.90	2,6-Dimethyl-3,5,7-octatriene-2-ol, ,E,E-
44	16.556	560556	0.02	193637	0.05	2.89	Acetic acid, octyl ester
45	16.650	3437926	0.13	1154143	0.28	2.98	2,6-Dimethyl-3,5,7-octatriene-2-ol, ,E,E-
46	16.740	517712	0.02	238689	0.06	2.17	2,6-Octadiene, 4,5-dimethyl-
47	16.995	420465	0.02	156352	0.04	2.69	2-Cyclohexen-1-ol, 2-methyl-5-(1-methylethenyl)-, cis-
48	17.089	17116863	0.67	5190597	1.28	3.30	2,6-Octadien-1-ol, 3,7-dimethyl-, (Z)-
49	17.248	1417714	0.06	530057	0.13	2.67	Butanoic acid, 2-methyl-, hexyl ester
50	17.445	421919	0.02	118355	0.03	3.56	Pulegone
51	17.818	536282215	21.04	48524184	11.97	11.05	1,6-Octadien-3-ol, 3,7-dimethyl-, acetate
52	18.281	1935192	0.08	667217	0.16	2.90	Myrcenylacetat
53	18.667	162930363	6.39	33457662	8.25	4.87	2,6-Octadien-1-ol, 3,7-dimethyl-, acetate, (Z)-
54	19.094	641999	0.03	154062	0.04	4.17	
55	19.872	1537008	0.06	570086	0.14	2.70	Cyclobutanecarboxylic acid, hexyl ester
56	19.985	2382282	0.09	814928	0.20	2.92	cis-3-Hexenyl iso-butyrate
57	20.166	578323	0.02	221133	0.05	2.62	1,3,6-Heptatriene, 2,5,5-trimethyl-
58	20.260	5184466	0.20	2144146	0.53	2.42	1-Hydroxylinalool
59	20.339	5557934	0.22	2179157	0.54	2.55	1-Hydroxylinalool
60	20.634	32236771	1.26	11410551	2.81	2.83	2,6-Octadien-1-ol, 3,7-dimethyl-, acetate, (Z)-
61	21.046	1406596	0.06	433700	0.11	3.24	7-Oxabicyclo[4.1.0]heptane, 1-methyl-4-(2-methyloxiranyl)-
62	21.171	58527635	2.30	17989882	4.44	3.25	2,6-Octadien-1-ol, 3,7-dimethyl-, acetate, (E)-
63	21.349	4906791	0.19	2039207	0.50	2.41	Hexanoic acid, hexyl ester
64	21.503	1251588	0.05	447438	0.11	2.80	1,6,10-Dodecatrien-3-ol, 3,7,11-trimethyl-, [S-(Z)]-
65	21.804	423051	0.02	165369	0.04	2.56	1,3-Cyclohexadiene, 5-(1,5-dimethyl-4-hexenyl)-2-methyl-, [S-(R@,S@)]-
66	22.104	1359020	0.05	452607	0.11	3.00	
67	22.339	73952854	2.90	17356256	4.28	4.26	Bicyclo[7.2.0]undec-4-ene, 4,11,11-trimethyl-8-methylene-,[1R-(1R@,4Z,9S@)]-
68	22.595	3028570	0.12	1054905	0.26	2.87	trans-.alpha.-Bergamotene
69	22.795	324683	0.01	123385	0.03	2.63	Cyclohexene, 3-(1,5-dimethyl-4-hexenyl)-6-methylene-, [S-(R@,S@)]-
70	22.890	769062	0.03	302823	0.07	2.54	1,6,10-Dodecatriene, 7,11-dimethyl-3-methylene-, (E)-
71	22.964	294877	0.01	141532	0.03	2.08	Bicyclo[2.2.1]heptane, 2-methyl-3-methylene-2-(4-methyl-3-pentenyl)-, (1S-endo)
72	23.095	68652261	2.69	19856077	4.90	3.46	1,6,10-Dodecatriene, 7,11-dimethyl-3-methylene-, (E)-
73	23.224	4216081	0.17	1416031	0.35	2.98	.alpha.-Caryophyllene
74	23.870	13320356	0.52	4163941	1.03	3.20	1,6-Cyclodecadiene, 1-methyl-5-methylene-8-(1-methylethyl)-, [s-(E,E)]-
75	24.221	733563	0.03	246226	0.06	2.98	.gamma.-Elemene
76	24.491	737403	0.03	208451	0.05	3.54	Cyclohexene, 1-methyl-4-(5-methyl-1-methylene-4-hexenyl)-, (S)-
77	24.647	3121027	0.12	1054998	0.26	2.96	Naphthalene, 1,2,4a,5,6,8a-hexahydro-4,7-dimethyl-1-(1-methylethyl)-
78	24.777	3599736	0.14	1216608	0.30	2.96	Tricyclo[2.2.1.0(2,6)]heptane-3-methanol, 2,3-dimethyl-
79	25.079	1050749	0.04	335923	0.08	3.13	
80	25.389	703521	0.03	186739	0.05	3.77	1,5-Cyclodecadiene, 1,5-dimethyl-8-(1-methylethylidene)-, (E,E)-
81	25.523	508610	0.02	216965	0.05	2.34	
82	25.605	3612446	0.14	918295	0.23	3.93	Caryophyllene oxide
83	25.801	1316474	0.05	319455	0.08	4.12	1,6,10-Dodecatrien-3-ol, 3,7,11-trimethyl-, [S-(Z)]-
84	26.378	58437268	2.29	15520783	3.83	3.77	Caryophyllene oxide
85	27.008	1289292	0.05	448926	0.11	2.87	3-Oxatricyclo[4.1.1.0(2,4)]octane, 2,7,7-trimethyl-
86	27.113	1017853	0.04	335292	0.08	3.04	Epiglobulol
87	27.729	11488899	0.45	3291497	0.81	3.49	1-Naphthalenol, 1,2,3,4,4a,7,8,8a-octahydro-1,6-dimethyl-4-(1-methylethyl)-, [1R
88	28.070	2055036	0.08	253284	0.06	8.11	
89	28.472	4161352	0.16	564382	0.14	7.37	
		2548692328	100.00	405434934			

Figure S3: GC-MS total ion chromatogram (TIC) of the chemical profile from *Hyssopus officinalis* obtained by HD.

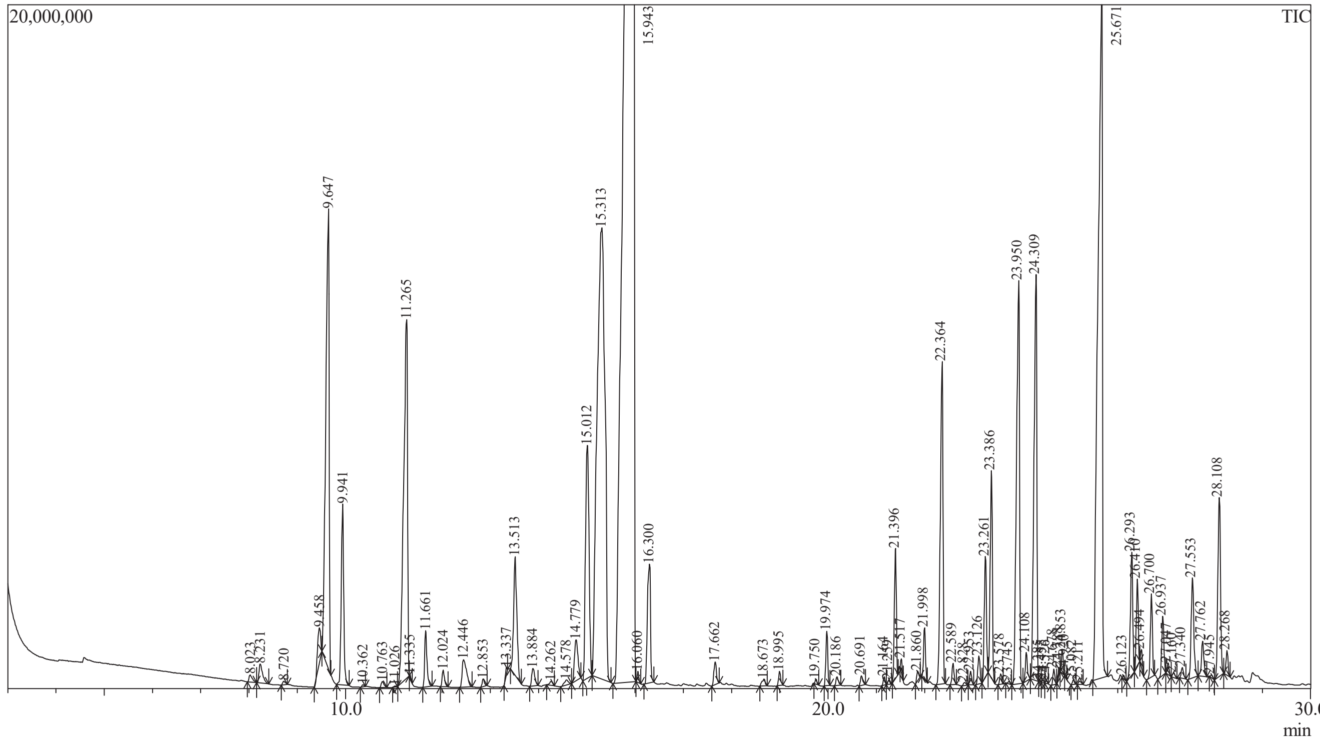

Peak Report TIC

Peak#	R.Time	Area	Area%	Height	Height%	A/H	Name
1	8.023	987491	0.08	210112	0.11	4.70	Bicyclo[3.1.0]hex-2-ene, 2-methyl-5-(1-methylethyl)-
2	8.231	2863113	0.23	552748	0.29	5.18	Bicyclo[3.1.1]hept-2-ene, 2,6,6-trimethyl-, (.+/-.)-
3	8.720	393952	0.03	99604	0.05	3.96	Camphene
4	9.458	4572739	0.37	1015814	0.53	4.50	Bicyclo[3.1.0]hexane, 4-methylene-1-(1-methylethyl)-
5	9.647	64480959	5.21	13448003	6.95	4.79	.beta.-Pinene
6	9.941	18846407	1.52	5286457	2.73	3.57	.beta.-Myrcene
7	10.362	166457	0.01	56720	0.03	2.93	1,3,7-Octatriene, 3,7-dimethyl-
8	10.763	656391	0.05	167434	0.09	3.92	Cyclohexene, 1-methyl-4-(1-methylethylidene)-
9	11.026	108823	0.01	32213	0.02	3.38	Benzene, 1-methyl-4-(1-methylethyl)-
10	11.265	59652528	4.82	10496089	5.43	5.68	Bicyclo[3.1.0]hexane, 4-methylene-1-(1-methylethyl)-
11	11.335	78503	0.01	65395	0.03	1.20	(1S)-2,6,6-Trimethylbicyclo[3.1.1]hept-2-ene
12	11.661	5496482	0.44	1645904	0.85	3.34	1,3,7-Octatriene, 3,7-dimethyl-
13	12.024	1656340	0.13	486677	0.25	3.40	1,4-Cyclohexadiene, 1-methyl-4-(1-methylethyl)-
14	12.446	5082727	0.41	803868	0.42	6.32	Terpineol, cis-.beta.-
15	12.853	721234	0.06	218145	0.11	3.31	Cyclohexene, 1-methyl-4-(1-methylethylidene)-
16	13.337	545833	0.04	186092	0.10	2.93	1,6-Octadien-3-ol, 3,7-dimethyl-
17	13.513	15919741	1.29	3469408	1.79	4.59	1,6-Octadien-3-ol, 3,7-dimethyl-
18	13.884	2279905	0.18	516623	0.27	4.41	Bicyclo[3.1.0]hexan-3-one, 4-methyl-1-(1-methylethyl)-, [1S-(1.alpha.,4.beta.,5.alpha.)]-
19	14.262	480634	0.04	119972	0.06	4.01	2-Cyclohexen-1-ol, 1-methyl-4-(1-methylethyl)-, trans-

Peak#	R.Time	Area	Area%	Height	Height%	A/H	Name
20	14.578	870133	0.07	144972	0.07	6.00	Bicyclo[3.1.1]heptane, 2,6,6-trimethyl-
21	14.779	5985815	0.48	1192682	0.62	5.02	Bicyclo[2.2.1]heptan-2-one, 1,7,7-trimethyl-, (.+/-.)-
22	15.012	34811274	2.81	6804638	3.52	5.12	Cyclohexene, 4-isopropenyl-1-methoxymethoxymethyl-
23	15.313	149366186	12.07	13200084	6.83	11.32	Bicyclo[3.1.1]heptan-3-one, 2,6,6-trimethyl-, (1.alpha.,2.beta.,5.alpha.)-
24	15.943	436294454	35.26	29869191	15.45	14.61	Bicyclo[3.1.1]heptan-3-one, 2,6,6-trimethyl-, (1.alpha.,2.beta.,5.alpha.)-
25	16.060	728666	0.06	323086	0.17	2.26	2-Cyclohexen-1-one, 4-(1-methylethyl)-
26	16.300	15570867	1.26	3469823	1.79	4.49	Bicyclo[3.1.1]hept-2-ene-2-methanol, 6,6-dimethyl-
27	17.662	2458098	0.20	660598	0.34	3.72	
28	18.673	941193	0.08	203954	0.11	4.61	Acetic acid, 1,7,7-trimethyl-bicyclo[2.2.1]hept-2-yl ester
29	18.995	1241371	0.10	410614	0.21	3.02	3-Octen-5-yne, 2,7-dimethyl-, (E)-
30	19.750	367010	0.03	148558	0.08	2.47	(-)-Myrtenyl acetate
31	19.974	4436132	0.36	1589710	0.82	2.79	1,5-Cyclodecadiene, 1,5-dimethyl-8-(1-methylethylidene)-, (E,E)-
32	20.186	875542	0.07	255991	0.13	3.42	
33	20.691	937160	0.08	276708	0.14	3.39	2,6-Octadien-1-ol, 3,7-dimethyl-, acetate, (Z)-
34	21.164	629790	0.05	238870	0.12	2.64	Copaene
35	21.259	328179	0.03	123874	0.06	2.65	2-Buten-1-one, 1-(2,6,6-trimethyl-1,3-cyclohexadien-1-yl)-
36	21.396	10946453	0.88	3799193	1.96	2.88	Cyclobuta[1,2:3,4]dicyclopentene, decahydro-3a-methyl-6-methylene-1-(1-methyl
37	21.517	766973	0.06	395161	0.20	1.94	Cyclohexane, 1-ethenyl-1-methyl-2,4-bis(1-methylethenyl)-, (1.alpha.,2.beta.,4.bet
38	21.860	829561	0.07	271203	0.14	3.06	Benzene, 1,2-dimethoxy-4-(2-propenyl)-
39	21.998	4098941	0.33	1507374	0.78	2.72	1H-Cycloprop[e]azulene, 1a,2,3,4,4a,5,6,7b-octahydro-1,1,4,7-tetramethyl-, [1aR-
40	22.364	36236583	2.93	9428805	4.88	3.84	Bicyclo[7.2.0]undec-4-ene, 4,11,11-trimethyl-8-methylene-,[1R-(1R@,4Z,9S@)]-
41	22.589	1829112	0.15	625517	0.32	2.92	1,6-Cyclodecadiene, 1-methyl-5-methylene-8-(1-methylethyl)-, [s-(E,E)]-
42	22.828	335965	0.03	93371	0.05	3.60	Bicyclo[7.2.0]undec-4-ene, 4,11,11-trimethyl-8-methylene-,[1R-(1R@,4Z,9S@)]-
43	22.953	1121315	0.09	392780	0.20	2.85	1H-Cycloprop[e]azulene, decahydro-1,1,7-trimethyl-4-methylene-, [1aR-(1a.alpha
44	23.126	2645452	0.21	811774	0.42	3.26	Cyclohexene, 1-methyl-4-(5-methyl-1-methylene-4-hexenyl)-, (S)-
45	23.261	10880356	0.88	3522526	1.82	3.09	.alpha.-Caryophyllene
46	23.386	18813100	1.52	6059327	3.13	3.10	Aromadendrene
47	23.578	830519	0.07	302693	0.16	2.74	1,6-Cyclodecadiene, 1-methyl-5-methylene-8-(1-methylethyl)-, [s-(E,E)]-
48	23.745	180050	0.01	68392	0.04	2.63	
49	23.950	50510348	4.08	11796579	6.10	4.28	1,6-Cyclodecadiene, 1-methyl-5-methylene-8-(1-methylethyl)-, [s-(E,E)]-
50	24.108	2545099	0.21	836646	0.43	3.04	Azulene, 1,2,3,5,6,7,8,8a-octahydro-1,4-dimethyl-7-(1-methylethenyl)-, [1S-(1.alp
51	24.309	48178115	3.89	11880673	6.14	4.06	.gamma.-Elemene
52	24.385	81371	0.01	56361	0.03	1.44	Naphthalene, decahydro-1,6-bis(methylene)-4-(1-methylethyl)-, (4.alpha.,4a.alpha
53	24.455	191662	0.02	87271	0.05	2.20	
54	24.526	215768	0.02	93289	0.05	2.31	Cyclohexene, 1-methyl-4-(5-methyl-1-methylene-4-hexenyl)-, (S)-
55	24.678	1232121	0.10	452731	0.23	2.72	Naphthalene, 1,2,4a,5,6,8a-hexahydro-4,7-dimethyl-1-(1-methylethyl)-
56	24.788	580945	0.05	223231	0.12	2.60	Naphthalene, 1,2,3,5,6,8a-hexahydro-4,7-dimethyl-1-(1-methylethyl)-, (1S-cis)-
57	24.853	1660778	0.13	706831	0.37	2.35	
58	24.920	118597	0.01	84269	0.04	1.41	Cyclohexene, 3-(1,5-dimethyl-4-hexenyl)-6-methylene-, [S-(R@,S@)]-
59	25.082	365708	0.03	127187	0.07	2.88	
60	25.211	380832	0.03	102132	0.05	3.73	
61	25.671	127810146	10.33	20154758	10.42	6.34	Cyclohexanemethanol, 4-ethenyl-.alpha.,.alpha.,4-trimethyl-3-(1-methylethenyl)-, [
62	26.123	228346	0.02	103775	0.05	2.20	4aH-Cycloprop[e]azulen-4a-ol, decahydro-1,1,4,7-tetramethyl-, [1aR-(1a.alpha.,4.
63	26.293	11803772	0.95	3596636	1.86	3.28	(-)-Spathulenol
64	26.410	7123323	0.58	2542016	1.31	2.80	Caryophyllene oxide
65	26.494	1270945	0.10	524360	0.27	2.42	Globulol
66	26.700	7285840	0.59	2429307	1.26	3.00	VERIDIFLOROL
67	26.937	5344168	0.43	1722357	0.89	3.10	Ledol
68	27.047	880914	0.07	338744	0.18	2.60	
69	27.160	468732	0.04	135546	0.07	3.46	Cubenol
70	27.340	1028047	0.08	318422	0.16	3.23	2-Naphthalenemethanol, 1,2,3,4,4a,5,6,8a-octahydro-.alpha.,.alpha.,4a,8-tetrameth
71	27.553	11294667	0.91	2925255	1.51	3.86	2-Naphthalenemethanol, 1,2,3,4,4a,5,6,7-octahydro-.alpha.,.alpha.,4a,8-tetramethy
72	27.762	3713932	0.30	1026853	0.53	3.62	.tau.-Cadinol
73	27.945	230861	0.02	82073	0.04	2.81	Tricyclo[4.4.0.0(2,7)]dec-8-ene-3-methanol, .alpha.,.alpha.,6,8-tetramethyl-, stere
74	28.108	20255465	1.64	5229168	2.70	3.87	2-Naphthalenemethanol, decahydro-.alpha.,.alpha.,4a-trimethyl-8-methylene-, [2R
75	28.268	2110291	0.17	716515	0.37	2.95	Cyclohexanemethanol, 4-ethenyl-.alpha.,.alpha.,4-trimethyl-3-(1-methylethenyl)-, [
		1237257302	100.00	193361732			

Figure S4: GC-MS total ion chromatogram (TIC) of the chemical profile from *Salvia officinalis* obtained by HD.

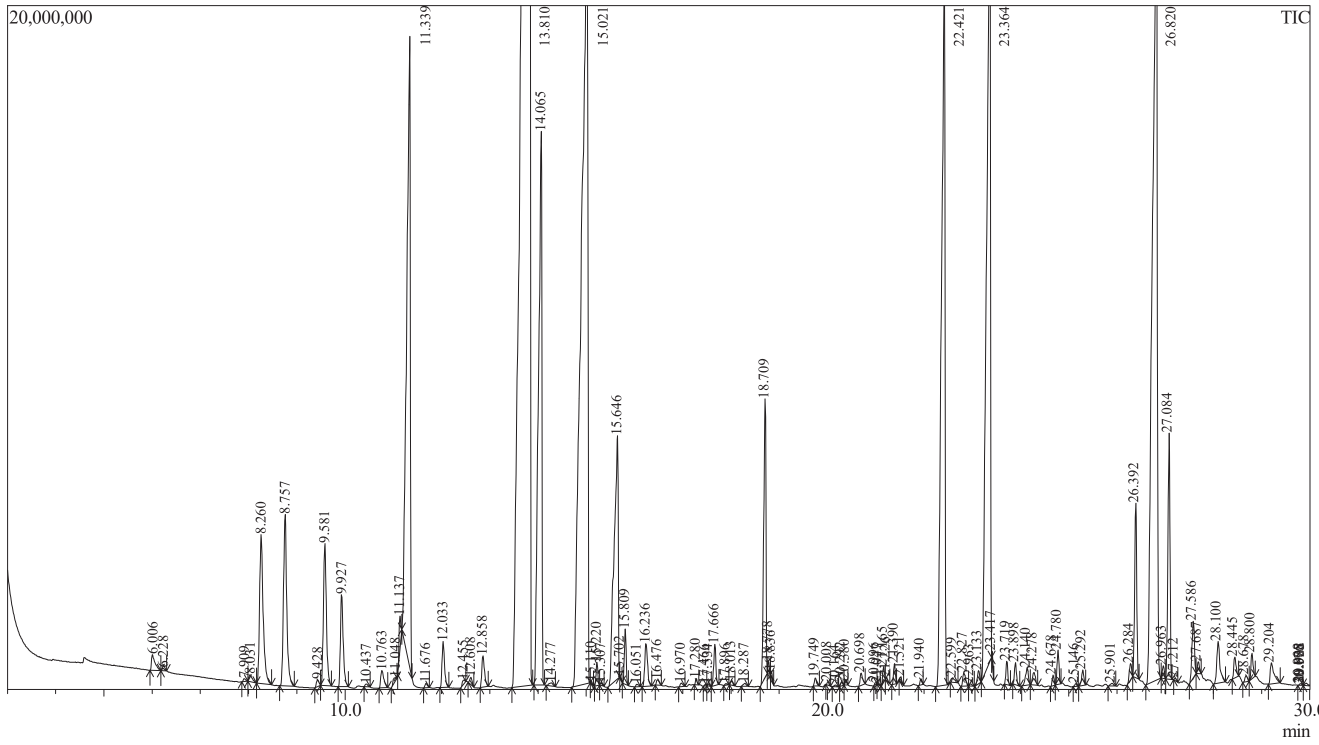

Peak Report TIC

Peak#	R.Time	Area	Area%	Height	Height%	A/H	Name
1	6.006	2302450	0.14	464636	0.18	4.96	
2	6.228	229713	0.01	71988	0.03	3.19	1,6-Octadiene, 3,7-dimethyl-
3	7.909	285222	0.02	83795	0.03	3.40	Tricyclo[2.2.1.0(2,6)]heptane, 1,7,7-trimethyl-
4	8.031	913304	0.06	204414	0.08	4.47	Bicyclo[3.1.0]hex-2-ene, 2-methyl-5-(1-methylethyl)-
5	8.260	22421972	1.40	4358477	1.69	5.14	Bicyclo[3.1.1]hept-2-ene, 2,6,6-trimethyl-, (.+/-.)-
6	8.757	23247723	1.45	5006494	1.94	4.64	Camphene
7	9.428	736594	0.05	198439	0.08	3.71	Bicyclo[3.1.0]hexane, 4-methylene-1-(1-methylethyl)-
8	9.581	17831602	1.12	4154065	1.61	4.29	.beta.-Pinene
9	9.927	10813564	0.68	2685767	1.04	4.03	.beta.-Myrcene
10	10.437	302274	0.02	80663	0.03	3.75	.alpha.-Phellandrene
11	10.763	2222721	0.14	505708	0.20	4.40	Cyclohexene, 4-methyl-3-(1-methylethylidene)-
12	11.048	620926	0.04	82245	0.03	7.55	Benzene, 1-methyl-4-(1-methylethyl)-
13	11.137	2825700	0.18	980962	0.38	2.88	Limonene
14	11.339	95590143	5.98	18433352	7.16	5.19	Eucalyptol
15	11.676	425368	0.03	124303	0.05	3.42	1,3,7-Octatriene, 3,7-dimethyl-
16	12.033	4848437	0.30	1350946	0.52	3.59	1,4-Cyclohexadiene, 1-methyl-4-(1-methylethyl)-
17	12.455	469078	0.03	126611	0.05	3.70	
18	12.608	651505	0.04	211495	0.08	3.08	Terpineol, cis-.beta.-
19	12.858	3559578	0.22	909463	0.35	3.91	Cyclohexene, 1-methyl-4-(1-methylethylidene)-

Peak#	R.Time	Area	Area%	Height	Height%	A/H	Name
20	13.810	390726491	24.44	29098155	11.30	13.43	Thujone
21	14.065	80306249	5.02	16187771	6.28	4.96	Bicyclo[3.1.0]hexan-3-one, 4-methyl-1-(1-methylethyl)-, [1S-(1.alpha.,4.beta.,5.al
22	14.277	506367	0.03	94685	0.04	5.35	Bicyclo[3.1.0]hexan-3-ol, 4-methyl-1-(1-methylethyl)-
23	15.021	207374656	12.97	21904197	8.50	9.47	Bicyclo[2.2.1]heptan-2-one, 1,7,7-trimethyl-, (1S)-
24	15.110	127513	0.01	73884	0.03	1.73	Bicyclo[2.2.1]heptan-2-ol, 2,3,3-trimethyl-
25	15.220	1707782	0.11	677772	0.26	2.52	Bicyclo[3.1.1]heptan-3-one, 2,6,6-trimethyl-, (1.alpha.,2.beta.,5.alpha.)-
26	15.307	172085	0.01	69838	0.03	2.46	Isoborneol
27	15.646	41712915	2.61	7117071	2.76	5.86	Borneol
28	15.702	68067	0.00	64422	0.03	1.06	Bicyclo[3.1.1]heptan-3-one, 2,6,6-trimethyl-, (1.alpha.,2.beta.,5.alpha.)-
29	15.809	4158912	0.26	1622112	0.63	2.56	3-Cyclohexen-1-ol, 4-methyl-1-(1-methylethyl)-
30	16.051	280278	0.02	72168	0.03	3.88	Benzenemethanol, .alpha.,.alpha.,4-trimethyl-
31	16.236	5030416	0.31	1239350	0.48	4.06	p-menth-1-en-8-ol
32	16.476	587212	0.04	152673	0.06	3.85	Bicyclo[3.1.0]hexan-3-ol, 4-methyl-1-(1-methylethyl)-
33	16.970	399415	0.02	106870	0.04	3.74	2-Cyclohexen-1-ol, 2-methyl-5-(1-methylethenyl)-, cis-
34	17.280	436646	0.03	183218	0.07	2.38	Pentanoic acid, 4-hexen-1-yl ester
35	17.466	101511	0.01	42738	0.02	2.38	
36	17.534	139102	0.01	58203	0.02	2.39	
37	17.666	3828623	0.24	1210154	0.47	3.16	
38	17.896	163613	0.01	40798	0.02	4.01	2,6-Octadien-1-ol, 3,7-dimethyl-, (E)-
39	18.013	340525	0.02	119232	0.05	2.86	
40	18.287	347566	0.02	80289	0.03	4.33	
41	18.709	26945348	1.69	8142334	3.16	3.31	Acetic acid, 1,7,7-trimethyl-bicyclo[2.2.1]hept-2-yl ester
42	18.778	682172	0.04	362656	0.14	1.88	(-)-Myrtenyl acetate
43	18.836	304730	0.02	194423	0.08	1.57	6,8-Nonadien-2-one, 8-methyl-5-(1-methylethyl)-, (E)-
44	19.749	736243	0.05	235195	0.09	3.13	(-)-Myrtenyl acetate
45	20.008	263247	0.02	100009	0.04	2.63	2-Cyclohexen-1-ol, 2-methyl-5-(1-methylethenyl)-, acetate, (1R-cis)-
46	20.164	181590	0.01	49676	0.02	3.66	2-Oxabicyclo[2.2.2]octan-6-ol, 1,3,3-trimethyl-, acetate
47	20.292	284549	0.02	105694	0.04	2.69	(-)-trans-Pinocarvyl acetate
48	20.380	398168	0.02	160340	0.06	2.48	.alpha.-Cubebene
49	20.698	1292523	0.08	356270	0.14	3.63	2,6-Octadien-1-ol, 3,7-dimethyl-, acetate
50	20.996	146918	0.01	58877	0.02	2.50	Ylangene
51	21.047	40308	0.00	20716	0.01	1.95	
52	21.165	1100901	0.07	445935	0.17	2.47	Copaene
53	21.226	187181	0.01	91706	0.04	2.04	2,6-Octadien-1-ol, 3,7-dimethyl-, acetate, (E)-
54	21.390	1543064	0.10	601596	0.23	2.56	Cyclobuta[1,2:3,4]dicyclopentene, decahydro-3a-methyl-6-methylene-1-(1-methyl
55	21.521	367707	0.02	157603	0.06	2.33	
56	21.940	491588	0.03	174664	0.07	2.81	Bicyclo[7.2.0]undec-4-ene, 4,11,11-trimethyl-8-methylene-,[1R-(1R@,4Z,9S@)]-
57	22.421	105276849	6.59	20378476	7.91	5.17	Caryophyllene
58	22.599	503522	0.03	133405	0.05	3.77	1H-Cyclopenta[1,3]cyclopropa[1,2]benzene, octahydro-7-methyl-3-methylene-4-(
59	22.827	1588694	0.10	395610	0.15	4.02	Aromadendrene
60	22.963	229117	0.01	60465	0.02	3.79	1H-Cycloprop[e]azulene, decahydro-1,1,7-trimethyl-4-methylene-, [1aR-(1a.alpha
61	23.133	1288465	0.08	389708	0.15	3.31	3,7-Nonadien-2-ol, 4,8-dimethyl-
62	23.364	125903762	7.88	22692952	8.81	5.55	.alpha.-Caryophyllene
63	23.417	405266	0.03	401327	0.16	1.01	Aromadendrene
64	23.719	1911945	0.12	692384	0.27	2.76	Naphthalene, 1,2,4a,5,6,8a-hexahydro-4,7-dimethyl-1-(1-methylethyl)-
65	23.898	1871776	0.12	648446	0.25	2.89	1,6-Cyclodecadiene, 1-methyl-5-methylene-8-(1-methylethyl)-, [s-(E,E)]-
66	24.140	2114542	0.13	499558	0.19	4.23	1H-Cycloprop[e]azulene, 1a,2,3,5,6,7,7a,7b-octahydro-1,1,4,7-tetramethyl-, [1aR-
67	24.278	1555575	0.10	368183	0.14	4.23	1H-Cycloprop[e]azulene, decahydro-1,1,7-trimethyl-4-methylene-
68	24.678	815137	0.05	304414	0.12	2.68	Naphthalene, 1,2,4a,5,6,8a-hexahydro-4,7-dimethyl-1-(1-methylethyl)-
69	24.780	2780341	0.17	998551	0.39	2.78	Naphthalene, 1,2,3,5,6,8a-hexahydro-4,7-dimethyl-1-(1-methylethyl)-, (1S-cis)-
70	25.146	222847	0.01	76347	0.03	2.92	Naphthalene, 1,2,3,4,4a,7-hexahydro-1,6-dimethyl-4-(1-methylethyl)-
71	25.292	1447366	0.09	452475	0.18	3.20	Cyclohexane, 1-methyl-2,4-bis(1-methylethenyl)-, (1.alpha.,2.beta.,4.beta.)-
72	25.901	335052	0.02	74621	0.03	4.49	1,6,10-Dodecatrien-3-ol, 3,7,11-trimethyl-, [S-(Z)]-
73	26.284	1561456	0.10	463872	0.18	3.37	(-)-Spathulenol
74	26.392	15276498	0.96	5107424	1.98	2.99	Caryophyllene oxide
75	26.820	156955350	9.82	22561507	8.76	6.96	VERIDIFLOROL
76	26.963	948807	0.06	362651	0.14	2.62	Ledol
77	27.084	21628825	1.35	7202155	2.80	3.00	3-Oxatricyclo[4.1.1.0(2,4)]octane, 2,7,7-trimethyl-
78	27.212	203507	0.01	69722	0.03	2.92	
79	27.586	5976281	0.37	1646691	0.64	3.63	3-Cyclohexene-1-carboxaldehyde, 2,4,6-trimethyl-
80	27.687	1024261	0.06	339695	0.13	3.02	Tetracyclo[6.3.2.0(2,5).0(1,8)]tridecan-9-ol, 4,4-dimethyl-
81	28.100	5571065	0.35	1222129	0.47	4.56	1H-Cycloprop[e]azulen-4-ol, decahydro-1,1,4,7-tetramethyl-, [1ar-(1a.alpha.,4.bet
82	28.445	2164295	0.14	612409	0.24	3.53	Caryophyllene oxide
83	28.678	609791	0.04	182010	0.07	3.35	3,6-Octadien-1-ol, 3,7-dimethyl-, (Z)-
84	28.800	2278683	0.14	736827	0.29	3.09	
85	29.204	3371157	0.21	616936	0.24	5.46	.alpha.-Caryophyllene
86	29.808	157973	0.01	51807	0.02	3.05	
87	29.922	286274	0.02	67610	0.03	4.23	
88	30.088	176179	0.01	60933	0.02	2.89	
89	30.176	47348	0.00	20393	0.01	2.32	
90	30.653	6483642	0.41	2126014	0.83	3.05	Bicyclo[7.2.0]undec-4-ene, 4,11,11-trimethyl-8-methylene-
91	31.141	670864	0.04	267086	0.10	2.51	3.beta.-Acetoxy-5.alpha.-pregnan-20-one
92	31.226	10953122	0.69	3773665	1.47	2.90	.alpha.-Caryophyllene
93	31.307	106178	0.01	66784	0.03	1.59	1,5,9-Decatriene, 2,3,5,8-tetramethyl-
94	31.525	189322	0.01	75011	0.03	2.52	2,4-Heptadiene, 2,6-dimethyl-
95	32.029	1201196	0.08	316145	0.12	3.80	2-Pentadecanone, 6,10,14-trimethyl-
96	32.861	270731	0.02	102849	0.04	2.63	
97	33.040	4959246	0.31	1521319	0.59	3.26	Sclareoloxide
98	33.588	2319451	0.15	766813	0.30	3.02	Kaur-16-ene
99	33.766	204518	0.01	72962	0.03	2.80	cis-Z-.alpha.-Bisabolene epoxide

Figure S5: GC-MS total ion chromatogram (TIC) of the chemical profile from *Picea abies* obtained by SD extraction.

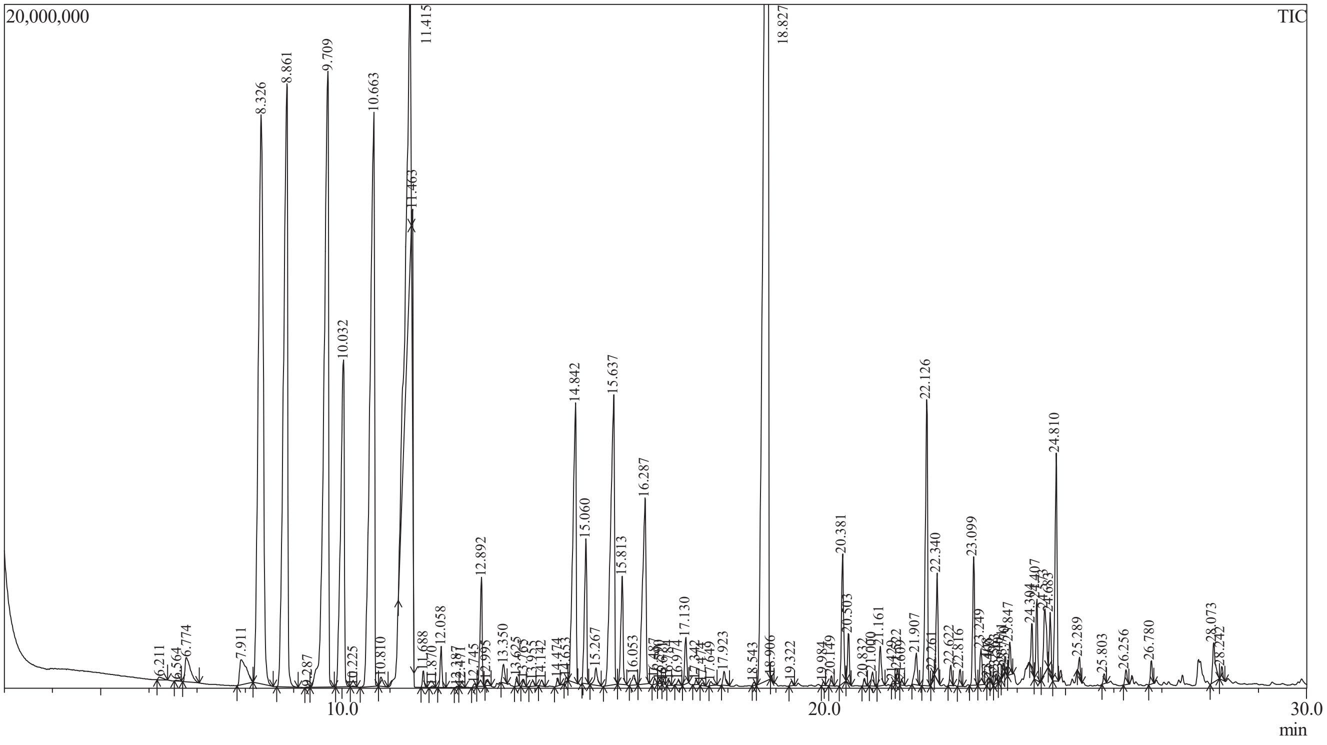

Peak Report TIC

Peak#	R.Time	Area	Area%	Height	Height%	A/H	Name
1	6.211	533731	0.05	80945	0.04	6.59	3-Hexen-1-ol, (E)-
2	6.564	110802	0.01	24945	0.01	4.44	1-Hexanol
3	6.774	5553084	0.49	712422	0.32	7.79	6-Propenylbicyclo[3.1.0]hexan-2-one
4	7.911	7160053	0.64	732660	0.33	9.77	Tricyclo[2.2.1.0(2,6)]heptane, 1,7,7-trimethyl-
5	8.326	118001086	10.49	16643180	7.59	7.09	.alpha.-Pinene
6	8.861	104788160	9.31	17641519	8.04	5.94	Camphene
7	9.287	55301	0.00	15986	0.01	3.46	
8	9.709	117840528	10.47	18007551	8.21	6.54	.beta.-Pinene
9	10.032	50814179	4.52	9562158	4.36	5.31	.beta.-Myrcene
10	10.225	75414	0.01	23945	0.01	3.15	
11	10.663	107411783	9.55	16791651	7.66	6.40	1,3,6-Octatriene, 3,7-dimethyl-, (E)-
12	10.810	1145306	0.10	266135	0.12	4.30	
13	11.415	60049711	5.34	8712465	3.97	6.89	Limonene
14	11.463	7558170	0.67	5975996	2.72	1.26	Eucalyptol
15	11.688	1207070	0.11	478499	0.22	2.52	(1S)-2,6,6-Trimethylbicyclo[3.1.1]hept-2-ene
16	11.870	157354	0.01	44008	0.02	3.58	2-Buten-1-ol, 3-methyl-, acetate
17	12.058	3076172	0.27	1205837	0.55	2.55	
18	12.387	87326	0.01	33054	0.02	2.64	
19	12.471	95548	0.01	32673	0.01	2.92	Artemiseole

Peak#	R.Time	Area	Area%	Height	Height%	A/H	Name
20	12.745	295512	0.03	95606	0.04	3.09	Cyclohexene, 1-methyl-4-(1-methylethylidene)-
21	12.892	9754280	0.87	3192858	1.46	3.06	Cyclohexene, 4-methyl-3-(1-methylethylidene)-
22	12.995	636501	0.06	156499	0.07	4.07	Bicyclo[2.2.1]heptan-2-one, 1,3,3-trimethyl-
23	13.350	1917007	0.17	538650	0.25	3.56	1,6-Octadien-3-ol, 3,7-dimethyl-
24	13.625	707148	0.06	273876	0.12	2.58	4-Penten-1-ol, propanoate
25	13.765	602671	0.05	202244	0.09	2.98	Valeric acid, 4-pentenyl ester
26	13.955	666438	0.06	158800	0.07	4.20	Bicyclo[2.2.1]heptan-2-ol, 1,3,3-trimethyl-, (1R-endo)-
27	14.142	599923	0.05	177739	0.08	3.38	Bicyclo[2.2.1]heptane-2,5-diol, 1,7,7-trimethyl-, (2-endo,5-exo)-
28	14.474	576166	0.05	224753	0.10	2.56	
29	14.653	331559	0.03	119948	0.05	2.76	Bicyclo[3.1.1]heptan-3-ol, 6,6-dimethyl-2-methylene-, [1S-(1.alpha.,3.alpha.,5.alp
30	14.842	38248904	3.40	8187616	3.73	4.67	Bicyclo[2.2.1]heptan-2-one, 1,7,7-trimethyl-, (1S)-
31	15.060	14102658	1.25	4233697	1.93	3.33	Bicyclo[2.2.1]heptan-2-ol, 2,3,3-trimethyl-
32	15.267	2175562	0.19	511557	0.23	4.25	Isoborneol
33	15.637	48065833	4.27	8483777	3.87	5.67	Borneol
34	15.813	10333799	0.92	3168522	1.44	3.26	3-Cyclohexen-1-ol, 4-methyl-1-(1-methylethyl)-
35	16.053	1388061	0.12	295278	0.13	4.70	Benzenemethanol, .alpha.,.alpha.,4-trimethyl-
36	16.287	27204515	2.42	5445082	2.48	5.00	p-menth-1-en-8-ol
37	16.487	529498	0.05	174542	0.08	3.03	4,7-Methanobenzofuran, 2,2'-oxybis[octahydro-7,8,8-trimethyl-, [2R[2.alpha.(2'R
38	16.590	241212	0.02	100382	0.05	2.40	Bicyclo[3.1.1]hept-3-en-2-one, 4,6,6-trimethyl-
39	16.671	151609	0.01	64509	0.03	2.35	2,4-Cycloheptadien-1-one, 2,6,6-trimethyl-
40	16.784	381083	0.03	157077	0.07	2.43	Bicyclo[2.2.1]heptan-2-ol, 1,3,3-trimethyl-, acetate, (1S-exo)-
41	16.974	516437	0.05	142250	0.06	3.63	Bicyclo[3.1.1]hept-3-en-2-ol, 4,6,6-trimethyl-
42	17.130	4217598	0.37	1364558	0.62	3.09	6-Octen-1-ol, 3,7-dimethyl-
43	17.342	350382	0.03	132423	0.06	2.65	Bicyclo[2.2.1]heptane-3-methylene-2,2-dimethyl-5-ol acetate
44	17.474	265746	0.02	86637	0.04	3.07	2-Cyclohexen-1-ol, 1-methyl-4-(1-methylethenyl)-, trans-
45	17.649	526014	0.05	124162	0.06	4.24	2-Cyclohexen-1-one, 2-methyl-5-(1-methylethenyl)-
46	17.923	1462877	0.13	403885	0.18	3.62	1-Acetoxy-p-menth-3-one
47	18.543	238841	0.02	92122	0.04	2.59	Acetic acid, undec-2-enyl ester
48	18.827	222678664	19.79	33514277	15.28	6.64	Bornyl acetate
49	18.906	232527	0.02	140767	0.06	1.65	2-Undecanone
50	19.322	442240	0.04	132761	0.06	3.33	
51	19.984	284205	0.03	112976	0.05	2.52	Santolina triene
52	20.149	818934	0.07	299858	0.14	2.73	2-Oxabicyclo[2.2.2]octan-6-ol, 1,3,3-trimethyl-, acetate
53	20.381	11620344	1.03	3786043	1.73	3.07	3-Cyclohexene-1-methanol, .alpha.,.alpha.,4-trimethyl-, acetate
54	20.503	3858924	0.34	1452551	0.66	2.66	Tricyclo[5.4.0.0(2,8)]undec-9-ene, 2,6,6,9-tetramethyl-
55	20.832	617877	0.05	221235	0.10	2.79	2-Dodecanone
56	21.000	1259762	0.11	406692	0.19	3.10	Ylangene
57	21.161	3772088	0.34	1150336	0.52	3.28	1H-Benzocycloheptene, 2,4a,5,6,7,8,9,9a-octahydro-3,5,5-trimethyl-9-methylene-
58	21.429	234992	0.02	85630	0.04	2.74	1,3-Cyclohexadiene, 5-(1,5-dimethyl-4-hexenyl)-2-methyl-, [S-(R@,S@)]-
59	21.522	1059568	0.09	422358	0.19	2.51	Cyclohexane, 1-ethenyl-1-methyl-2,4-bis(1-methylethenyl)-
60	21.609	368819	0.03	139756	0.06	2.64	1,4-Methano-1H-indene, octahydro-4-methyl-8-methylene-7-(1-methylethyl)-, [1S
61	21.907	3417556	0.30	926315	0.42	3.69	1H-Cycloprop[e]azulene, 1a,2,3,4,4a,5,6,7b-octahydro-1,1,4,7-tetramethyl-, [1aR-
62	22.126	27879761	2.48	8367227	3.81	3.33	1,4-Methanoazulene, decahydro-4,8,8-trimethyl-9-methylene-, [1S-(1.alpha.,3a.be
63	22.261	459618	0.04	198281	0.09	2.32	1H-3a,7-Methanoazulene, 2,3,4,7,8,8a-hexahydro-3,6,8,8-tetramethyl-, [3R-(3.alp
64	22.340	8208910	0.73	3123234	1.42	2.63	Bicyclo[7.2.0]undec-4-ene, 4,11,11-trimethyl-8-methylene-,[1R-(1R@,4Z,9S@)]-
65	22.622	1612245	0.14	595292	0.27	2.71	trans-.alpha.-Bergamotene
66	22.816	1368519	0.12	498359	0.23	2.75	Cyclohexene, 3-(1,5-dimethyl-4-hexenyl)-6-methylene-, [S-(R@,S@)]-
67	23.099	10536849	0.94	3755925	1.71	2.81	1,6,10-Dodecatriene, 7,11-dimethyl-3-methylene-
68	23.249	3185127	0.28	1050092	0.48	3.03	.alpha.-Caryophyllene
69	23.408	300615	0.03	132097	0.06	2.28	1H-Cyclopenta[1,3]cyclopropa[1,2]benzene, octahydro-7-methyl-3-methylene-4-(
70	23.460	170365	0.02	94971	0.04	1.79	Butanoic acid, 1,7,7-trimethylbicyclo[2.2.1]hept-2-yl ester, endo-
71	23.563	723655	0.06	278606	0.13	2.60	Cyclododeca-5,9-dien-1-ol, 2-methyl-, (Z,Z)-
72	23.640	188663	0.02	92325	0.04	2.04	Naphthalene, 1,2,3,5,6,8a-hexahydro-4,7-dimethyl-1-(1-methylethyl)-, (1S-cis)-
73	23.711	813073	0.07	348331	0.16	2.33	Naphthalene, 1,2,4a,5,6,8a-hexahydro-4,7-dimethyl-1-(1-methylethyl)-
74	23.770	338332	0.03	192191	0.09	1.76	Cyclopentanol, 1,2-dimethyl-3-(1-methylethenyl)-, [1R-(1.alpha.,2.alpha.,3.alpha.)
75	23.847	2523167	0.22	952551	0.43	2.65	Benzene, 1-(1,5-dimethyl-4-hexenyl)-4-methyl-
76	24.304	3041292	0.27	1344294	0.61	2.26	.alpha.-Muurolene
77	24.407	6158026	0.55	2376301	1.08	2.59	.alpha.-Farnesene
78	24.573	7763256	0.69	1879957	0.86	4.13	trans-.alpha.-Bergamotene
79	24.683	4260990	0.38	1782808	0.81	2.39	Naphthalene, 1,2,4a,5,6,8a-hexahydro-4,7-dimethyl-1-(1-methylethyl)-
80	24.810	19666299	1.75	6668250	3.04	2.95	Naphthalene, 1,2,3,5,6,8a-hexahydro-4,7-dimethyl-1-(1-methylethyl)-, (1S-cis)-
81	25.289	1109143	0.10	524799	0.24	2.11	.alpha.-Caryophyllene
82	25.803	788107	0.07	282472	0.13	2.79	1,6,10-Dodecatrien-3-ol, 3,7,11-trimethyl-, (E)-
83	26.256	1283616	0.11	432591	0.20	2.97	(-)-Spathulenol
84	26.780	1857105	0.17	680298	0.31	2.73	Bicyclo[3.3.1]non-2-en-9-ol, 9-methyl-
85	28.073	4724829	0.42	1155349	0.53	4.09	.alpha.-Cadinol
86	28.242	1037476	0.09	386850	0.18	2.68	
87	33.729	8147097	0.72	2714476	1.24	3.00	Cycloheptane, 4-methylene-1-methyl-2-(2-methyl-1-propen-1-yl)-1-vinyl-
88	34.020	1870578	0.17	596934	0.27	3.13	
89	34.344	674796	0.06	239787	0.11	2.81	
90	36.299	1748885	0.16	519491	0.24	3.37	Thunbergol
		1125315506	100.00	219350372			

Figure S6: GC-MS total ion chromatogram (TIC) of the chemical profile from *Salvia officinalis* obtained by SD extraction.

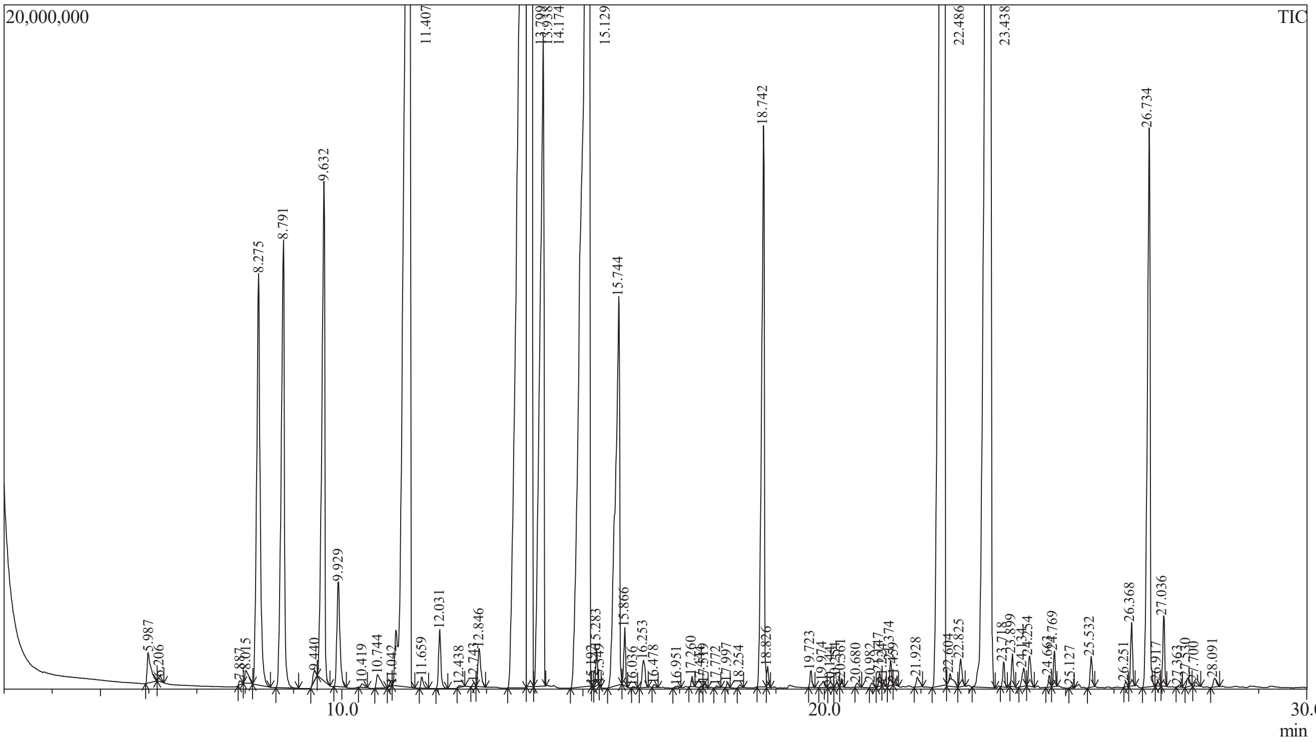

Peak Report TIC

Peak#	R.Time	Area	Area%	Height	Height%	A/H	Name
1	5.987	4619474	0.18	888174	0.24	5.20	2,5-Octadiene
2	6.206	364310	0.01	101424	0.03	3.59	1,6-Octadiene, 3,7-dimethyl-
3	7.887	402012	0.02	115036	0.03	3.49	Bicyclo[3.1.1]hept-2-ene, 2,6,6-trimethyl-, (.+/-.)-
4	8.015	2011473	0.08	365583	0.10	5.50	Bicyclo[3.1.0]hex-2-ene, 2-methyl-5-(1-methylethyl)-
5	8.275	60008696	2.39	12028231	3.25	4.99	.alpha.-Pinene
6	8.791	59840533	2.38	13088880	3.53	4.57	Camphene
7	9.440	1282990	0.05	269902	0.07	4.75	Bicyclo[3.1.0]hexane, 4-methylene-1-(1-methylethyl)-
8	9.632	63407367	2.53	14606759	3.94	4.34	Bicyclo[3.1.1]heptane, 6,6-dimethyl-2-methylene-, (1S)-
9	9.929	13605855	0.54	3068389	0.83	4.43	.beta.-Myrcene
10	10.419	509174	0.02	97796	0.03	5.21	.alpha.-Phellandrene
11	10.744	2466851	0.10	390219	0.11	6.32	Cyclohexene, 4-methyl-3-(1-methylethylidene)-
12	11.042	137739	0.01	0	0.00	0.00	
13	11.407	286261795	11.41	43675441	11.78	6.55	Eucalyptol
14	11.659	1207731	0.05	334630	0.09	3.61	1,3,7-Octatriene, 3,7-dimethyl-
15	12.031	5820409	0.23	1724537	0.47	3.38	1,4-Cyclohexadiene, 1-methyl-4-(1-methylethyl)-
16	12.438	255417	0.01	50586	0.01	5.05	Bicyclo[3.1.0]hexan-3-ol, 4-methyl-1-(1-methylethyl)-
17	12.743	335161	0.01	109793	0.03	3.05	Terpineol, cis-.beta.-
18	12.846	4655223	0.19	1107877	0.30	4.20	Cyclohexene, 1-methyl-4-(1-methylethylidene)-
19	13.799	372609098	14.85	38825974	10.48	9.60	1,6-Octadien-3-ol, 3,7-dimethyl-

Peak#	R.Time	Area	Area%	Height	Height%	A/H	Name
20	13.938	285713807	11.38	42519355	11.47	6.72	Bicyclo[3.1.0]hexan-3-one, 4-methyl-1-(1-methylethyl)-
21	14.174	109614738	4.37	19025704	5.13	5.76	Thujone
22	15.129	297548087	11.85	24901769	6.72	11.95	Bicyclo[2.2.1]heptan-2-one, 1,7,7-trimethyl-, (1S)-
23	15.192	53721	0.00	46500	0.01	1.16	Bicyclo[2.2.1]heptan-2-ol, 2,3,3-trimethyl-
24	15.283	2594939	0.10	1090851	0.29	2.38	1,6-Octadiene, 5,7-dimethyl-, (R)-
25	15.349	49024	0.00	40405	0.01	1.21	Isoborneol
26	15.744	69241546	2.76	11344542	3.06	6.10	Borneol
27	15.866	4210569	0.17	1696392	0.46	2.48	3-Cyclohexen-1-ol, 4-methyl-1-(1-methylethyl)-
28	16.036	105564	0.00	28966	0.01	3.64	Benzenemethanol, .alpha.,.alpha.,4-trimethyl-
29	16.253	3425724	0.14	796129	0.21	4.30	p-menth-1-en-8-ol
30	16.478	294561	0.01	77689	0.02	3.79	Bicyclo[3.1.0]hexan-3-ol, 4-methyl-1-(1-methylethyl)-
31	16.951	261630	0.01	52541	0.01	4.98	
32	17.260	798674	0.03	301046	0.08	2.65	Pentanoic acid, 5-hexen-1-yl ester
33	17.444	76278	0.00	35177	0.01	2.17	Hexyl n-valerate
34	17.519	276343	0.01	112096	0.03	2.47	2-Pentene, 1-ethoxy-4-methyl-, (Z)-
35	17.772	201453	0.01	52152	0.01	3.86	
36	17.997	420387	0.02	153863	0.04	2.73	
37	18.254	289273	0.01	81272	0.02	3.56	trans-2-Caren-4-ol
38	18.742	65097679	2.59	16403400	4.43	3.97	Bornyl acetate
39	18.826	1484911	0.06	615724	0.17	2.41	6,8-Nonadien-2-one, 8-methyl-5-(1-methylethyl)-, (E)-
40	19.723	1395779	0.06	484343	0.13	2.88	(-)-Myrtenyl acetate
41	19.974	800591	0.03	175338	0.05	4.57	
42	20.144	100376	0.00	30314	0.01	3.31	2-Oxabicyclo[2.2.2]octan-6-ol, 1,3,3-trimethyl-, acetate
43	20.254	429806	0.02	141841	0.04	3.03	(-)-trans-Pinocarvyl acetate
44	20.361	609807	0.02	237802	0.06	2.56	.alpha.-Cubebene
45	20.680	448970	0.02	119269	0.03	3.76	Myrcenylacetat
46	20.982	545704	0.02	128912	0.03	4.23	Ylangene
47	21.147	1001433	0.04	389253	0.11	2.57	Copaene
48	21.224	176357	0.01	69949	0.02	2.52	2,6-Octadien-1-ol, 3,7-dimethyl-, acetate, (E)-
49	21.374	2005546	0.08	714206	0.19	2.81	.alpha.-Bourbonene
50	21.459	198724	0.01	77705	0.02	2.56	
51	21.928	1332572	0.05	282918	0.08	4.71	Bicyclo[7.2.0]undec-4-ene, 4,11,11-trimethyl-8-methylene-,[1R-(1R@,4Z,9S@)]-
52	22.486	318069914	12.67	43680663	11.79	7.28	Caryophyllene
53	22.604	2235145	0.09	364709	0.10	6.13	1,6-Cyclodecadiene, 1-methyl-5-methylene-8-(1-methylethyl)-, [s-(E,E)]-
54	22.825	2893600	0.12	806225	0.22	3.59	Aromadendrene
55	23.438	332666876	13.25	40185979	10.84	8.28	.alpha.-Caryophyllene
56	23.718	2021992	0.08	731021	0.20	2.77	Naphthalene, 1,2,4a,5,6,8a-hexahydro-4,7-dimethyl-1-(1-methylethyl)-
57	23.899	2507337	0.10	953516	0.26	2.63	1,6-Cyclodecadiene, 1-methyl-5-methylene-8-(1-methylethyl)-, [s-(E,E)]-
58	24.134	2094733	0.08	536716	0.14	3.90	1H-Cycloprop[e]azulene, 1a,2,3,5,6,7,7a,7b-octahydro-1,1,4,7-tetramethyl-, [1aR
59	24.254	3120822	0.12	884398	0.24	3.53	.gamma.-Elemene
60	24.663	907080	0.04	339969	0.09	2.67	Naphthalene, 1,2,4a,5,6,8a-hexahydro-4,7-dimethyl-1-(1-methylethyl)-
61	24.769	2769668	0.11	1039639	0.28	2.66	Naphthalene, 1,2,3,5,6,8a-hexahydro-4,7-dimethyl-1-(1-methylethyl)-, (1S-cis)-
62	25.127	189325	0.01	68285	0.02	2.77	Naphthalene, 1,2,3,4,4a,7-hexahydro-1,6-dimethyl-4-(1-methylethyl)-
63	25.532	2721744	0.11	894860	0.24	3.04	3,7-Cyclodecadiene-1-methanol, .alpha.,.alpha.,4,8-tetramethyl-, [s-(Z,Z)]
64	26.251	413092	0.02	149890	0.04	2.76	(-)-Spathulenol
65	26.368	5251223	0.21	1857958	0.50	2.83	Caryophyllene oxide
66	26.734	66282727	2.64	16338443	4.41	4.06	Epiglobulol
67	26.917	366679	0.01	126948	0.03	2.89	Ledol
68	27.036	5678694	0.23	2057455	0.56	2.76	3-Oxatricyclo[4.1.1.0(2,4)]octane, 2,7,7-trimethyl-
69	27.363	236476	0.01	65612	0.02	3.60	
70	27.530	978611	0.04	257908	0.07	3.79	
71	27.700	473377	0.02	126575	0.03	3.74	
72	28.091	1151977	0.05	258421	0.07	4.46	2-Naphthalenemethanol, decahydro-.alpha.,.alpha.,4a-trimethyl-8-methylene-, [2R
73	30.635	1975519	0.08	642571	0.17	3.07	
74	31.215	2264642	0.09	634351	0.17	3.57	
75	33.570	2588751	0.10	785783	0.21	3.29	Kaur-16-ene
76	33.986	521473	0.02	174750	0.05	2.98	
77	36.279	18911444	0.75	3584005	0.97	5.28	
		2509898802	100.00	370623304			

Table S1. Chemical composition of *Salvia officinalis* SEO and HEO.

RT (min)	Compound	Steam distillation	Hydro-distillation	MAE*	RI^{exp}	RI^{lit}	Organoleptic properties
**8.031**	α-thujene	0.085±0.0071	0.065±0.0071		938	932	
**8.260**	α-pinene	2.555±0.3182	1.53±0.1697		945	942	sharp warm resinous fresh pine
**8.757**	camphene	2.555±0.3182	1.59±0.1697		961	960	woody herbal fir needle camphor terpenic
**9.428**	sabinene	0.055±0.0071	0.05±0.0000		980	980	
**9.581**	β-pinene	2.725±0.3041	1.22±0.1273		985	985	
**9.927**	β-myrcene	0.59±0.0566	0.74±0.0707		994	994	
**10.763**	α-terpinen	0.105±0.0071	0.15±0.0141		1013	1013	
**11.137**	d-limonene	0.125±0.0212	0.18±0.0424		1020	1020	
**11.339**	eucalyptol	11.535±1.3647	6.65±0.5233	24.32	1024	1024	eucalyptus herbal camphoreous medicinal
**11.676**	β-ocimene	0.055±0.0071	0.03±0.0000		1030	1030	
**12.033**	γ-terpinen	0.25±0.0283	0.335±0.0354	0.32	1037	1035	
**12.608**	cis-β-terpineol	0.02±0.0000	0.045±0.0071		1047	1045	
**12.858**	α-terpinolen	0.21±0.0141	0.245±0.0212		1051	1051	
**13.665**	β-linalool	7.555±8.5065	1.205±0.3889	1.05	1064	1062	citrus floral sweet bois de rose woody green blueberry
**13.810**	thujone	12.955±0.4313	13.59±5.3174	3.05	1067	1066	cedar thujonic
**14.065**	β-thujone	4.735±0.5020	5.635±0.3748	21.01	1070	1070	cedar thujonic spicy woody
**15.021**	camphor	12.935±1.2233	14.615±0.8697	40.16	1084	1085	camphoreous
**15.220**	isopinocamphone		0.12±0.0141		1087	1087	
**15.283**	isocitronellene	0.115±0.0071			1088	1088	
**15.646**	borneol	2.99±0.3111	2.94±0.1697	7.39	1093	1091	pine woody camphoreous peppery
**15.809**	terpinen-4-ol	0.185±0.0212	0.295±0.0212	0.7	1095	1096	
**16.236**	α-terpineol	0.15±0.0141	0.35±0.0283	0.25	1201	1204	
**16.476**	isothujol	0.01±0.0000	0.04±0.0000	0.17	1206	1206	
**17.666**	linalyl acetate		0.26±0.0283		1230	1230	
**18.709**	bornyl acetate	2.8±0.3111	1.9±0.1131		1250	1253	sweet balsamic woody fresh fir needle herbal
**18.836**	solanone	0.065±0.0071	0.02±0.0000		1252	1253	
**19.749**	myrtenyl acetate	0.06±0.0000	0.1±0.0000		1268	1271	
**20.698**	neryl acetate		0.085±0.0071		1284	1282	
**21.165**	copaene	0.045±0.0071	0.075±0.0071		1291	1292	
**21.390**	β-bourbonene	0.085±0.0071	0.095±0.0212		1295	1297	
**22.421**	β-caryophyllene	13.79±1.3718	7.38±0.4950		1416	1415	sweet woody spicy clove dry
**22.599**	β-cubebene		0.035±0.0071		1420	1419	
**22.827**	aromadendrene	0.125±0.0071	0.115±0.0071		1425	1420	

**23.364**	α-humulene	14.5±1.3152	8.88±0.5091		1436	1435	sweet woody spicy clove dry
**23.718**	α-amorphene	0.085±0.0071			1443	1440	
**23.898**	germacrene D	0.11±0.0141	0.13±0.0141		1447	1446	
**24.140**	viridiflorene	0.095±0.0071	0.165±0.0071		1452	1453	
**24.254**	γ-elemene	0.135±0.0071			1454	1454	
**24.780**	δ-cadinene	0.12±0.0141	0.19±0.0141		1465	1464	
**25.146**	cadina-1,4-diene	0.01±0.0000	0.015±0.0071		1472	1473	
**25.532**	hedycaryol	0.125±0.0071			1479	1479	
**26.284**	(-)-spathulenol	0.02±0.0000	0.105±0.0071		1493	1493	
**26.392**	caryophyllene oxide	0.225±0.0212	1.07±0.0707		1495	1495	
**26.820**	viridiflorol	2.885±0.2758	11.13±0.5515		1604	1607	sweet green herbal fruity tropical minty
**26.963**	ledol	0.015±0.0071	0.07±0.0000		1608	1608	
**27.084**	α-pinene epoxide	0.245±0.0212	1.525±0.0919		1611	1612	
**27.530**	α-selinene	0.045±0.0071			1621	1623	
**27.586**	isocyclocitral 1		0.42±0.0283		1623	1623	
**32.029**	hexahydrofarnesyl acetone		0.08±0.0141		1824	1827	
**33.040**	sclareoloxide		0.355±0.0071		1850	1867	
**33.588**	kaur-16-ene	0.105±0.0212	0.17±0.0000		1863	1867	
**36.357**	epimanool	0.825±0.0778	8.66±0.1131		2034	2032	fresh, herbal, and woody, coniferous undertones with a subtle sweet or floral nuance
**36.859**	humulane-1,6-dien-3-ol	0.015±0.0071	0.62±0.0000		2048	2047	
	**Total**	99.03	95.27	98.42			
	**Total Monoterpene Hydrocarbon**	9.425	6.135	0.32			
	**Total Sesquiterpene Hydrocarbon**	29.145	17.08	0			
	**Total Oxygenated Monoterpene Oxide**	11.78	8.175	24.32			
	**Total Oxygenated Monoterpene Alcohol**	10.91	4.875	9.56			
	**Total Oxygenated Monoterpene Ketone**	30.625	33.96	64.22			
	**Total Oxygenated Monoterpene Aldehyde**	0	0.42	0			
	**Total Oxygenated Monoterpene Ester**	2.86	2.345	0			
	**Total Oxygenated Sesquiterpene Alcohol**	3.06	11.925	0			
	**Total Oxygenated Sesquiterpene Oxide**	0.225	1.07	0			

**Total Diterpenes and Derivatives**	0.93	9.185	0
**Other compounds**	0.065	0.1	0

Table S2. Chemical composition of *Mentha piperita* SEO and HEO.

RT (min)	Compound	Steam distillation	Hydro-distillation	MAE*	RI^{exp}	RI^{lit}	Organoleptic properties
**8.008**	α-thujene	0.01±0.0000	0.185±0.0778		937	932	
**8.216**	α-pinene	0.215±0.0495	0.475±0.2051		944	942	
**9.397**	sabinene	0.12±0.0424	0.02±0.0141		980	980	
**9.540**	β-pinene	0.335±0.1061	0.82±0.2404		983	983	
**9.896**	β-myrcene	0.285±0.0778	0.61±0.1414		993	992	
**10.218**	octan-3-ol	0.15±0.0283	0.445±0.0919	0.64	1001	1001	
**10.996**	p-cymene	0.05±0.0141	0.115±0.0354		1017	1017	
**11.116**	d-limonene	0.225±0.1061	0.53±0.1980		1020	1019	
**11.266**	eucalyptol	3.455±0.9829	3.575±0.5303	8.15	1023	1024	eucalyptus herbal camphor medicinal
**11.640**	β-ocimene	0.05±0.0141	0.07±0.0283		1030	1030	
**11.999**	γ-terpinen	0.08±0.0141	0.16±0.0283	0.14	1036	1035	
**12.428**	cis-sabinene hydrate	0.115±0.0212	0.865±0.1202		1044	1043	
**12.831**	α-terpinolen	0.035±0.0071	0.035±0.0071	0.14	1051	1050	
**13.351**	3-methylbutyl 2-methylbutanoate;	0.085±0.0212	0.175±0.0212		1059	1052	
**13.519**	β-linalool	0.275±0.0919	0.205±0.0212	0.54	1062	1062	
**13.862**	octan-3-yl acetate	0.045±0.0071	0.14±0.0141		1067	1068	
**14.861**	sabinol		0.085±0.0071	0.06	1082	1083	
**15.419**	menthone	59.835±2.1567	41.975±2.6799	35.91	1090	1089	mentholic peppermint herbal camphoreous
**15.478**	menthofuran	0.435±0.0071			1091	1090	
**15.547**	isomenthone	2.035±0.0495	4.23±0.2970	4.79	1092	1091	minty
**15.629**	p-menthan-1-ol	1.655±0.0919			1093	1091	
**15.732**	isopulegone	0.085±0.0071			1094	1096	
**15.780**	isomentol		2.56±0.2263	1.48	1095	1096	mentholic musty woody camphoreous
**16.046**	menthol	17.11±0.2828	21.38±0.6788	36.2	1098	1099	peppermint cooling mentholic minty
**16.332**	α-terpineol	0.075±0.0071	0.12±0.0141	0.23	1203	1204	
**17.341**	hex-5-en-1-yl pentanoate	0.055±0.0071	0.14±0.0000		1224	1219	
**17.587**	pulegone	5.495±0.0778	11.025±0.0495	8.09	1229	1229	minty, sulfuraceous, sweet with metallic buchu nuances
**17.669**	carvone	0.37±0.0141		0.13	1230	1230	
**17.957**	piperitone	0.675±0.0071	1.635±0.1202		1236	1236	
**18.336**	neomenthyl acetate		0.08±0.0000		1243	1243	
**18.831**	menthyl acetate	0.895±0.0212	1.375±0.0212		1252	1253	
**20.275**	verbenone	0.025±0.0071	0.09±0.0000		1277	1277	
**21.368**	α-bourbonene	0.125±0.0071	0.185±0.0071		1295	1297	
**21.499**	β-elemene	0.28±0.0283	0.045±0.0071		1297	1297	
**22.056**	exo-2-hydroxycineole		0.065±0.0071		1408	1408	
**22.325**	β-caryophyllene	1.025±0.0495	0.855±0.0778		1414	1415	
**23.075**	β-farnesene	0.095±0.0071	0.17±0.0141		1430	1420	
**23.224**	α-humulene	0.04±0.0000	0.045±0.0071		1433	1435	
**23.386**	β-cubebene	0.06±0.0000			1436	1437	
**23.691**	α-amorphene	0.05±0.0000	0.14±0.0000		1443	1440	

**23.874**	germacrene D	0.435±0.0495			1447	1446
**24.083**	eudesma-4(14),11-diene	0.065±0.0071	0.265±0.0354		1451	1453
**24.230**	γ-elemene	0.1±0.0283			1454	1454
**24.263**	α-selinene		0.22±0.0141		1454	1454
**24.397**	mint furanone	0.28±0.0283	1.51±0.1556		1457	1458
**24.752**	α-cubebene	0.035±0.0071	0.065±0.0071		1464	1464
**26.235**	(-)-spathulenol	0.045±0.0071	0.065±0.0071		1492	1493
**26.355**	caryophyllene oxide	0.305±0.0071	0.52±0.0990		1495	1495
**26.659**	ledol	0.085±0.0071			1600	1607
**26.678**	epiglobulol		0.195±0.0354		1601	1607
**28.055**	α-cadinol	0.01±0.0000	0.06±0.0141		1633	1633
	**Total**	97.31	97.53	96.5		
	**Total Monoterpene Hydrocarbon**	1.405	3.02	0.28		
	**Total Sesquiterpene Hydrocarbon**	2.31	1.99	0		
	**Total Oxygenated Monoterpene Oxide**	3.89	3.575	8.15		
	**Total Oxygenated Monoterpene Alcohol**	19.23	25.28	38.51		
	**Total Oxygenated Monoterpene Ketone**	68.52	58.955	48.92		
	**Total Oxygenated Monoterpene Ester**	0.895	1.455	0		
	**Total Oxygenated Sesquiterpene Alcohol**	0.14	0.32	0		
	**Total Oxygenated Sesquiterpene Oxide**	0.305	0.52	0		
	**Other compounds**	0.615	2.41	0.64		

Table S3. Chemical composition of *Achillea millefolium* SEO and HEO.

RT (min)	Compound	Steam distillation	Hydro-distillation	RI^{exp}	RI^{lit}	Organoleptic properties
8.015	α-thujene	0.105±0.0071		937	932	
8.239	α-pinene	0.62±0.0283	0.805±0.0495	945	942	
8.713	camphene	0.04±0.0000	0.035±0.0071	960	960	
9.665	sabinene	15.615±0.9970	5.91±0.1838	987	986	woody terpene citrus pine spice camphoreous nuances
9.787	β-pinene	4.995±0.1909	4.895±0.1768	990	987	dry woody resinous pine hay green eucalyptus camphoreous
9.948	β-myrcene	0.185±0.0071	0.325±0.0354	994	994	
10.114	2,6-dimethyl-3,7-octadien-2-ol	0.04±0.0000	0.185±0.0212	999	997	
10.762	γ-terpinen	0.15±0.0000		1013	1013	
11.010	p-cymene	0.06±0.0000	0.095±0.0071	1018	1017	
11.200	d-limonene	0.055±0.0212	0.11±0.0141	1021	1021	
11.230	sulcatone		2.09±0.0849	1022	1021	
11.288	eucalyptol	0.995±0.0354		1023	1024	
11.306	α-ocimene	0.14±0.0000	0.045±0.0071	1030	1030	
11.629	β-ocimene		0.35±0.0283	1039	1039	
12.047	2,4-dimethyl-octa-2,6-diene	3.94±0.0141	4.275±0.1768	1037	1039	
12.409	cis-β-terpineol		0.325±0.0212	1044	1043	
12.694	artemisia alcohol	0.06±0.0000	0.21±0.0141	1049	1050	
12.820	α-terpinolen		0.535±0.0495	1051	1050	
13.310	β-linalool	0.04±0.0000	2.735±0.1061	1059	1052	
13.481	octen-1-ol acetate		0.105±0.0071	1061	1061	
14.062	cis-2-p-menthen-1-ol;		0.15±0.0141	1070	1070	
14.561	trans-pinocarveol	0.04±0.0000	0.12±0.0000	1078	1075	
14.736	camphor	0.13±0.0000	0.555±0.0354	1080	1083	
15.005	myrtanol		0.35±0.0141	1084	1085	
15.172	pinocarvone		0.095±0.0071	1086	1087	
15.241	lavandulol		0.365±0.0071	1087	1087	
15.516	borneol	0.05±0.0000	0.48±0.0141	1091	1091	
15.805	terpinen-4-ol	1.8±0.0424	6.19±0.0141	1095	1096	pepper woody earth musty sweet
15.988	n-hexyl butanoate		0.095±0.0071	1097	1097	
16.183	α-terpineol	0.22±0.0000	1.705±0.0354	1100	1099	
18.234	trans-sabinyl acetate	0.575±0.0071		1241	1240	
17.268	4,4,5-trimethyl-2-hexene		0.165±0.0071	1222	1219	
17.639	linalyl acetate		2.345±0.0212	1230	1230	
18.397	isomyrcenyl acetate	0.215±0.0071	0.345±0.0071	1244	1244	
18.592	lavandulyl acetate	0.215±0.0071		1248	1245	
18.661	bornyl acetate	0.22±0.0000		1249	1245	

18.962	trans-pinocarvyl acetate	0.05±0.0000	0.695±0.0071	1254	1254	
20.037	δ-eIemene;	0.065±0.0071		1273	1274	
20.354	α-terpenyl acetate	0.15±0.0000	0.155±0.0071	1278	1277	
20.665	neryl acetate	0.04±0.0000	1.275±0.0212	1283	1282	
21.172	geranyl acetate		0.2±0.0141	1292	1292	
21.151	copaene	0.155±0.0071		1291	1292	
21.392	β-bourbonene	0.91±0.0424	0.535±0.0071	1295	1297	
21.712	1-tetradecen-3-yne		0.105±0.0071	1400	1399	
22.507	β-caryophyllene	22.895±0.2616	13.585±0.2475	1418	1419	sweet woody spicy clove dry
22.621	isogermacrene D	0.415±0.0212	0.28±0.0000	1420	1420	
22.833	β-sesquiphellandrene	0.205±0.0071	0.43±0.0141	1425	1420	
22.955	aromadendrene	0.105±0.0071	0.125±0.0071	1427	1420	
23.174	β-farnesene	0.7±0.0283	0.7±0.0141	1432	1430	
23.298	α-humulene	2.125±0.0919	2.46±0.0283	1435	1435	
24.130	germacrene D	21.755±0.2616	7.48±0.0849	1452	1453.0437	woody spice
24.239	zingiberene	0.705±0.0354	2.555±0.0071	1454	1454	
24.335	γ-elemene	0.49±0.0283		1456	1455	
24.421	α-farnesene	0.445±0.0354		1458	1458	
24.696	α-amorphene	0.055±0.0071	0.04±0.0000	1463	1464	
24.813	δ-cadinene	0.34±0.0141	0.63±0.0000	1465	1464	
25.521	elemol		0.07±0.0000	1479	1479	
25.817	nerolidol	0.395±0.0212	1.05±0.0283	1485	1485	
26.143	β-elemene		0.12±0.0000	1491	1491	
26.317	(-)-spathulenol	0.535±0.0354	0.83±0.0424	1641	1641	
26.455	caryophyllene oxide	3.21±0.1556	4.755±0.0636	1496	1495	sweet fresh dry woody spicy
26.673	trans-α-bisabolene epoxide	0.1±0.0000		1601	1607	
27.012	tridec-3-ene		0.57±0.0283	1609	1609	
27.508	guaiyl acetate	0.095±0.0071		1621	1619	
27.656	Grandlure II		0.2±0.0141	1624	1623	
27.727	τ-cadinol		0.05±0.0283	1626	1628	
28.077	β-eudesmol	0.49±0.0283		1634	1633	
28.717	limonene dioxide	0.24±0.0000	1.08±0.0566	1649	1646	
28.880	(+)-spathulenol		0.085±0.0071	1652	1651	
29.432	tridecanal	0.505±0.0354	0.225±0.0071	1664	1668	
29.951	chamazulene	9.45±0.1980	20.245±0.4879	1676	1675	strong, herbal, and slightly bitter, aromatic, or tea-like scent
	**Total**	97.13	96.52			
	**Total Monoterpene Hydrocarbon**	21.97	13.11			
	**Total Sesquiterpene Hydrocarbon**	60.82	49.19			
	**Total Oxygenated Monoterpene Alcohol**	2.25	13.02			

**Total Oxygenated Monoterpene Ketone**	0.13	0.65
**Total Oxygenated Monoterpene Ester**	1.47	5.02
**Total Oxygenated Monoterpene Ether**	1.24	1.08
**Total Oxygenated Sesquiterpene Alcohol**	1.42	2.09
**Total Oxygenated Sesquiterpene Ester**	0.10	0.00
**Total Oxygenated Sesquiterpene Oxide**	3.31	4.76
**Other compounds**	4.45	7.63

Table S4. Chemical composition of *Mentha spicata* SEO and HEO.

RT (min)	Compound	Steam distillation	Hydro-distillation	RI^{exp}	RI^{lit}	Organoleptic properties
8.244	α-pinene	0.475±0.0212	0.33±0.0283	945	942	
9.440	sabinene	0.365±0.0071	0.295±0.0212	981	980	
9.582	β-pinene	0.61±0.0141	0.505±0.0495	985	985	dry woody resinous pine hay green eucalyptus camphoreous
9.995	β-myrcene	3.675±0.0212	2.525±0.1344	996	997	Terpy, herbaceous, woody with a rosy celery and carrot nuance
10.762	α-terpinen		0.085±0.0071	1013	1013	
11.316	d-limonene	4.17±0.0424	3.61±0.1273	1024	1024	citrus orange fresh sweet sweet citrus peely
"						
11.380	eucalyptol	1.335±0.0778	1.435±0.0354	1025	1025	
11.673	β-ocimene	0.075±0.0071	0.06±0.0000	1030	1030	
12.041	γ-terpinen	0.32±0.0000	0.205±0.0071	1037	1039	
12.476	cis-β-terpineol	0.895±0.0071	1.865±0.0919	1045	1044	
12.856	α-terpinolen	0.27±0.0000	0.09±0.0000	1051	1051	
13.373	decen-2-al	0.255±0.0071		1060	1052	
13.382	β-linalool		0.165±0.0071	1060	1052	
14.105	cis-2-p-menthen-1-ol	0.045±0.0071	0.04±0.0000	1071	1072	
14.451	limonene oxide		0.12±0.0000	1076	1075	
15.023	menthone	0.1±0.0000	0.065±0.0212	1084	1085	
15.784	terpinen-4-ol	1.29±0.0283	1.215±0.0636	1095	1096	
16.295	dodec-3-yne		1.775±0.0778	1202	1204	
16.301	dihydrocarvone	0.9±0.0000		1202	1204	
17.298	cis-3-Hexenyl isovalerate	0.15±0.0000	0.205±0.0071	1223	1219	
17.501	cis-carveol		0.195±0.0071	1227	1229	
17.964	carvone	79.12±0.1838	74.96±0.8202	1242	1240	sweet spearmint herbal minty
18.340	piperitone	0.05±0.0141	0.19±0.0141	1243	1243	
18.414	trans-carvone oxide	0.095±0.0071	0.38±0.0141	1244	1244	
18.664	cis-carvone oxide	0.125±0.0071	0.565±0.0212	1249	1245	
18.818	bornyl acetate	0.02±0.0000	0.13±0.0141	1252	1253	
19.805	1-acetoxymethyl-3-isopropenyl-2-methylcyclopentane		0.1±0.0000	1269	1271	
20.723	cis-carvyl acetate	0.09±0.0000	0.16±0.0000	1284	1286	
21.183	cis-jasmone		0.115±0.0071	1292	1292	
21.441	β-bourbonene	0.93±0.0283	1.36±0.0000	1296	1297	
21.560	β-elemene	0.72±0.0000	1.18±0.0000	1298	1298	
22.378	β-caryophyllene	1.155±0.0071	1.705±0.0071	1415	1415	
22.641	trans-α-bergamotene	0.33±0.0000		1421	1420	
22.974	aromadendrene	0.135±0.0071	0.225±0.0071	1428	1420	
23.111	β-farnesene	0.13±0.0000	0.25±0.0000	1431	1430	
23.427	β-cubebene	0.1±0.0000	0.17±0.0000	1437	1437	
23.591	isogermacrene D	0.07±0.0000	0.125±0.0071	1441	1440	
23.916	germacrene D	0.65±0.0000	0.975±0.0071	1447	1446	
24.263	γ-elemene	0.19±0.0000	0.34±0.0000	1454	1454	
24.686	α-amorphene	0.05±0.0000	0.03±0.0000	1463	1462	

24.870	calamenene	0.02±0.0000	0.05±0.0000	1466	1464
26.270	(-)-spathulenol		0.12±0.0000	1493	1493
26.406	caryophyllene oxide	0.02±0.0000	0.155±0.0071	1496	1495
27.181	cubenol	0.02±0.0000	0.1±0.0000	1613	1612
28.108	α-cadinol		0.09±0.0000	1635	1633
	**Total**	98.95	98.26		
	**Total Monoterpene Hydrocarbon**	9.96	7.705		
	**Total Sesquiterpene Hydrocarbon**	4.48	6.41		
	**Total Oxygenated Monoterpene Oxide**	1.335	1.555		
	**Total Oxygenated Monoterpene Alcohol**	2.23	3.48		
	**Total Oxygenated Monoterpene Ketone**	80.39	76.16		
	**Total Oxygenated Monoterpene Ester**	0.11	0.29		
	**Total Oxygenated Sesquiterpene Alcohol**	0.02	0.31		
	**Total Oxygenated Sesquiterpene Oxide**	0.02	0.16		
	**Other compounds**	0.405	2.195		

Table S5. Chemical composition of *Hyssopus officinalis* SEO and HEO.

RT (min)	Compound	Steam distillation	Hydro-distillation	RI^{exp}	RI^{lit}	Organoleptic properties
8.021	α-thujene	0.105±0.0071	0.085±0.0071	937	932	
8.235	α-pinene	0.33±0.0000	0.235±0.0071	945	942	
8.721	camphene	0.07±0.0000	0.03±0.0000	960	960	
9.458	sabinene		0.395±0.0354	981	981	
9.743	β-pinene	12.715±0.1909	5.32±0.1556	989	987	dry woody resinous pine hay green eucalyptus camphoreous
9.992	β-myrcene	1.485±0.0071	1.555±0.0495	995	994	
10.772	α-terpinen	0.05±0.0000	0.055±0.0071	1013	1013	
11.338	β-phellandrene	7.115±0.0636	5.21±0.0283	1024	1024	mint terpentine
11.673	β-ocimene	0.3±0.0000	0.445±0.0071	1030	1030	
12.028	γ-terpinen	0.105±0.0071	0.135±0.0071	1037	1035	
12.446	cis-β-terpineol		0.41±0.0000	1044	1044	
12.848	α-terpinolen	0.055±0.0071	0.055±0.0071	1051	1051	
13.694	β-linalool	0.495±0.0071	1.34±0.0424	1065	1062	
13.887	thujone	0.07±0.0000	0.18±0.0000	1068	1068	
14.578	pinane		0.07±0.0000	1078	1075	
14.779	camphor		0.445±0.0495	1081	1083	
15.024	perillyl methyl ether	2.45±0.0000	2.81±0.0000	1084	1085	
15.310	D-pinocamphone	8.115±0.1202	8.12±0.1131	1088	1088	cedar camphoreous
16.154	isopinocamphone	39.575±0.0495	39.58±0.0424	1100	1099	terpineol like camphoreous
16.397	myrtenol	0.58±0.0000	1.26±0.0000	1204	1204	
17.662	nerol		0.195±0.0071	1230	1230	
18.673	bornyl acetate		0.075±0.0071	1249	1245	
18.962	thymol	0.535±0.0071		1254	1254	
19.748	myrtenyl acetate	0.09±0.0000	0.025±0.0071	1268	1271	
19.983	germacrene B	0.36±0.0000	0.365±0.0071	1272	1271	
20.666	neryl acetate	0.07±0.0000	0.075±0.0071	1283	1282	
21.166	copaene	0.08±0.0000	0.05±0.0000	1291	1292	
21.430	β-bourbonene	1.34±0.0283	0.885±0.0071	1296	1297	
21.497	β-elemene	0.1±0.0000	0.065±0.0071	1297	1297	
21.838	methyl eugenol	0.1±0.0000	0.065±0.0071	1403	1403	
22.017	α-gurjunene	0.575±0.0071	0.33±0.0000	1407	1408	
22.432	β-caryophyllene	5.1±0.0424	2.9±0.0424	1416	1415	sweet woody spicy clove dry
22.828	aromadendrene	2.265±0.0354	1.62±0.0141	1425	1420	
23.126	β-bisabolene		0.215±0.0071	1431	1430	
23.156	isocaryophyllene	0.185±0.0071	0.035±0.0071	1432	1430	
23.283	α-humulene	0.62±0.0000	0.825±0.0778	1434	1435	
24.027	germacrene D	6.22±0.0141	4.13±0.0283	1450	1453	woody spice
24.149	α-bulnesene	0.18±0.0000	0.205±0.0071	1452	1453	
24.381	γ-elemene	4.095±0.0495	3.91±0.0283	1457	1458	green woody oily
24.690	α-amorphene	0.105±0.0071	0.1±0.0000	1463	1462	
24.792	δ-cadinene	0.08±0.0000	0.045±0.0071	1465	1464	
25.614	hedycaryol	2.17±0.0000	0.12±0.0141	1481	1481	
25.671	elemol		10.4±0.0990	1482	1481	green woody spicy rose
26.275	(-)-spathulenol	0.215±0.0071	0.96±0.0141	1493	1493	
26.397	caryophyllene oxide	0.21±0.0000	0.57±0.0141	1495	1495	
26.494	globulol		0.105±0.0071	1497	1495	

26.700	viridiflorol		0.425±0.2333	1601	1607
26.937	ledol	0.13±0.0000	0.435±0.0071	1607	1607
27.340	α-eudesmol		0.085±0.0071	1617	1612
27.541	γ-eudesmol	0.3±0.0000	0.91±0.0000	1621	1623
27.750	τ-cadinol	0.09±0.0000	0.3±0.0000	1626	1628
28.093	β-eudesmol	0.44±0.0000	1.645±0.0071	1634	1633
	**Total**	99.27	99.81		
	**Total Monoterpene Hydrocarbon**	22.33	13.59		
	**Total Sesquiterpene Hydrocarbon**	21.305	15.68		
	**Total Oxygenated Monoterpene Alcohol**	1.075	3.205		
	**Total Oxygenated Monoterpene Ketone**	47.76	48.325		
	**Total Oxygenated Monoterpene Ester**	0.16	0.175		
	**Total Oxygenated Monoterpene Ether**	2.45	2.81		
	**Total Oxygenated Sesquiterpene Alcohol**	3.345	15.385		
	**Total Oxygenated Sesquiterpene Oxide**	0.21	0.57		
	**Total Phenylpropanoid**	0.1	0.065		

Unsupervised Principal Component Analysis (PCA) and Hierarchical Cluster Analysis (HCA) for *Mentha piperita*

	F1	F2	F3
Eigenvalue	44.955	4.771	1.274
Variability (%)	88.148	9.354	2.498
Cumulative %	88.148	**97.502**	100.000

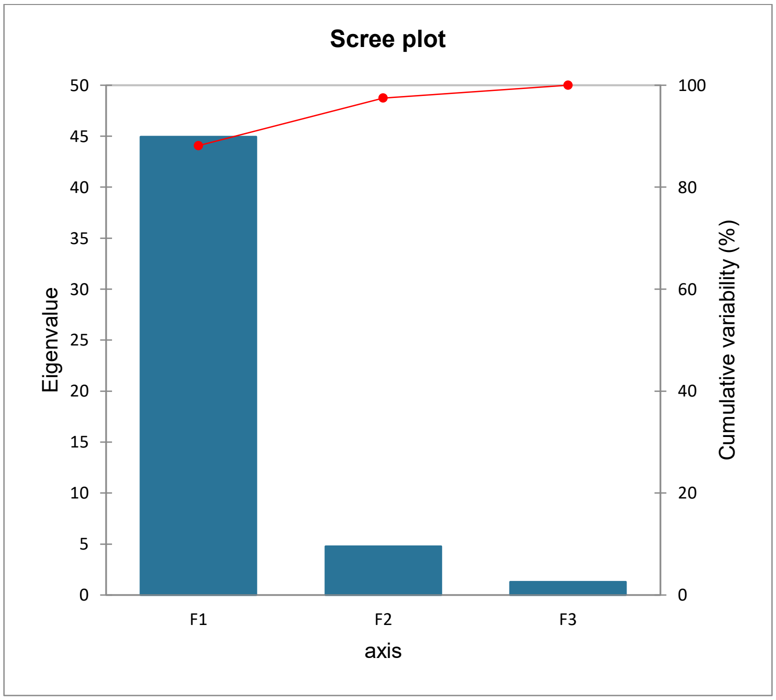

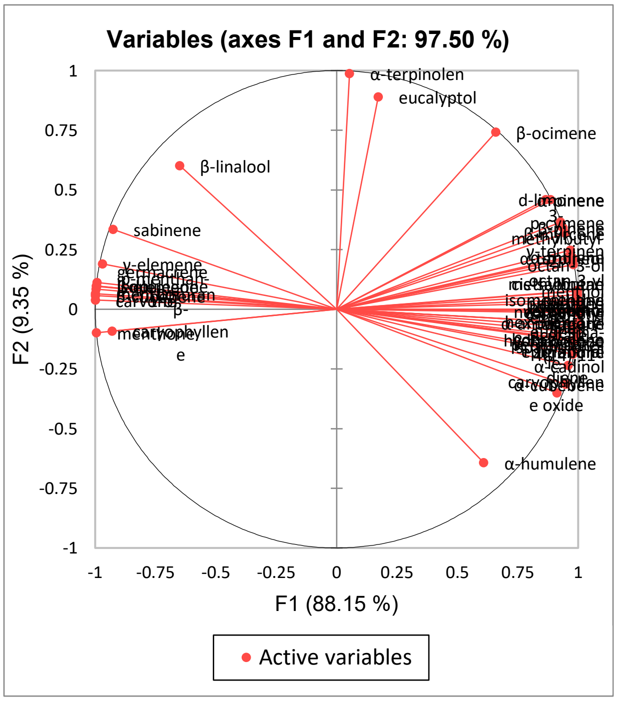

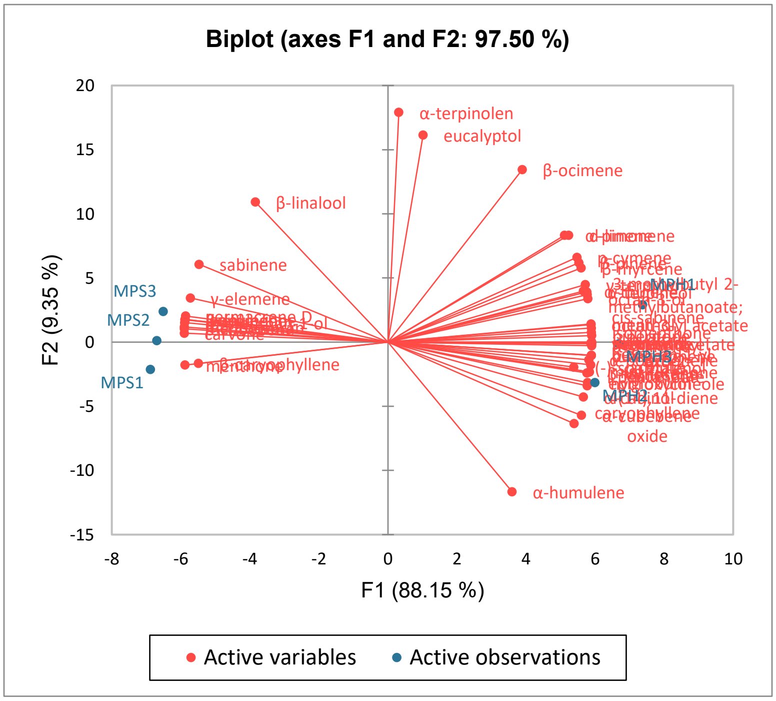

Biplot (axes F1 and F2: 97.50 %)
α-terpinolen
eucalyptol
β-ocimene
β-linalool
sabinene
MPS3
γ-elemene
MPS2
germacrene D
carvone
MPS1
β-caryophyllene
menthone
p-cymene
β-myrcene
3-methylbutyl 2-
methylbutanoate;
octan-3-ol
cis-sabinene
acetate
MPH1
MPH3
MPH2
caryophyllene
oxide
α-cubebene
α-humulene
20
15
10
5
0
-5
-10
-15
-8
-6
-4
-2
0
2
4
6
8
10
F1 (88.15 %)
F2 (9.35 %)
Active variables
Active observations

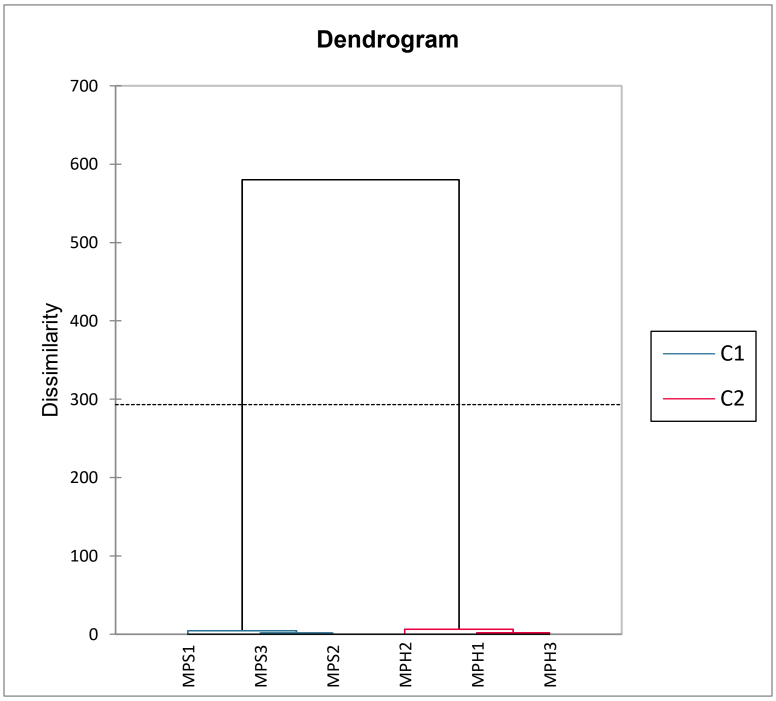
Dendrogram
Dissimilarity
700
600
500
400
300
200
100
0
MPS1
MPS3
MPS2
MPH2
MPH1
MPH3
C1
C2

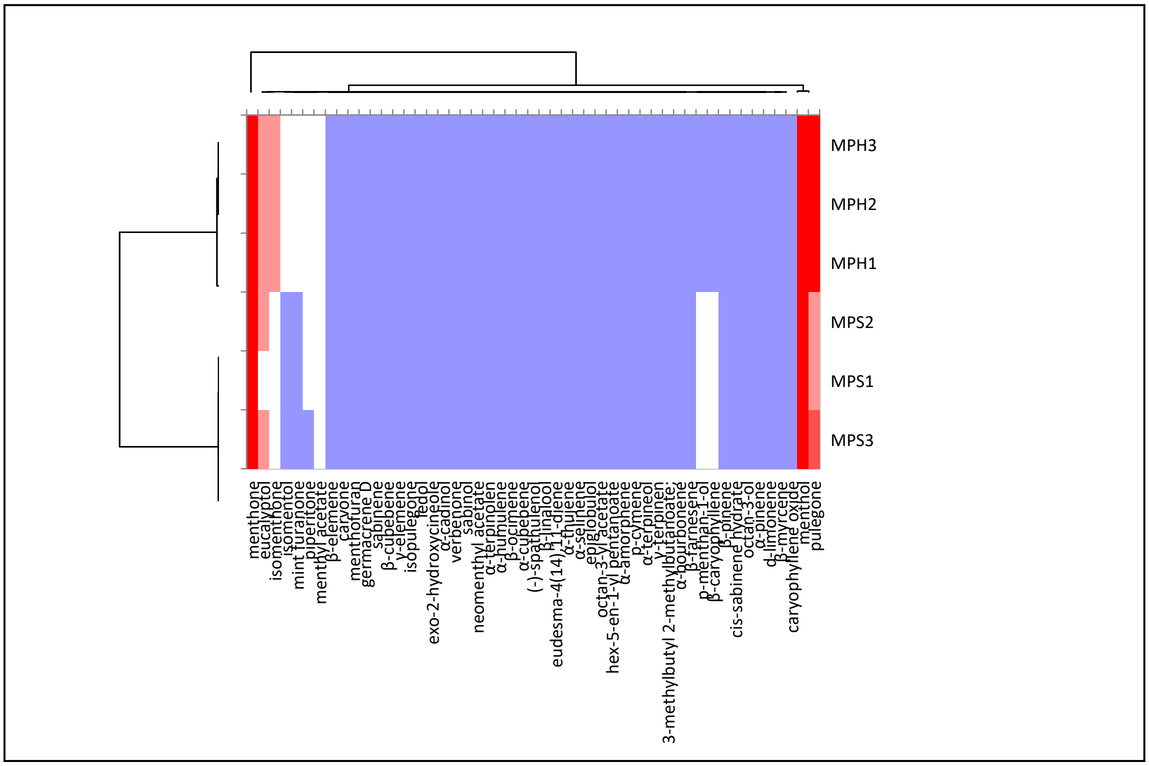
MPH3
MPH2
MPH1
MPS2
MPS1
MPS3
menthone
eucalyptol
isomenthone
isomentol
mint furanone
piperitone
menthyl acetate
β-elemene
carvone
menthofuran
germacrene D
sabinene
β-cubebene
γ-elemene
isopulegone
ledol
exo-2-hydroxycineole
α-cadinol
verbenone
sabinol
neomenthyl acetate
α-terpinolen
α-humulene
β-ocimene
α-cubebene
(-)-spathulenol
β-linalool
eudesma-4(14),11-diene
α-thujene
α-selinene
epiglobulol
octan-3-yl acetate
hex-5-en-1-yl pentanoate
α-amorphene
p-cymene
α-terpineol
γ-terpinen
3-methylbutyl 2-methylbutanoate
α-bourbonene
β-farnesene
p-menthan-1-ol
β-caryophyllene
β-pinene
cis-sabinene hydrate
octan-3-ol
α-pinene
d-limonene
β-myrcene
caryophyllene oxide
menthol
pulegone

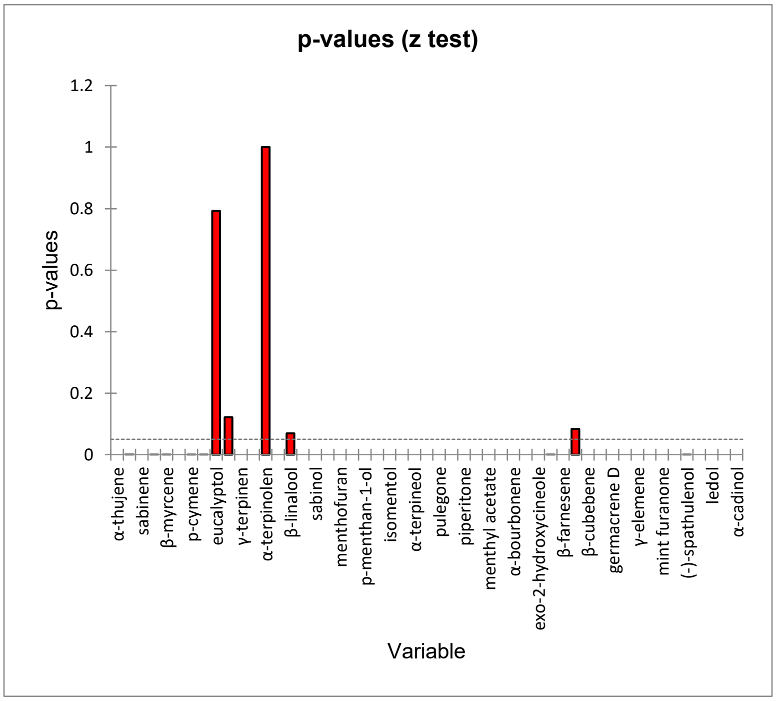

p-values (z test)
p-values
1.2
1
0.8
0.6
0.4
0.2
0
α-thujene
sabinene
β-myrcene
p-cymene
eucalyptol
γ-terpinen
α-terpinolen
β-linalool
sabinol
menthofuran
p-menthan-1-ol
isomentol
α-terpineol
pulegone
piperitone
menthyl acetate
α-bourbonene
exo-2-hydroxycineole
β-farnesene
β-cubebene
germacrene D
γ-elemene
mint furanone
(-)-spathulenol
ledol
α-cadinol
Variable

Statistic	Comp1
Q^2 cum	**0.994**
R^2Y cum	0.997
R^2X cum	0.881

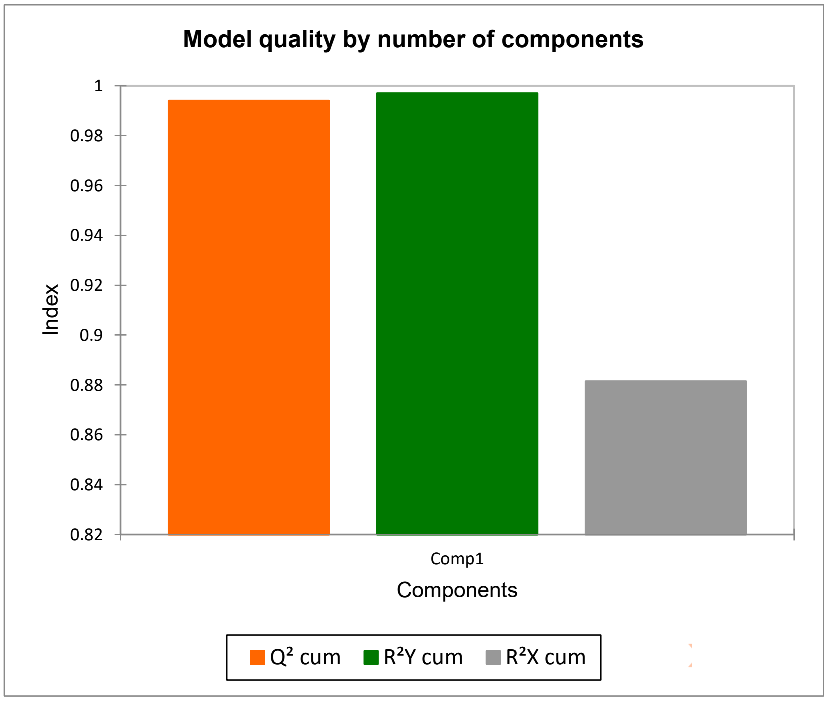

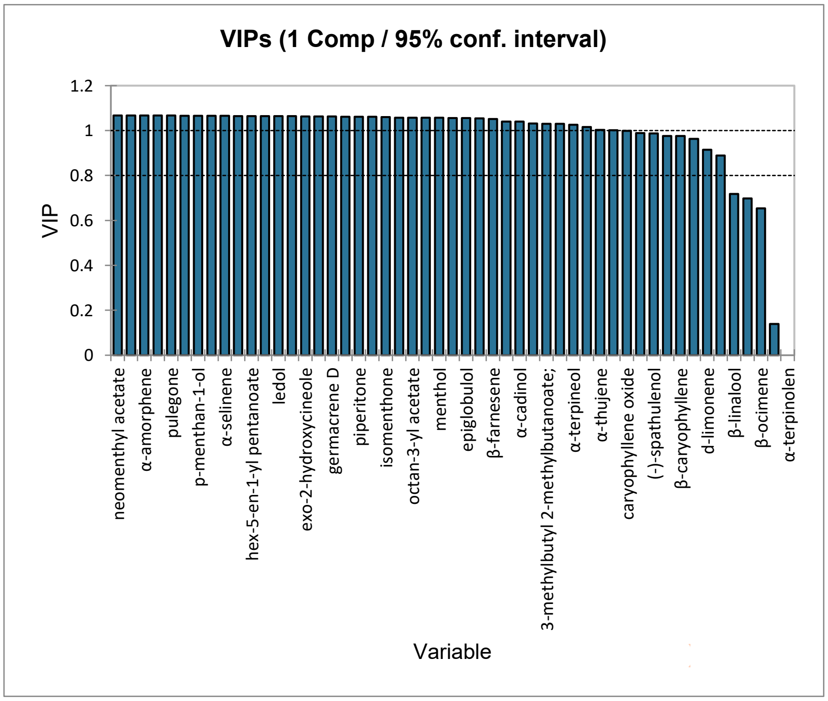

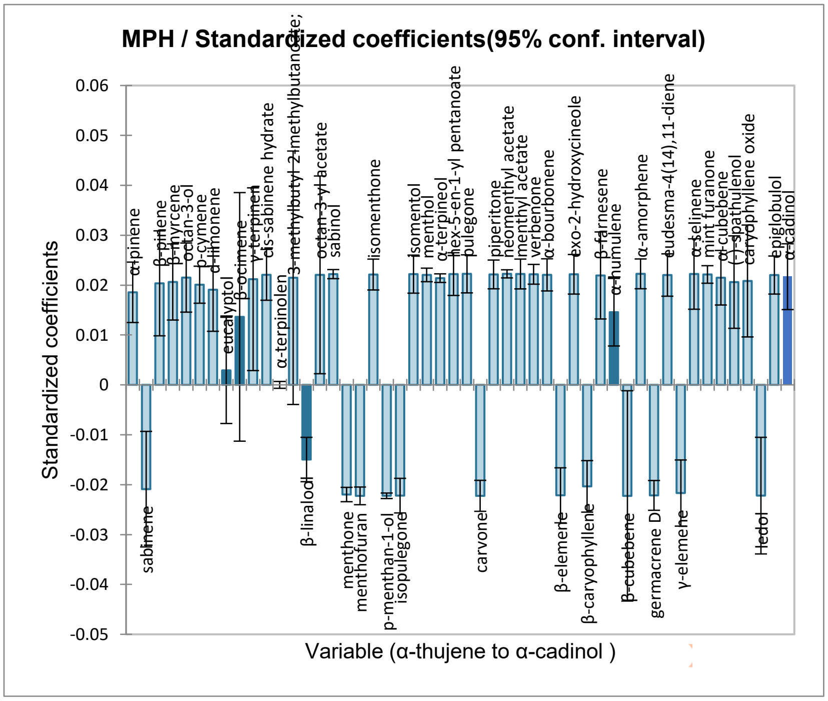

MPH / Standardized coefficients(95% conf. interval)
Standardized coefficients
0.06
0.05
0.04
0.03
0.02
0.01
0
-0.01
-0.02
-0.03
-0.04
-0.05
Variable (α-thujene to α-cadinol)
α-pinene
sabinene
β-pinene
β-myrcene
octan-3-ol
p-cymene
d-limonene
eucalyptol
β-ocimene
γ-terpinen
cis-sabinene hydrate
α-terpinolen
3-methylbutyl 2-methylbutanoate;
β-linalool
octan-3-yl acetate
sabinol
menthone
menthofuran
isomenthone
p-menthan-1-ol
isopulegone
isomentol
menthol
α-terpineol
hex-5-en-1-yl pentanoate
pulegone
carvone
piperitone
neomenthyl acetate
menthyl acetate
verbenone
α-bourbonene
β-elemene
exo-2-hydroxycineole
β-caryophyllene
β-farnesene
α-humulene
β-cubebene
α-amorphene
germacrene D
eudesma-4(14),11-diene
γ-elemene
α-selinene
mint furanone
α-cubebene
(-)-spathulenol
caryophyllene oxide
Ledol
epiglobulol
α-cadinol

Unsupervised Principal Component Analysis (PCA) and Hierarchical Cluster Analysis (HCA) for *Achillea millefolium*

	F1	F2	F3
Eigenvalue	66.933	2.437	1.630
Variability (%)	94.272	3.432	2.295
Cumulative %	94.272	97.705	100.000

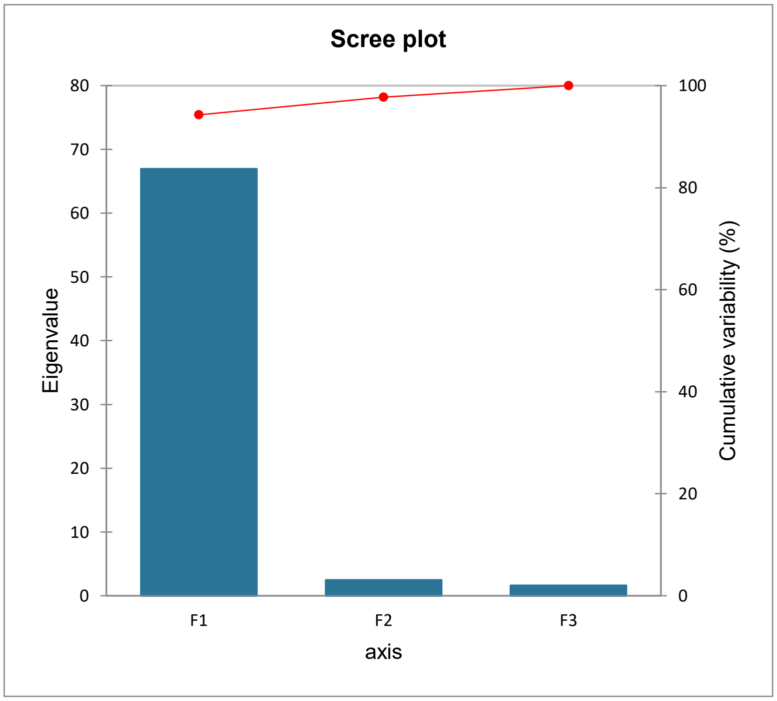

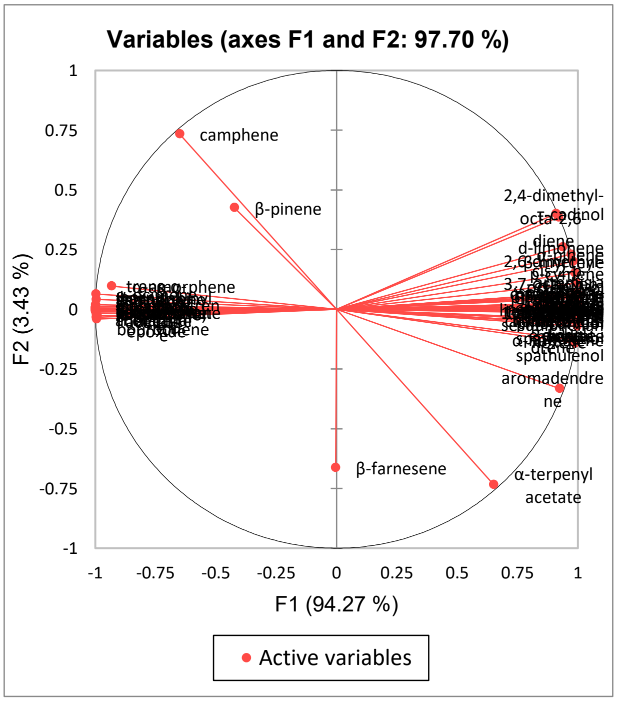

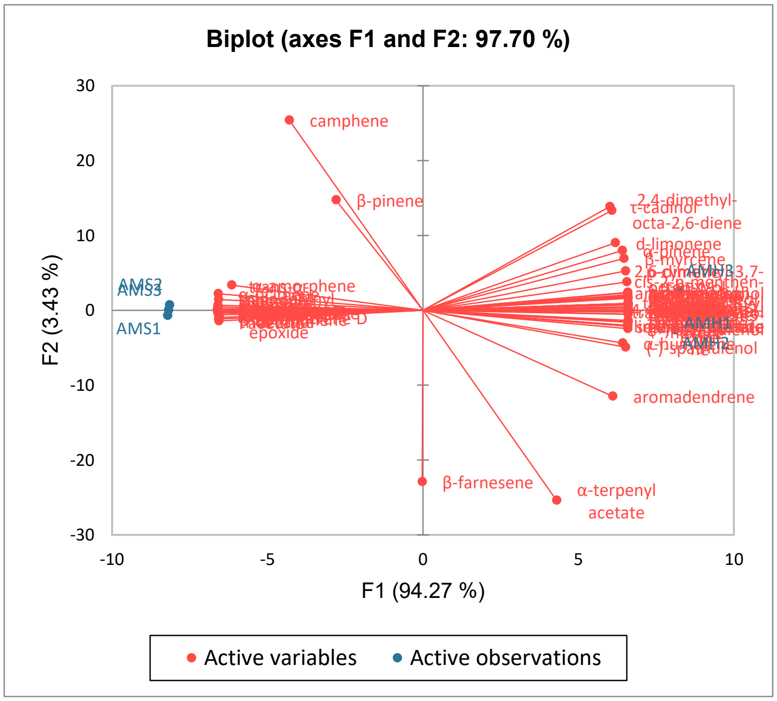

Biplot (axes F1 and F2: 97.70 %)
camphene
β-pinene
2,4-dimethyl-
octa-2,6-diene
d-limonene
aromadendrene
β-farnesene
α-terpenyl
acetate
epoxide
AMS2
AMS3
AMS1
AMH1
AMH2
F2 (3.43 %)
F1 (94.27 %)
30
20
10
0
-10
-20
-30
-10
-5
0
5
10
Active variables
Active observations

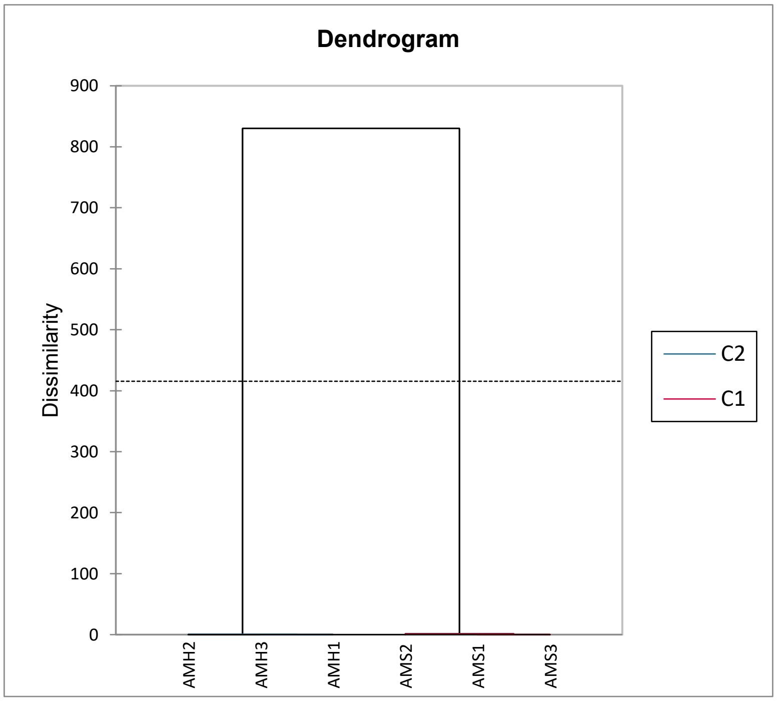

Dendrogram
900
800
700
600
500
400
300
200
100
0
Dissimilarity
AMH2
AMH3
AMH1
AMS2
AMS1
AMS3
C2
C1

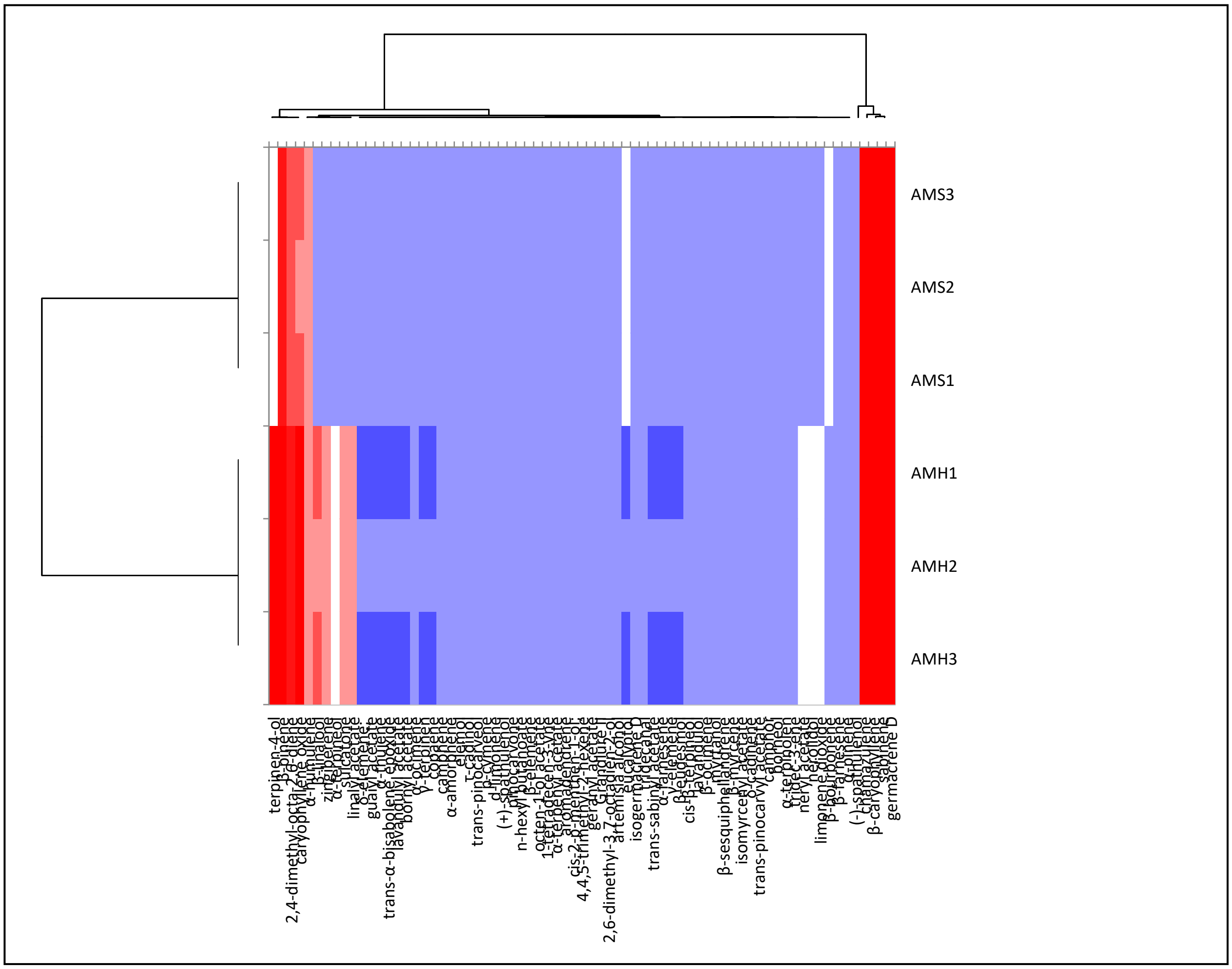
AMS3
AMS2
AMS1
AMH1
AMH2
AMH3

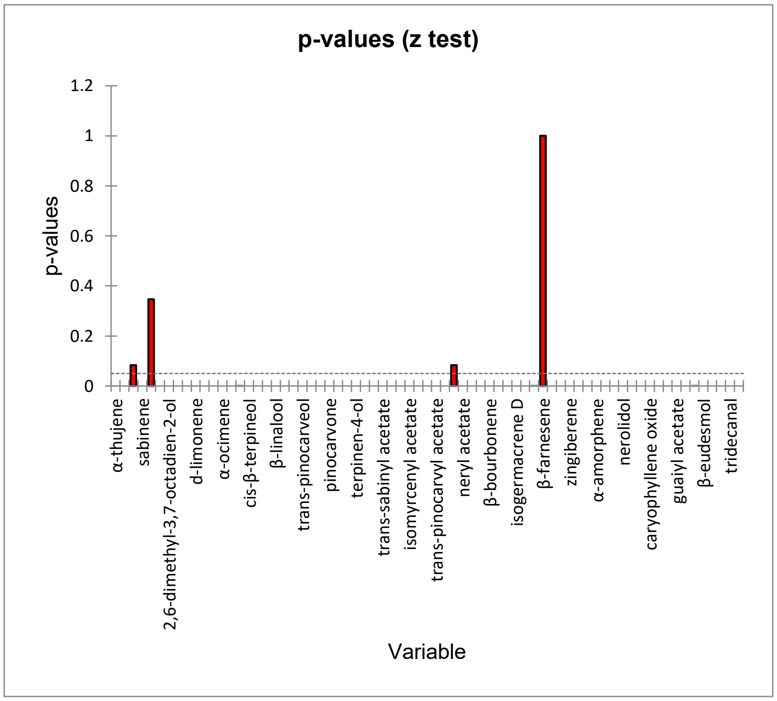

p-values (z test)
p-values
1.2
1
0.8
0.6
0.4
0.2
0
α-thujene
sabinene
2,6-dimethyl-3,7-octadien-2-ol
d-limonene
α-ocimene
cis-β-terpineol
β-linalool
trans-pinocarveol
pinocarvone
terpinen-4-ol
trans-sabinyl acetate
isomyrcenyl acetate
trans-pinocaryl acetate
neryl acetate
β-bourbonene
isogermacrene D
β-farnesene
zingiberene
α-amorphene
nerolidol
caryophyllene oxide
guaiyl acetate
β-eudesmol
tridecanal
Variable

Statistic	Comp1
Q² cum	0.9996348
R²Y cum	0.999974
R²X cum	0.9427227

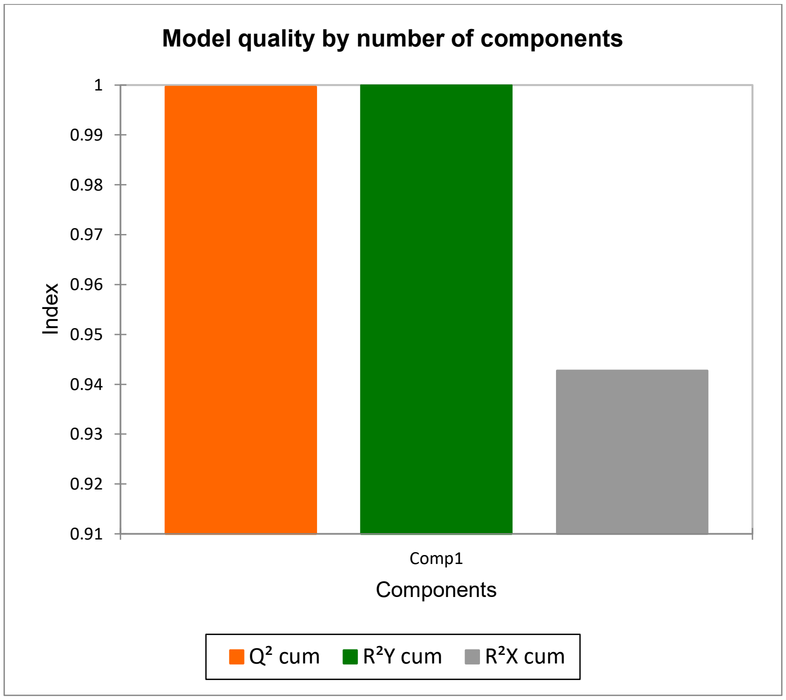

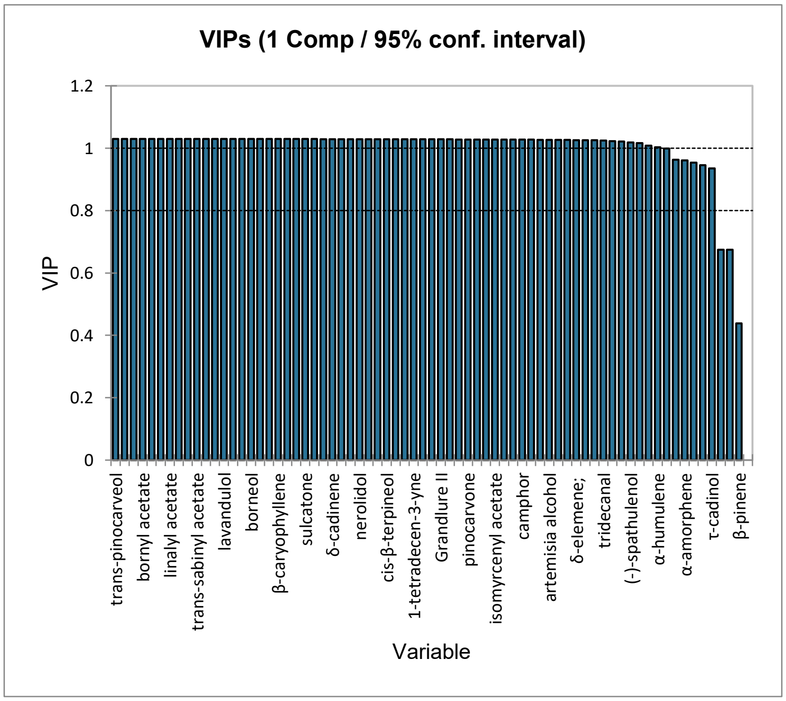

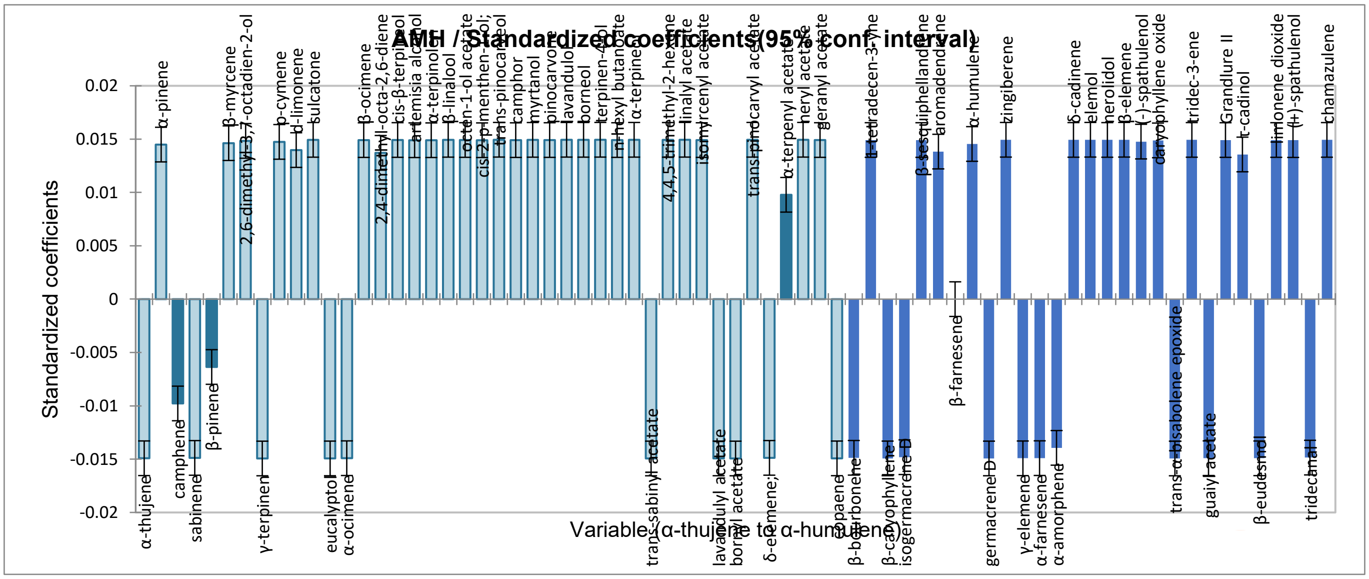

AMH / Standardized coefficients (95% conf. interval)
Standardized coefficients
Variable (α-thujene to α-humulene)
0.02
0.015
0.01
0.005
0
-0.005
-0.01
-0.015
-0.02
α-thujene
α-pinene
camphene
sabinene
β-pinene
β-myrcene
2,6-dimethyl-3,7-octadien-2-ol
γ-terpinene
p-cymene
d-limonene
sulcatone
eucalyptol
α-ocimene
β-ocimene
2,4-dimethyl-octa-2,6-diene
cis-β-terpineol
artemisia alcohol
α-terpinolene
β-linalool
octen-1-ol acetate
trans-pinocarveol
camphor
myrtanol
pinocarvone
lavandulol
borneol
terpinen-4-ol
n-hexyl butanoate
α-terpineol
trans-sabinyl acetate
4,4,5-trimethyl-2-hexene
linalyl acetate
isomyrcenyl acetate
lavandulyl acetate
bornyl acetate
trans-pinocarvyl acetate
δ-elemene
α-terpenyl acetate
neryl acetate
geranyl acetate
copaene
β-bourbonene
β-caryophyllene
isogermacrene D
β-sesquiphellandrene
aromadendrene
β-farnesene
α-humulene
germacrene D
zingiberene
γ-elemene
α-farnesene
α-amorphene
δ-cadinene
elemol
nerolidol
β-elemene
(-)-spathulenol
caryophyllene oxide
trans-α-bisabolene epoxide
tridec-3-ene
guaiyl acetate
Grandlure II
τ-cadinol
β-eudesmol
limonene dioxide
(+)-spathulenol
tridecanal
chamazulene

Unsupervised Principal Component Analysis (PCA) and Hierarchical Cluster Analysis (HCA) for *Mentha spicata*

	F1	F2	F3
Eigenvalue	43.033	1.308	0.659
Variability (%)	95.629	2.908	1.463
Cumulative %	95.629	98.537	100.000

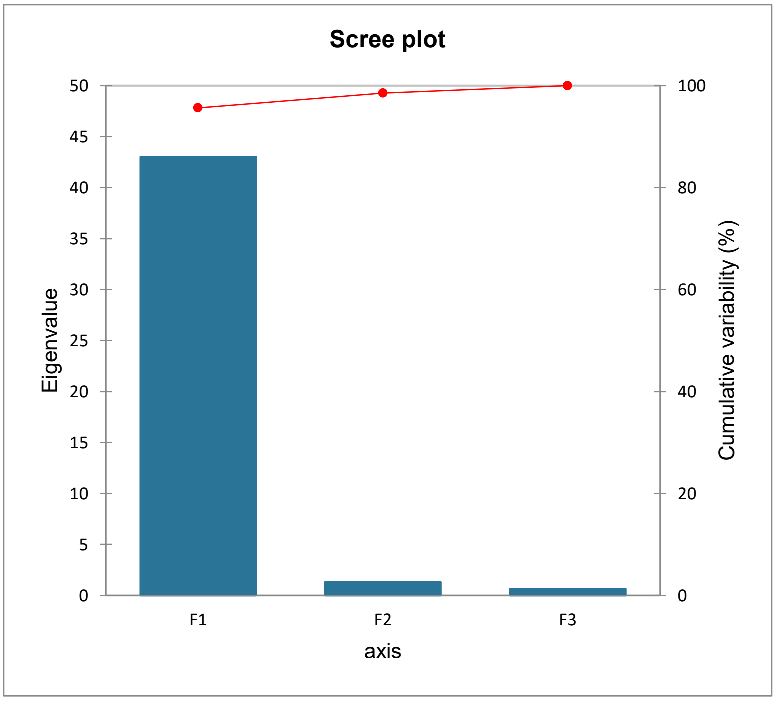

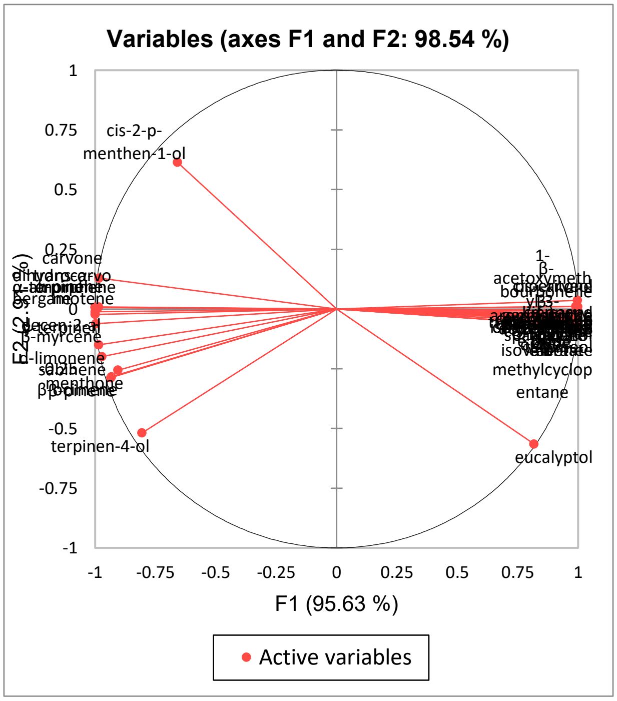

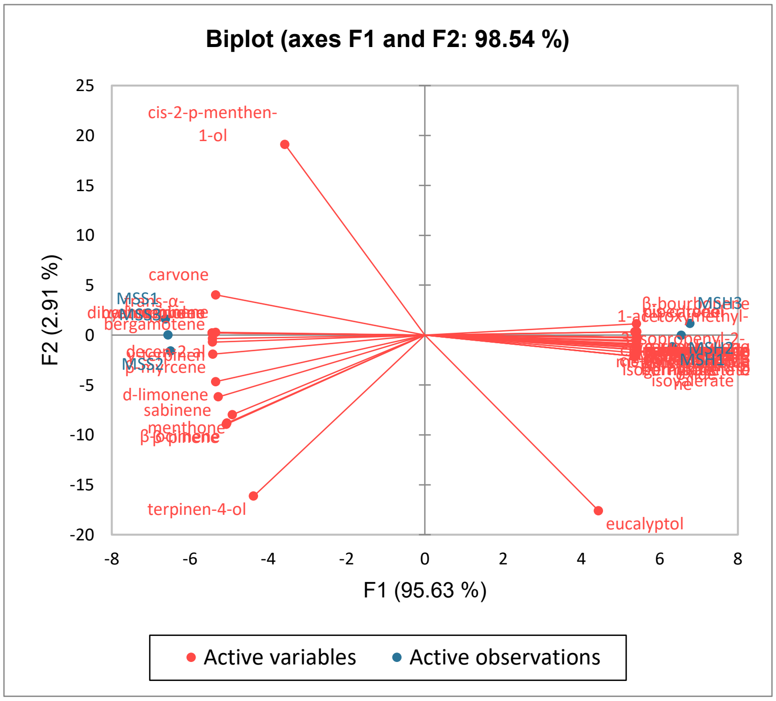

Biplot (axes F1 and F2: 98.54 %)
cis-2-p-menthen-
1-ol
carvone
MSS1
bergamotene
MSS2
d-limonene
sabinene
menthone
terpinen-4-ol
eucalyptol
MSH3
isovalerate
F1 (95.63 %)
F2 (2.91 %)
Active variables
Active observations

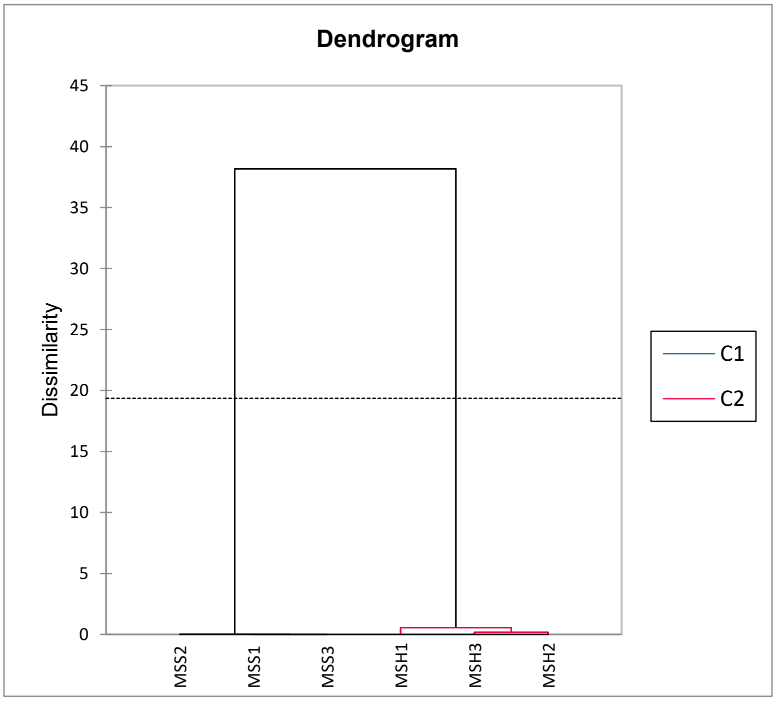

Dendrogram
Dissimilarity
45
40
35
30
25
20
15
10
5
0
MSS2
MSS1
MSS3
MSH1
MSH3
MSH2
C1
C2

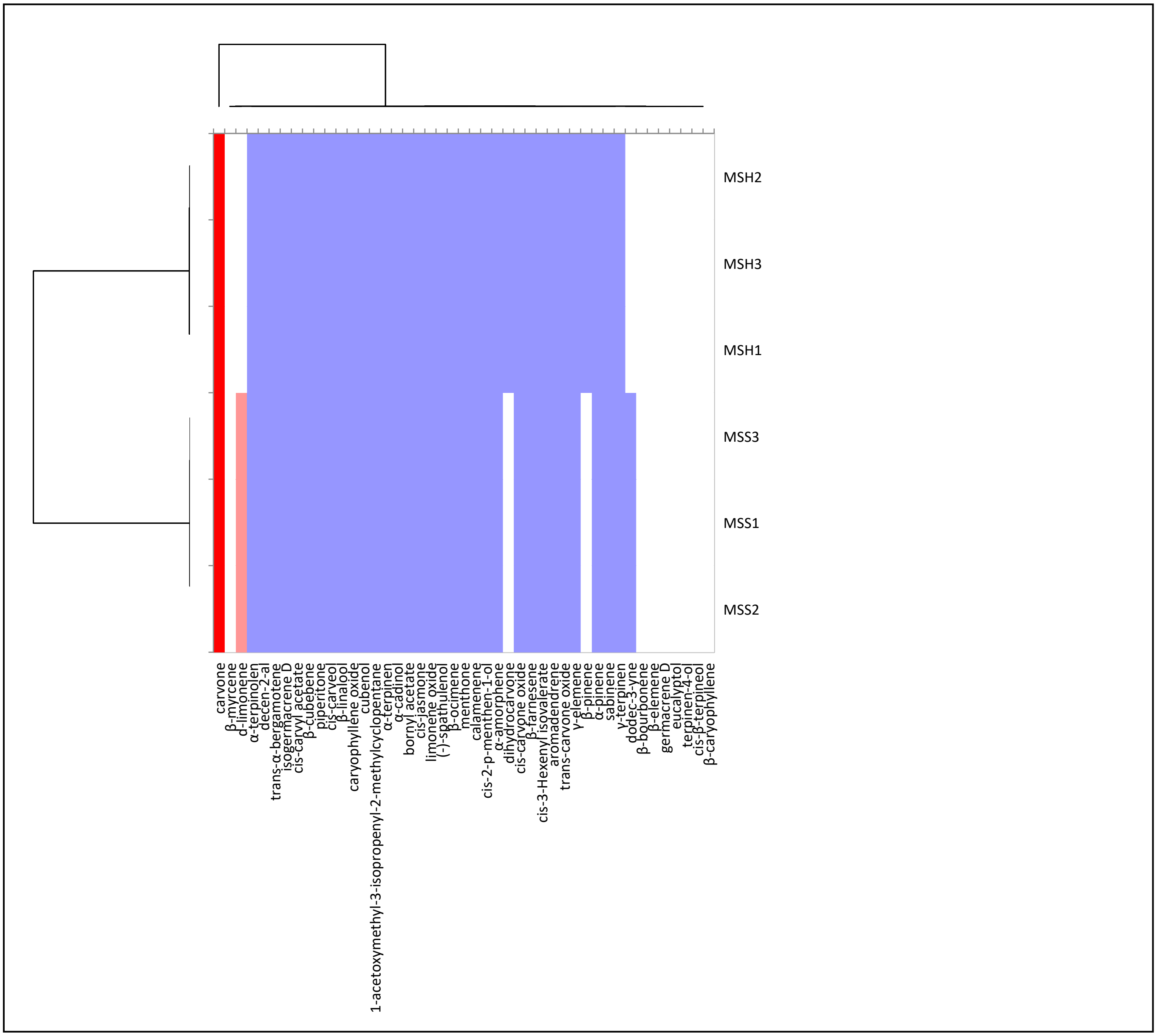
MSH2
MSH3
MSH1
MSS3
MSS1
MSS2
carvone
β-myrcene
d-limonene
α-terpinolen
decen-2-al
trans-α-bergamotene
isogermacrene D
cis-carvyl acetate
β-cubebene
piperitone
cis-carveol
β-linalool
caryophyllene oxide
cubenol
1-acetoxymethyl-3-isopropenyl-2-methylcyclopentane
α-terpinen
α-cadinol
bornyl acetate
cis-jasmone
limonene oxide
(-)-spathulenol
β-ocimene
menthone
calamenene
cis-2-p-menthen-1-ol
α-amorphene
dihydrocarvone
cis-carvone oxide
β-farnesene
cis-3-Hexenyl isovalerate
aromadendrene
trans-carvone oxide
γ-elemene
β-pinene
α-pinene
sabinene
γ-terpinen
dodec-3-yne
β-bourbonene
β-elemene
germacrene D
eucalyptol
terpinen-4-ol
cis-β-terpineol
β-caryophyllene

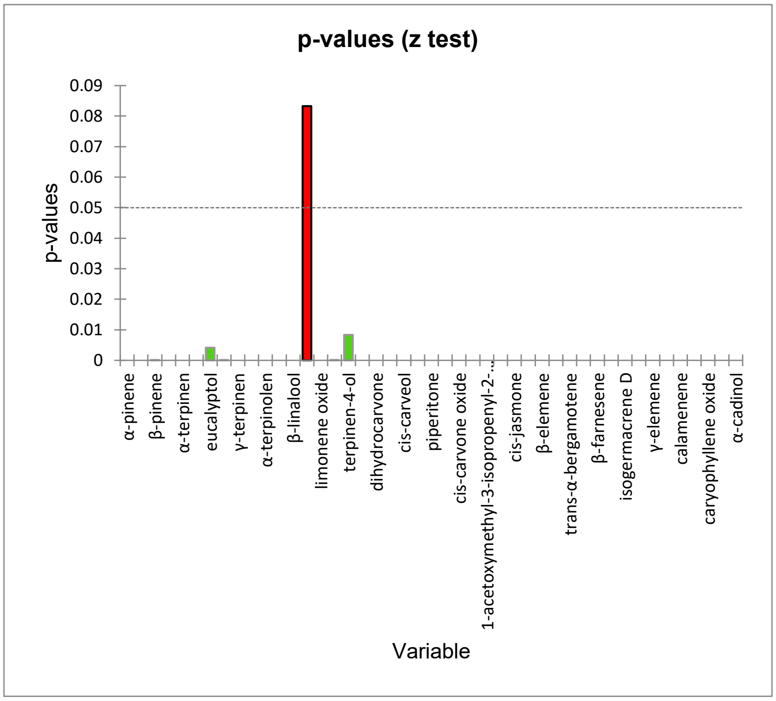

p-values (z test)
p-values
0.09
0.08
0.07
0.06
0.05
0.04
0.03
0.02
0.01
0
α-pinene
β-pinene
α-terpinen
eucalyptol
γ-terpinen
α-terpinolen
β-linalool
limonene oxide
terpinen-4-ol
dihydrocarvone
cis-carveol
piperitone
cis-carvone oxide
1-acetoxymethyl-3-isopropenyl-2-...
cis-jasmone
β-elemene
trans-α-bergamotene
β-farnesene
isogermacrene D
γ-elemene
calamenene
caryophyllene oxide
α-cadinol
Variable

Statistic	Comp1
Q^2 cum	0.99923
R^2Y cum	0.99959
R^2X cum	0.956

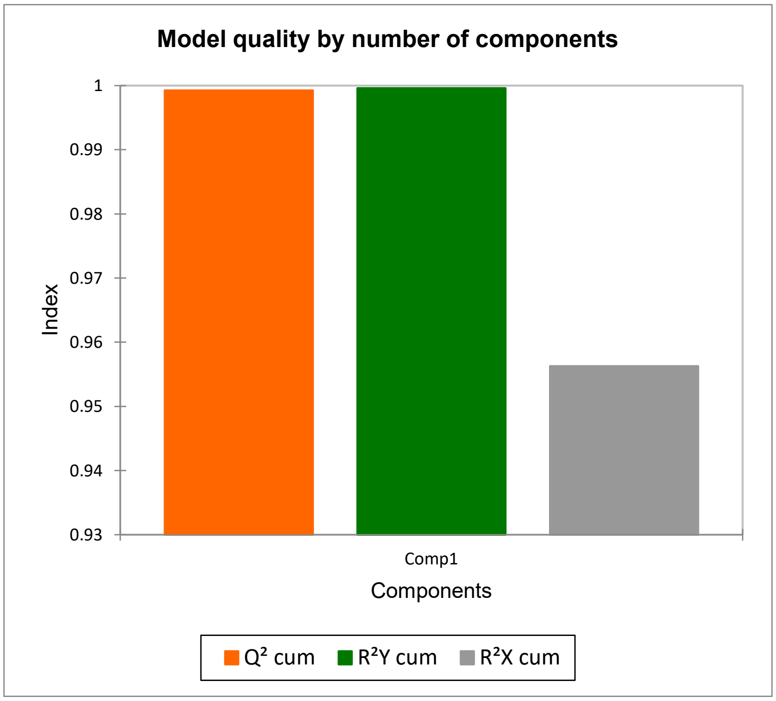

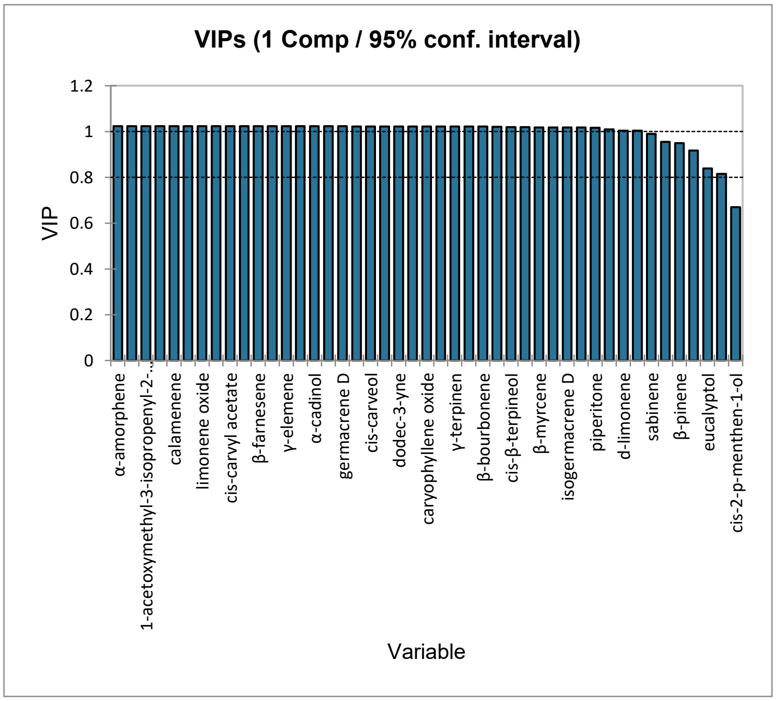

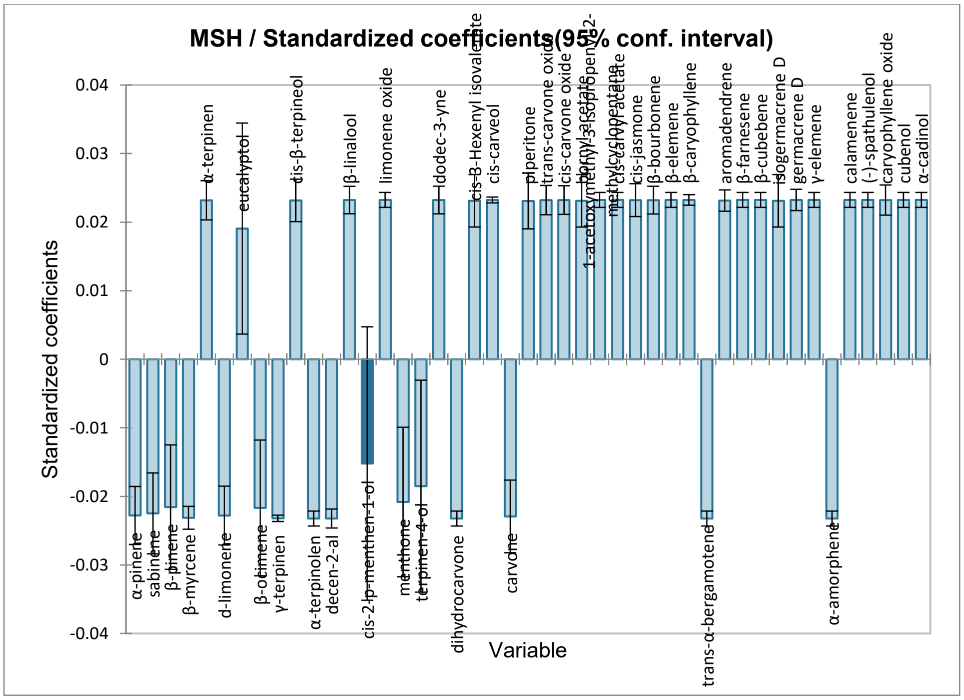

MSH / Standardized coefficients(95% conf. interval)
Standardized coefficients
Variable
0.04
0.03
0.02
0.01
0
-0.01
-0.02
-0.03
-0.04
α-pinene
sabinene
β-pinene
β-myrcene
α-terpinen
d-limonene
eucalyptol
β-ocimene
γ-terpinen
cis-β-terpineol
α-terpinolen
decen-2-al
β-linalool
limonene oxide
menthone
terpinen-4-ol
dodec-3-yne
dihydrocarvone
cis-β-Hexenyl isovalerate
cis-carveol
carvone
piperitone
trans-carvone oxide
cis-carvone oxide
bornyl acetate
cis-carvyl acetate
cis-jasmone
β-bourbonene
β-elemene
β-caryophyllene
trans-α-bergamotene
aromadendrene
β-farnesene
β-cubebene
bicyclogermacrene D
germacrene D
γ-elemene
α-amorphene
calamenene
(-)-spathulenol
caryophyllene oxide
cubenol
α-cadinol

Unsupervised Principal Component Analysis (PCA) and Hierarchical Cluster Analysis (HCA) for *Hyssopus officinalis*

	F1	F2	F3
Eigenvalue	45.773	4.887	1.340
Variability (%)	88.025	9.399	2.576
Cumulative %	88.025	97.424	100.000

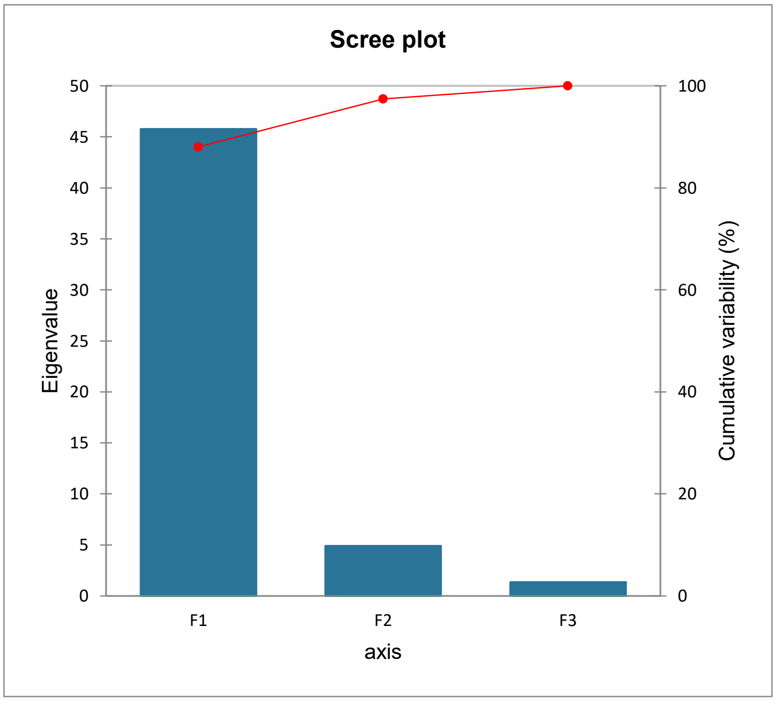

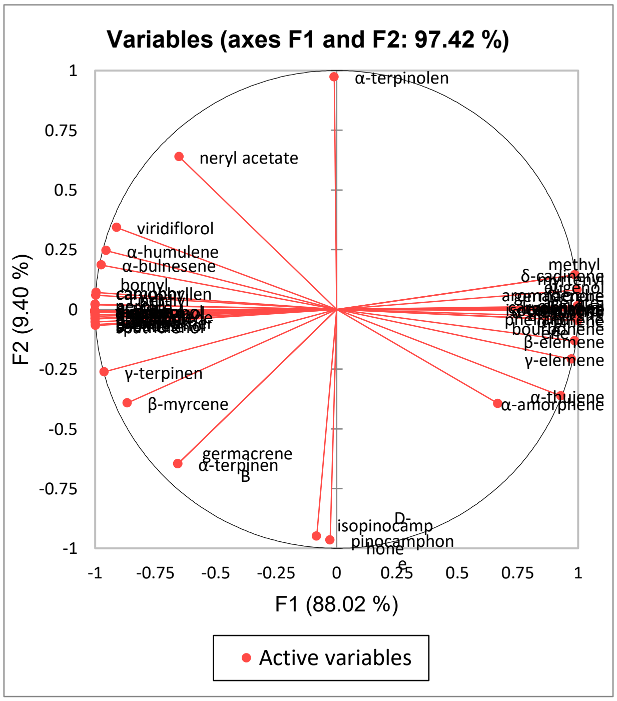

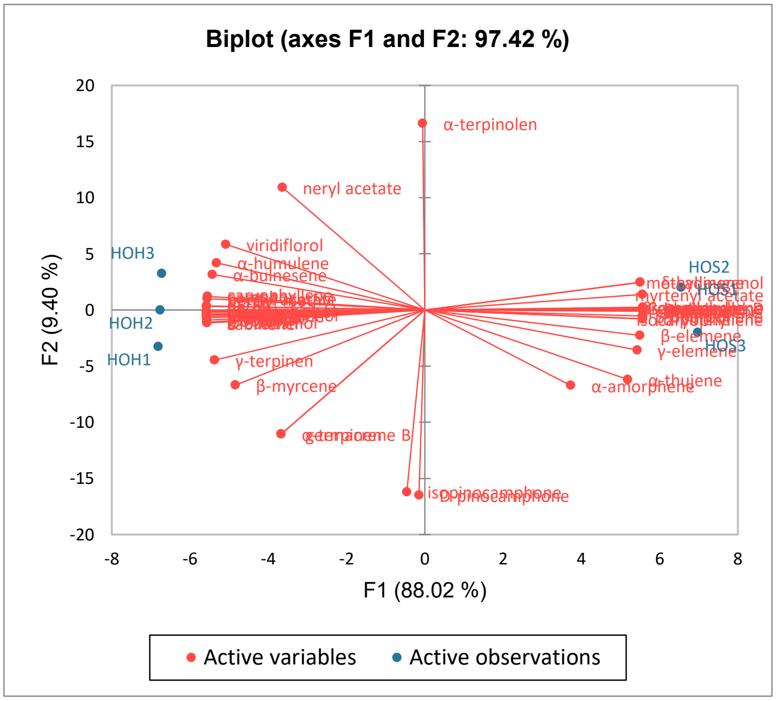

Biplot (axes F1 and F2: 97.42 %)
F2 (9.40 %)
F1 (88.02 %)
20
15
10
5
0
-5
-10
-15
-20
-8
-6
-4
-2
0
2
4
6
8
α-terpinolen
neryl acetate
viridiflorol
α-humulene
α-bulnesene
HOH3
HOH2
HOH1
γ-terpinen
β-myrcene
HOS2
HOS1
myrtenyl acetate
β-elemene
γ-elemene
HOS3
α-thujene
α-amorphene
Active variables
Active observations

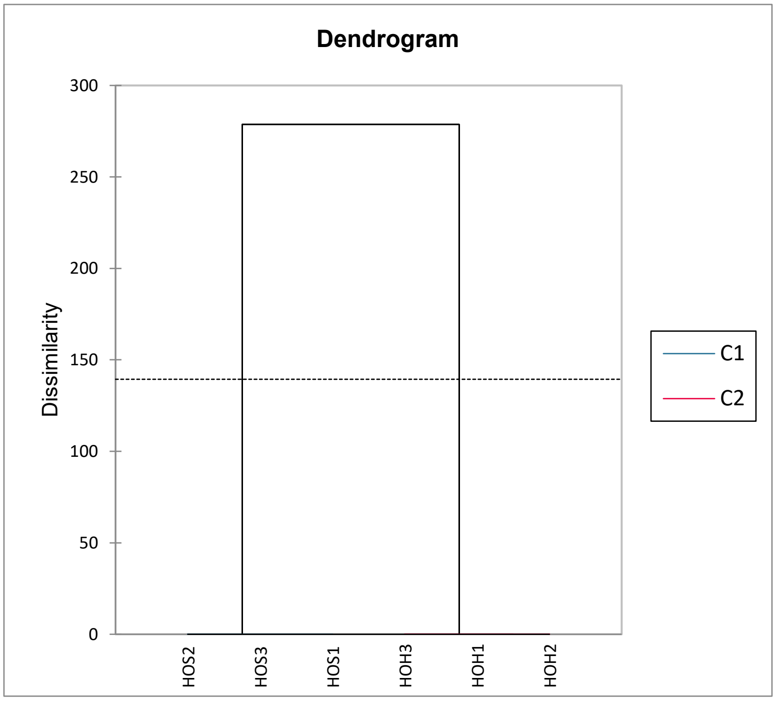
Dendrogram
Dissimilarity
300
250
200
150
100
50
0
HOS2
HOS3
HOS1
HOH3
HOH1
HOH2
C1
C2

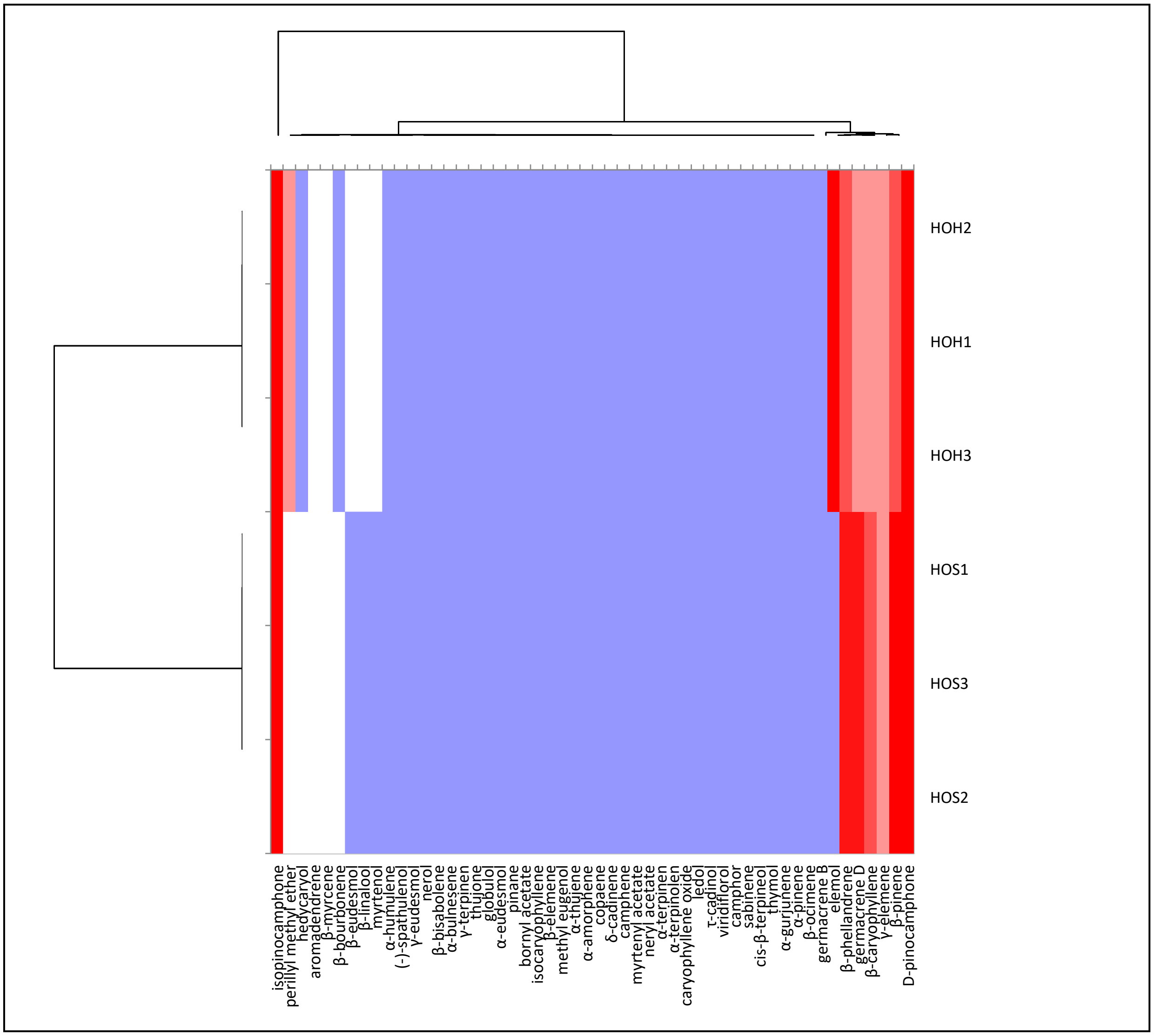
HOH2
HOH1
HOH3
HOS1
HOS3
HOS2
isopinocamphone
perillyl methyl ether
hedycaryol
aromadendrene
β-myrcene
β-bourbonene
β-eudesmol
β-linalool
myrtenol
α-humulene
(-)-spathulenol
γ-eudesmol
nerol
β-bisabolene
α-bulnesene
γ-terpinen
thujone
globulol
α-eudesmol
pinane
bornyl acetate
isocaryophyllene
β-elemene
methyl eugenol
α-thujene
α-amorphene
copaene
δ-cadinene
camphene
myrtenyl acetate
neryl acetate
α-terpinen
α-terpinolen
caryophyllene oxide
ledol
τ-cadinol
viridiflorol
camphor
sabinene
cis-β-terpineol
thymol
α-gurjunene
α-pinene
β-ocimene
germacrene B
elemol
β-phellandrene
germacrene D
β-caryophyllene
γ-elemene
β-pinene
D-pinocamphone

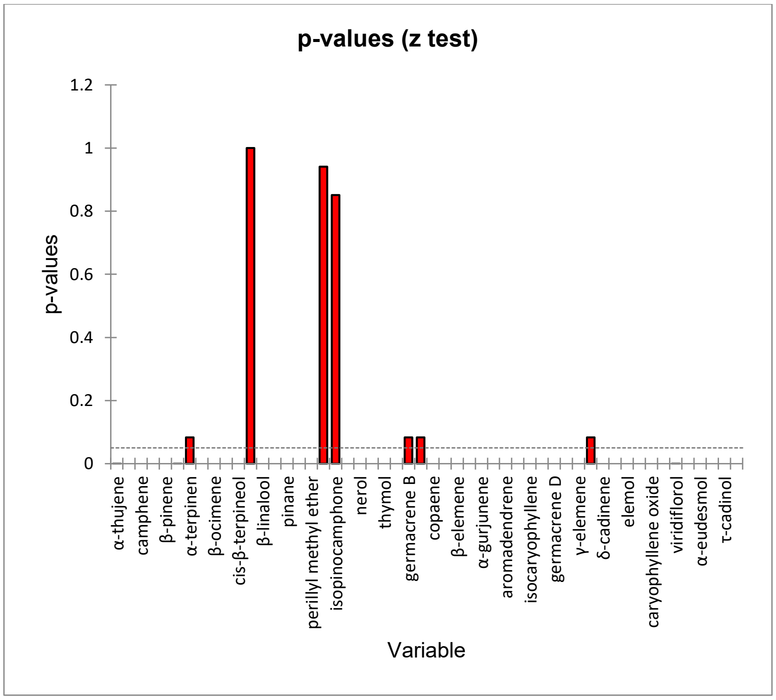
p-values (z test)
p-values
1.2
1
0.8
0.6
0.4
0.2
0
α-thujene
camphene
β-pinene
α-terpinen
β-ocimene
cis-β-terpineol
β-linalool
pinane
perillyl methyl ether
isopinocamphone
nerol
thymol
germacrene B
copaene
β-elemene
α-gurjunene
aromadendrene
isocaryophyllene
germacrene D
γ-elemene
δ-cadinene
elemol
caryophyllene oxide
viridiflorol
α-eudesmol
τ-cadinol
Variable

Statistic	Comp1
Q^2 cum	0.99785
R^2Y cum	0.999689
R^2X cum	0.8802466

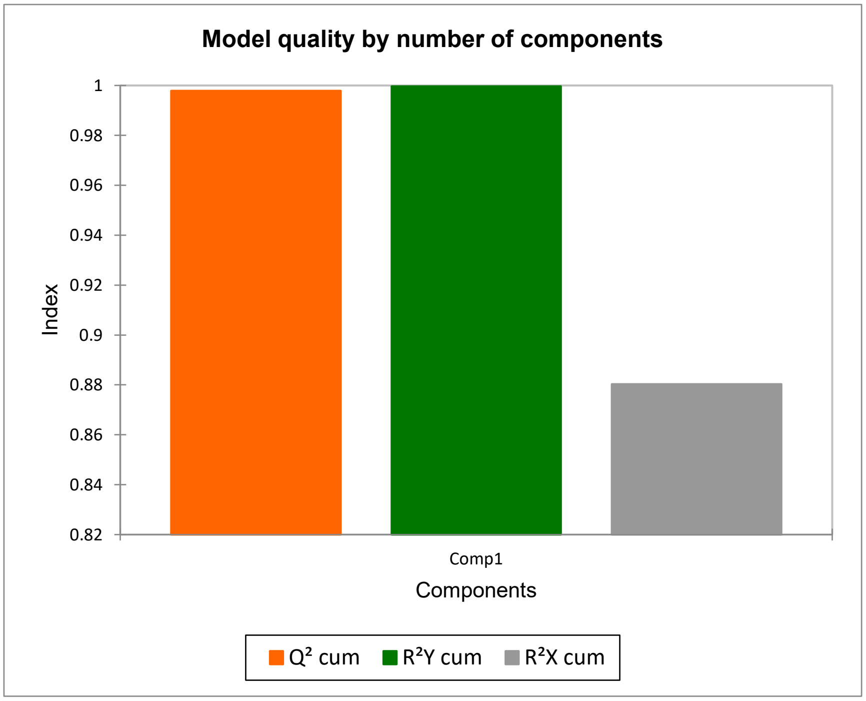

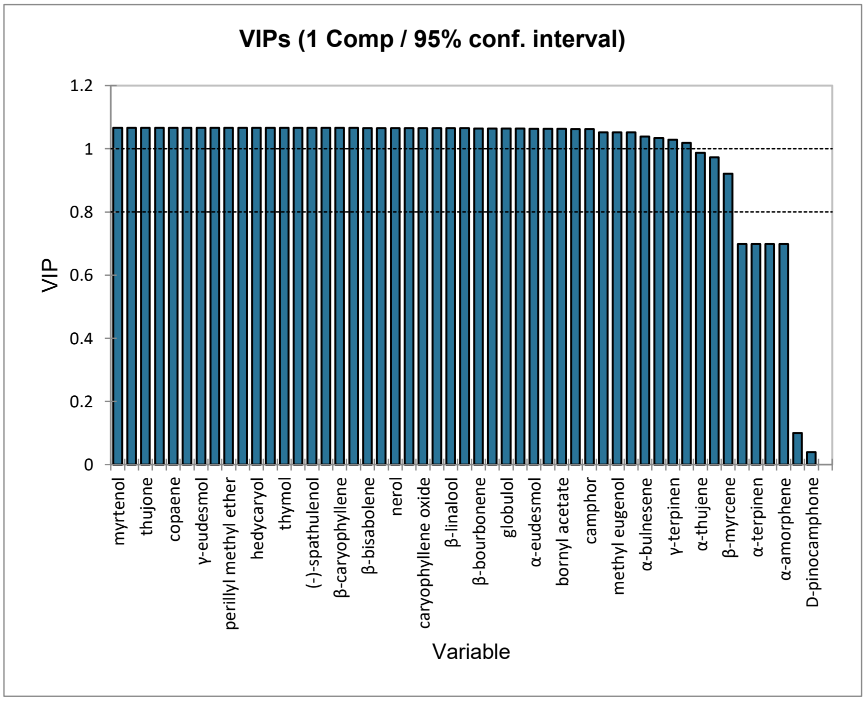

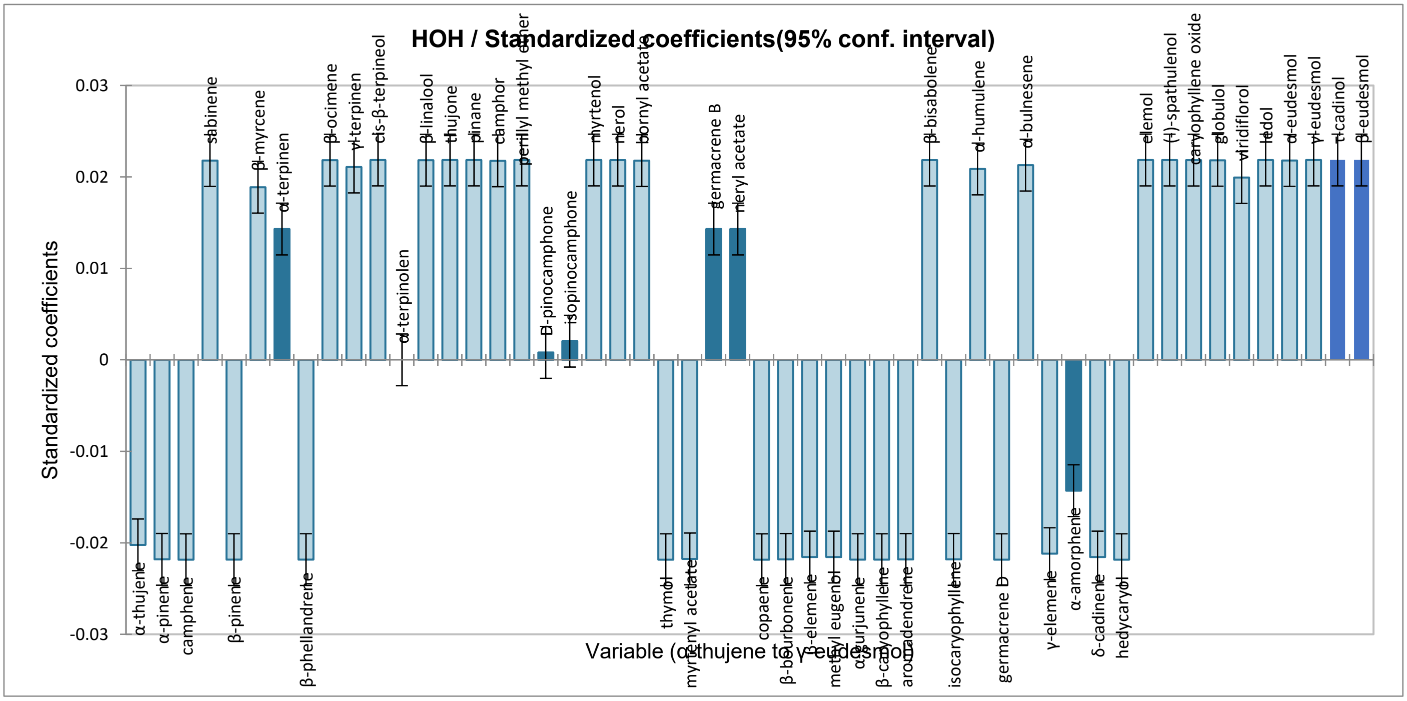

HOH / Standardized coefficients(95% conf. interval)
Standardized coefficients
0.03
0.02
0.01
0
-0.01
-0.02
-0.03
Variable (α-thujene to γ-eudesmol)
α-thujene
α-pinene
camphene
sabinene
β-pinene
β-myrcene
α-terpinen
β-phellandrene
β-ocimene
γ-terpinen
cis-β-terpineol
α-terpinolen
β-linalool
thujone
pinane
camphor
perillyl methyl ether
β-pinocamphone
isopinocamphone
myrtenol
nerol
bornyl acetate
thymol
myrtenyl acetate
germacrene B
neryl acetate
copaene
β-bourbonene
β-elemene
methyl eugenol
α-gurjunene
β-caryophyllene
aromadendrene
β-bisabolene
isocaryophyllene
α-humulene
germacrene D
α-bulnesene
γ-elemene
α-amorphene
δ-cadinene
hedycaryol
elemol
(-)-spathulenol
caryophyllene oxide
globulol
viridiflorol
ledol
α-eudesmol
γ-eudesmol
τ-cadinol
β-eudesmol

Unsupervised Principal Component Analysis (PCA) and Hierarchical Cluster Analysis (HCA) for *Picea abies*

	F1	F2	F3
Eigenvalue	78.053	0.698	0.248
Variability (%)	98.802	0.884	0.314
Cumulative %	98.802	99.686	100.000

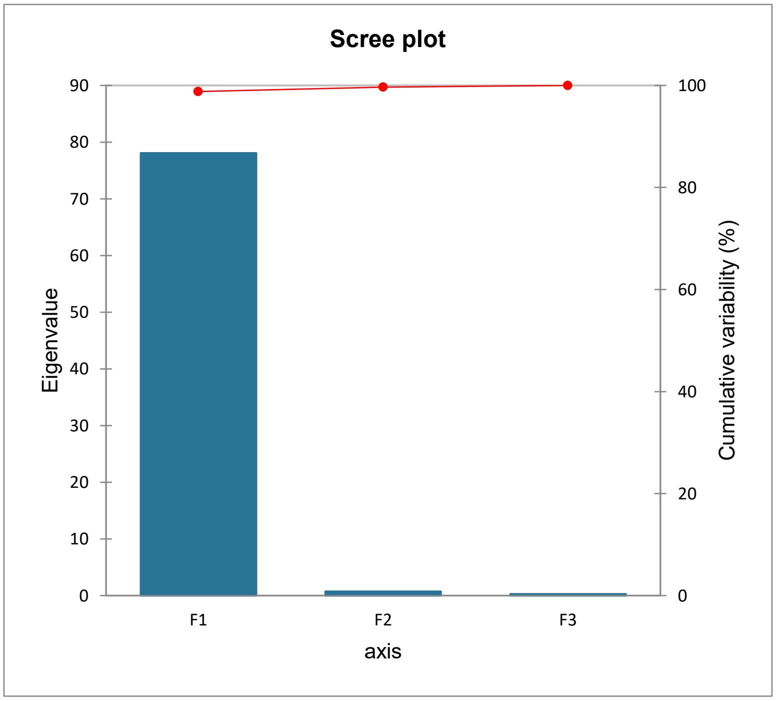

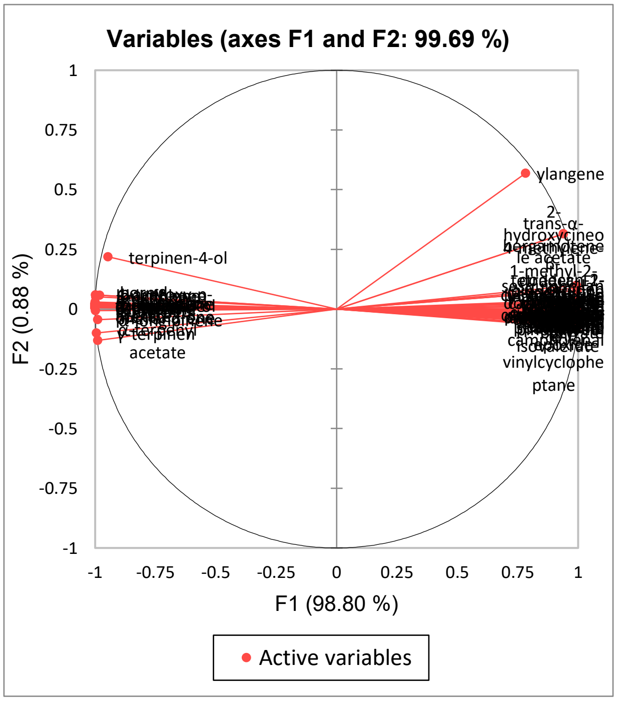

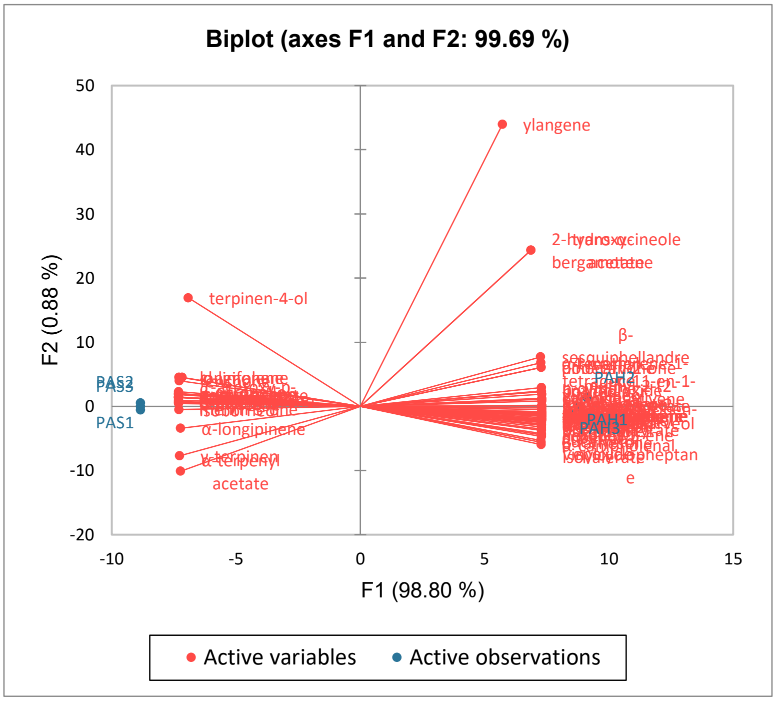
Biplot (axes F1 and F2: 99.69 %)
ylangene
terpinen-4-ol
α-longipinene
acetate
PAS1
PAH1
PAH3
F2 (0.88 %)
F1 (98.80 %)
Active variables
Active observations

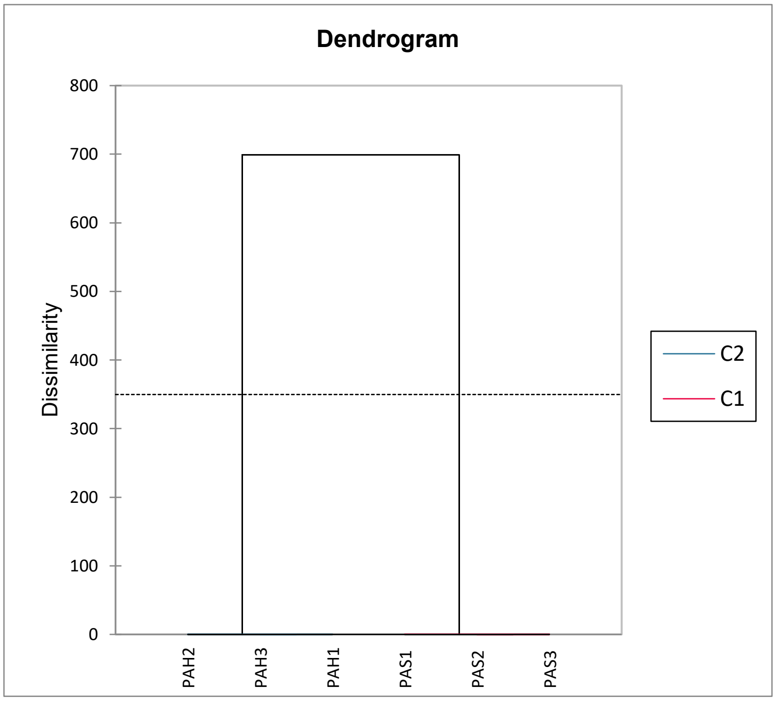
Dendrogram
Dissimilarity
800
700
600
500
400
300
200
100
0
PAH2
PAH3
PAH1
PAS1
PAS2
PAS3
C2
C1

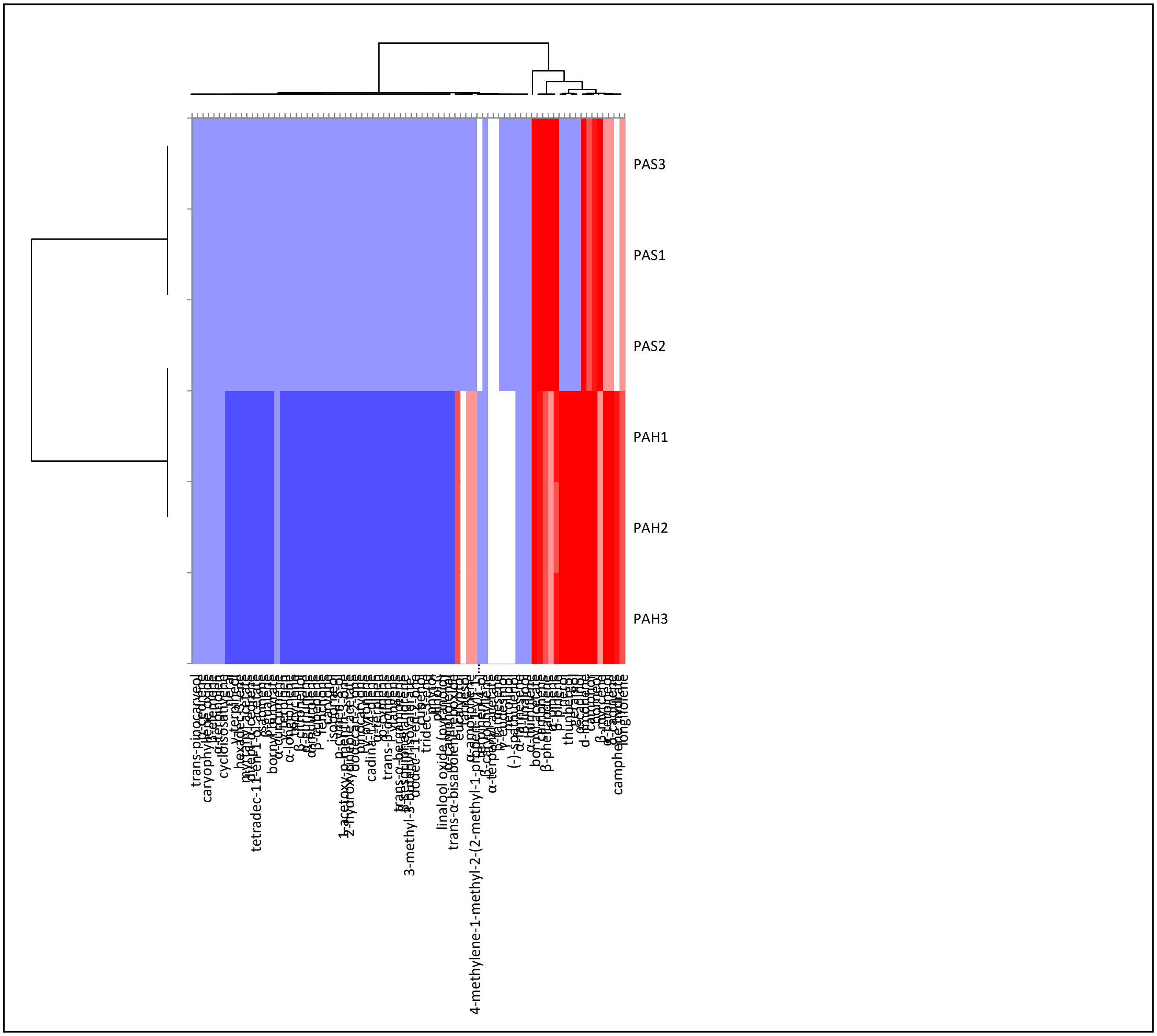
PAS3
PAS1
PAS2
PAH1
PAH2
PAH3
trans-pinocarveol
longifolene

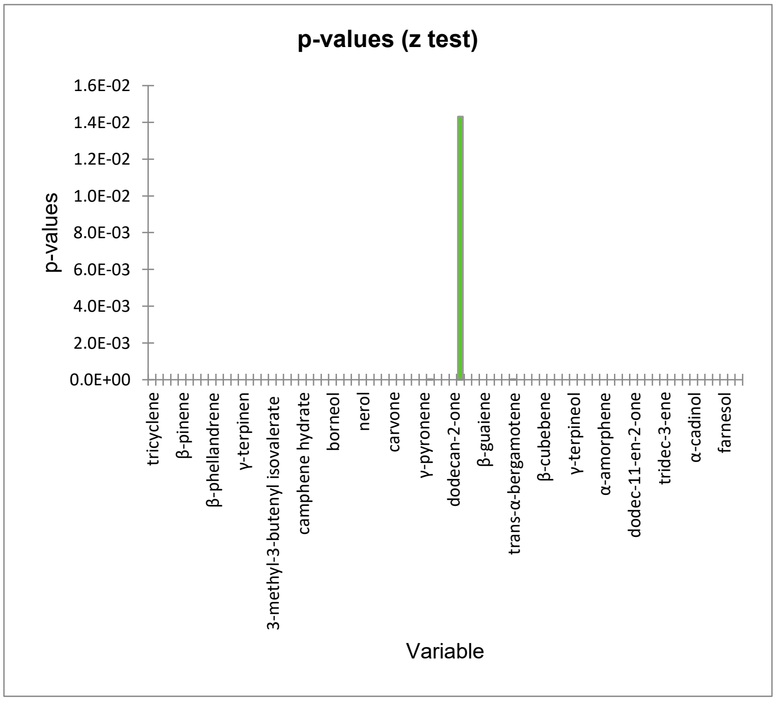

p-values (z test)
p-values
1.6E-02
1.4E-02
1.2E-02
1.0E-02
8.0E-03
6.0E-03
4.0E-03
2.0E-03
0.0E+00
tricyclene
β-pinene
β-phellandrene
γ-terpinen
3-methyl-3-butenyl isovalerate
camphene hydrate
borneol
nerol
carvone
γ-pyronene
dodecan-2-one
β-guaiene
trans-α-bergamotene
β-cubebene
γ-terpineol
α-amorphene
dodec-11-en-2-one
tridec-3-ene
α-cadinol
farnesol
Variable

Statistic	Comp1
Q^2 cum	0.99944401
R^2Y cum	0.99962293
R^2X cum	0.98801862

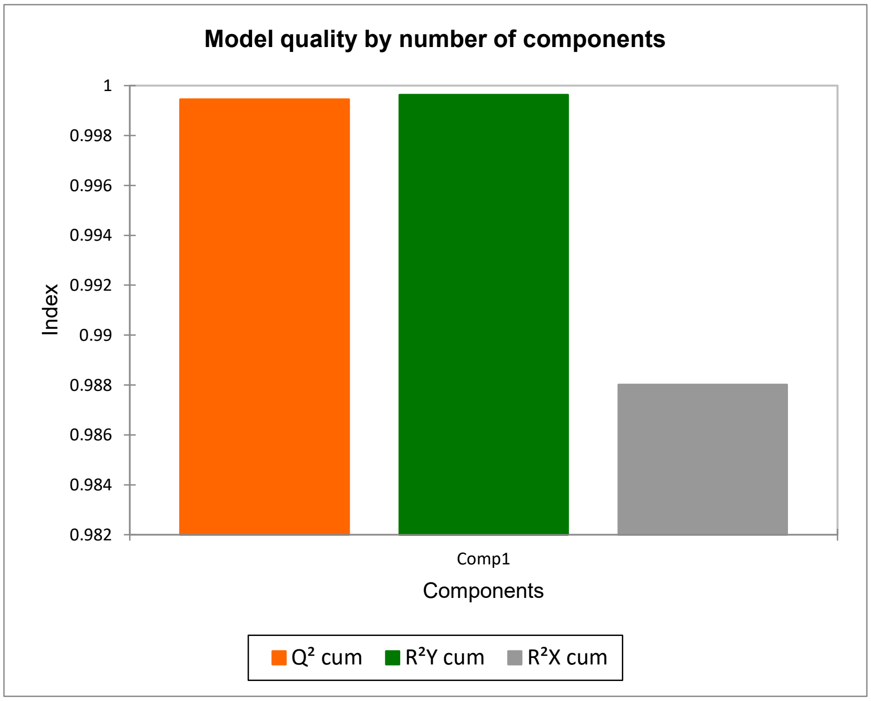

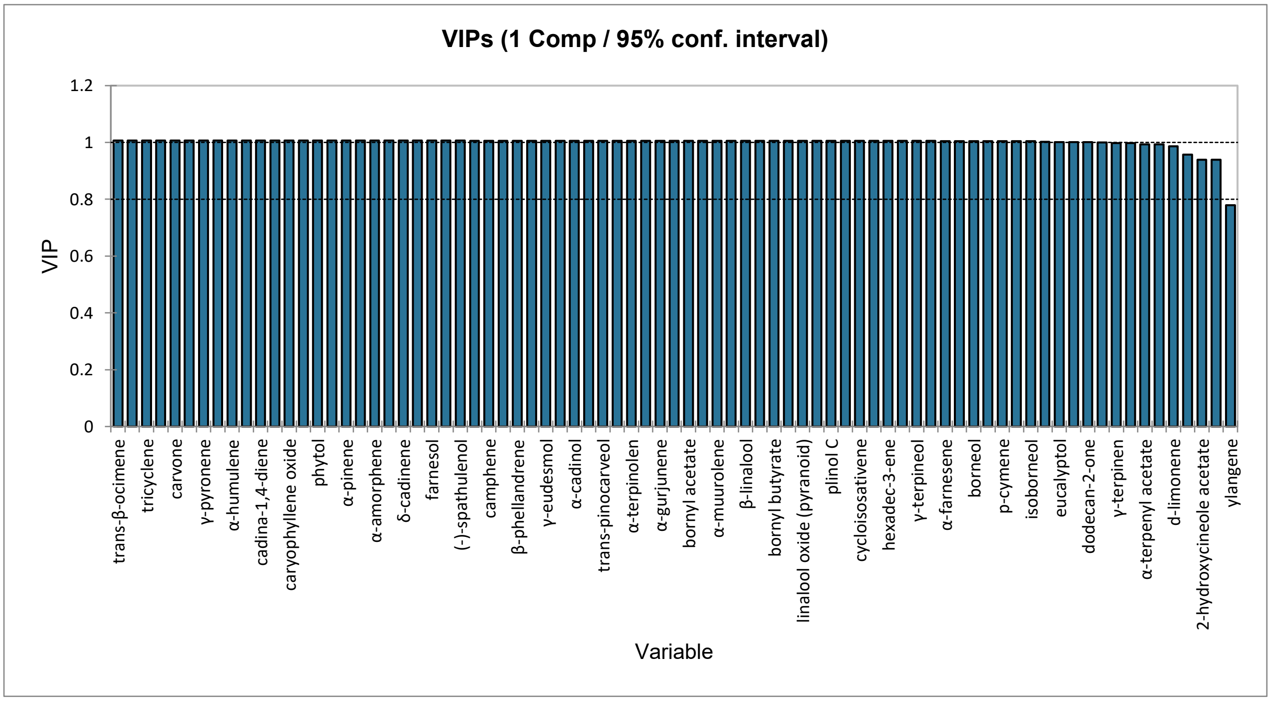

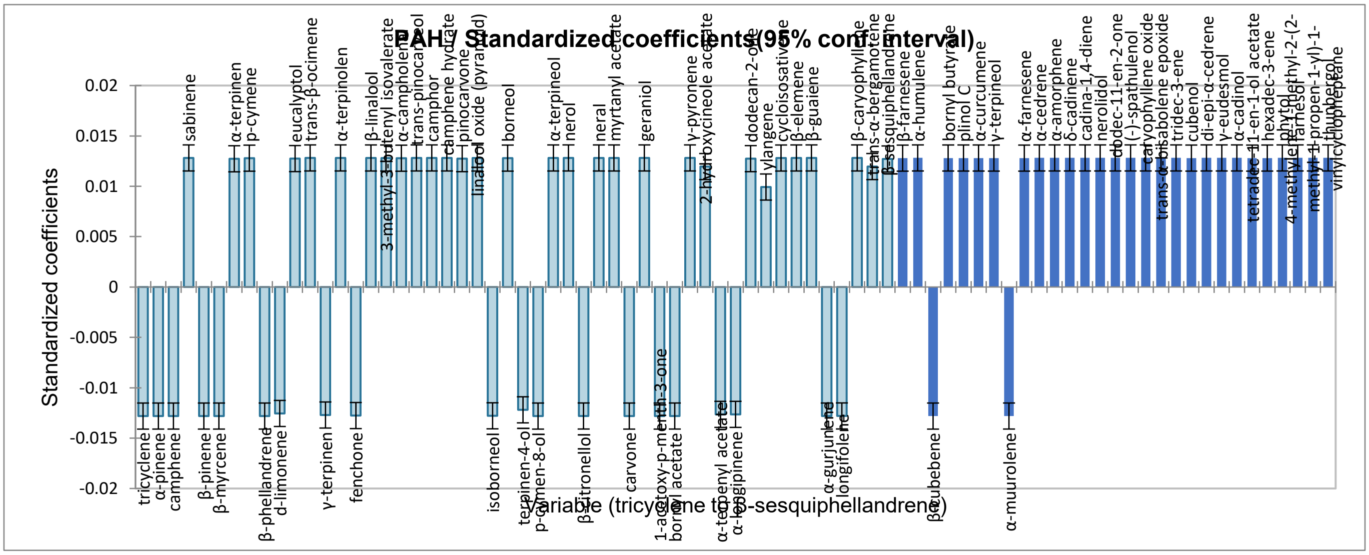
PAH 7 Standardized coefficients (95% conf. interval)
Standardized coefficients
Variable (tricyclene to β-sesquiphellandrene)
0.02
0.015
0.01
0.005
0
-0.005
-0.01
-0.015
-0.02
tricyclene
α-pinene
camphene
sabinene
β-pinene
β-myrcene
α-terpinen
p-cymene
β-phellandrene
d-limonene
eucalyptol
trans-β-ocimene
γ-terpinen
α-terpinolen
fenchone
β-linalool
isoborneol
borneol
terpinen-4-ol
p-cymen-8-ol
α-terpineol
nerol
β-citronellol
neral
myrtanyl acetate
carvone
geraniol
bornyl acetate
γ-pyronene
α-terpenyl acetate
α-longipinene
ylangene
β-elemene
β-guaiene
α-gurjunene
longifolene
β-caryophyllene
β-farnesene
α-humulene
β-cubebene
bornyl butyrate
plinol C
α-curcumene
γ-terpineol
α-muurolene
α-farnesene
α-cedrene
α-amorphene
δ-cadinene
cadina-1,4-diene
nerolidol
(-)-spathulenol
caryophyllene oxide
trans-α-bisabolene epoxide
tridec-3-ene
cubenol
di-epi-α-cedrene
γ-eudesmol
α-cadinol
hexadec-3-ene
phytol
farnesol
vinylcycloheptane

Unsupervised Principal Component Analysis (PCA) and Hierarchical Cluster Analysis (HCA) for *Salvia officinalis*

	F1	F2	F3
Eigenvalue	45.434	5.718	1.847
Variability (%)	85.725	10.790	3.485
Cumulative %	85.725	96.515	100.000

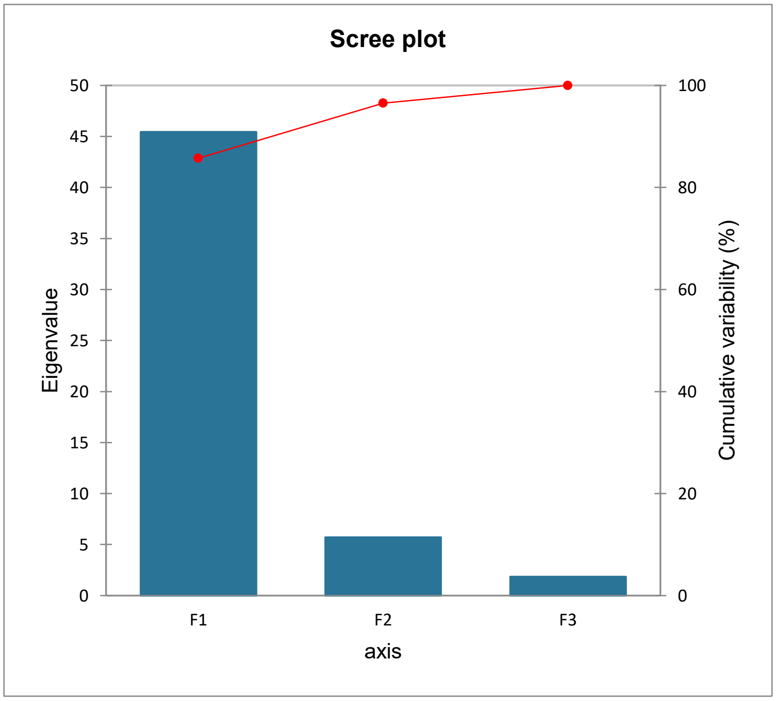

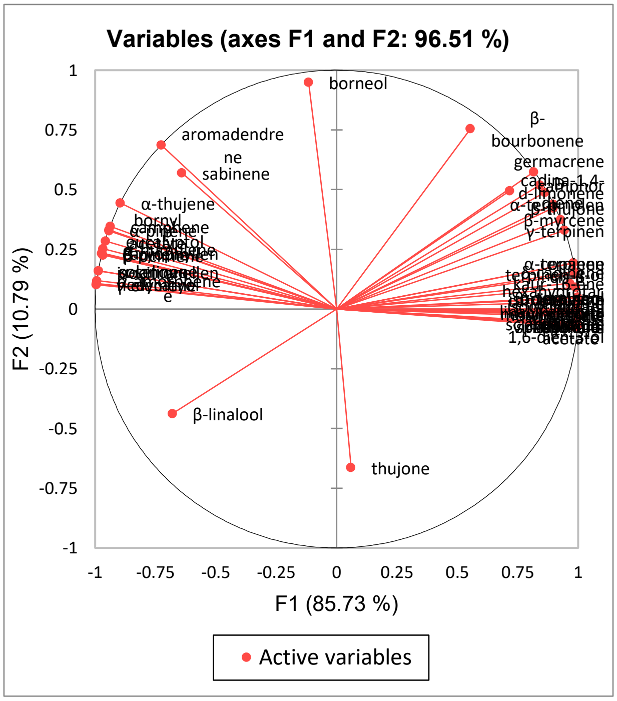

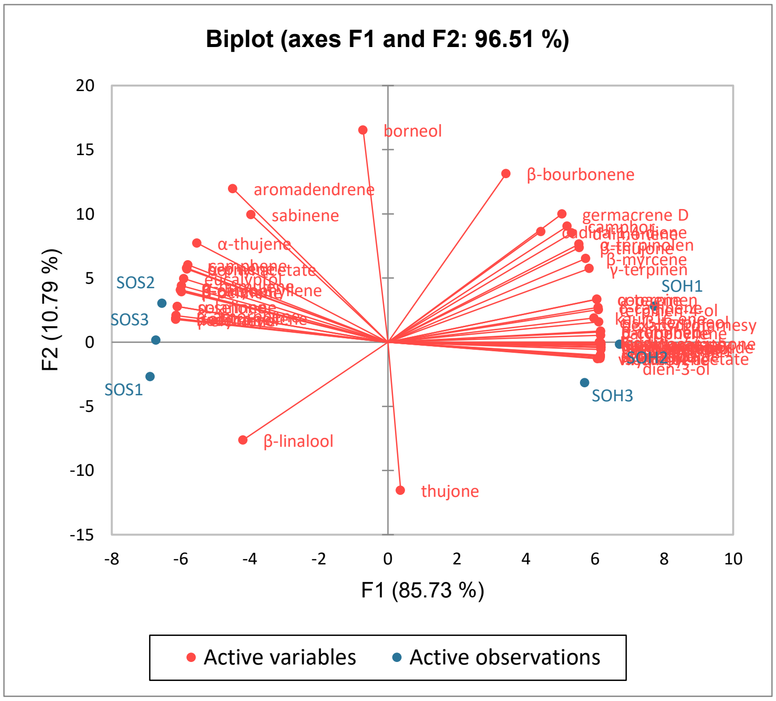

Biplot (axes F1 and F2: 96.51 %)
F2 (10.79 %)
F1 (85.73 %)
borneol
β-bourbonene
aromadendrene
sabinene
α-thujene
germacrene D
camphor
β-thujone
β-myrcene
γ-terpinen
SOS2
SOS3
SOS1
SOH1
SOH2
SOH3
dien-3-ol
β-linalool
thujone
Active variables
Active observations

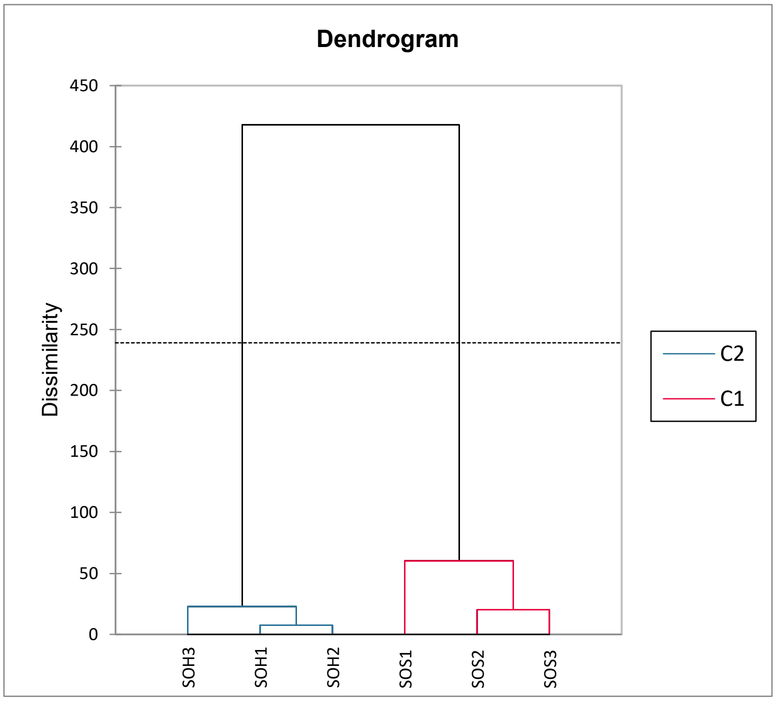
Dendrogram
Dissimilarity
450
400
350
300
250
200
150
100
50
0
SOH3
SOH1
SOH2
SOS1
SOS2
SOS3
C2
C1

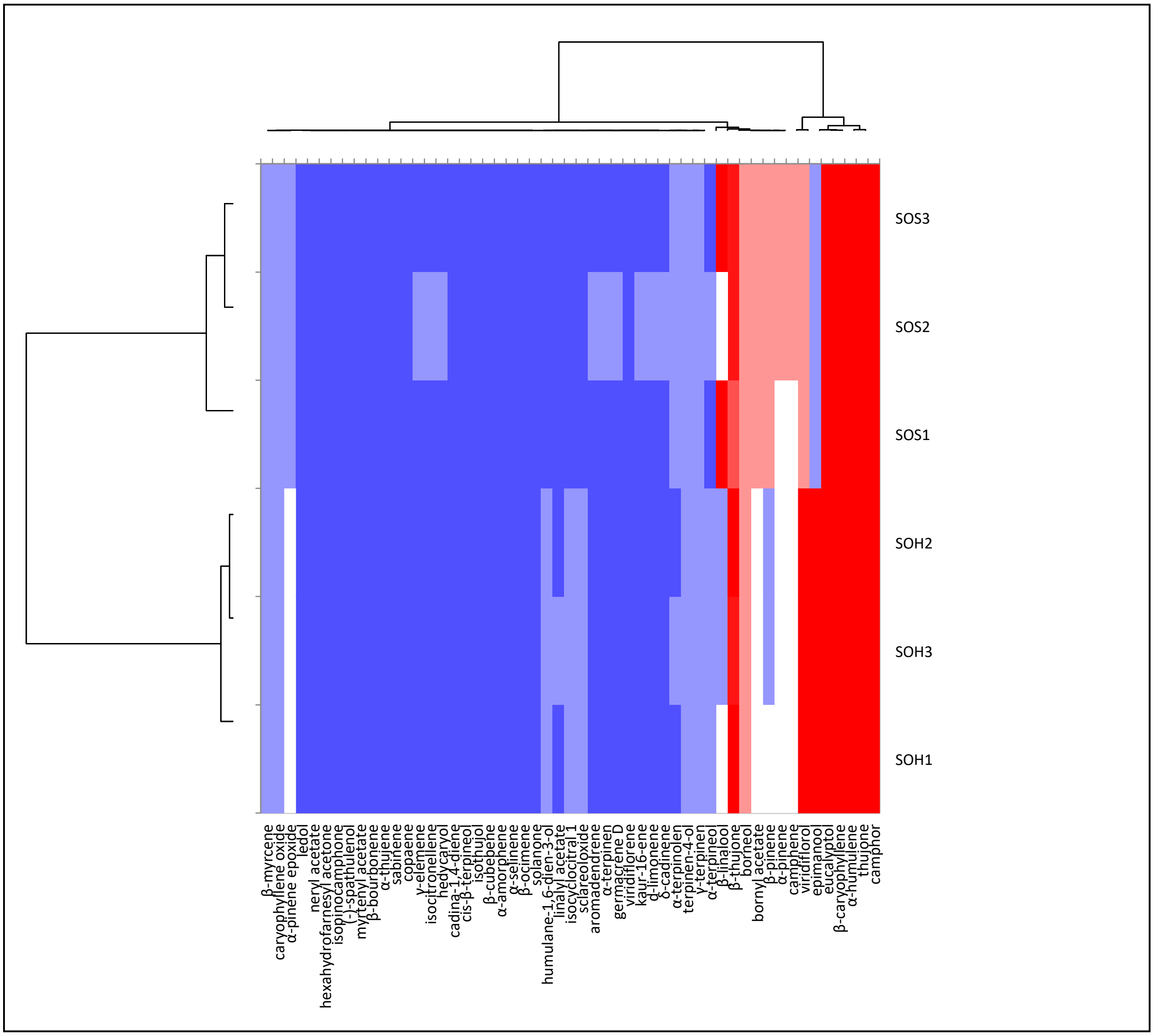
SOS3
SOS2
SOS1
SOH2
SOH3
SOH1
β-myrcene
caryophyllene oxide
α-pinene epoxide
ledol
neryl acetate
hexahydrofarnesyl acetone
isopinocamphone
(-)-spathulenol
myrtenyl acetate
β-bourbonene
α-thujene
sabinene
copaene
γ-elemene
isocitronellene
hedycaryol
cadina-1,4-diene
cis-β-terpineol
isothujol
β-cubebene
α-amorphene
α-selinene
β-ocimene
solanone
humulane-1,6-dien-3-ol
linalyl acetate
isocyclocitral 1
sclareoloxide
aromadendrene
α-terpinen
germacrene D
viridiflorene
kaur-16-ene
d-limonene
δ-cadinene
α-terpinolen
terpinen-4-ol
γ-terpinen
α-terpineol
β-linalool
β-thujone
borneol
bornyl acetate
β-pinene
α-pinene
camphene
viridiflorol
epimanool
eucalyptol
β-caryophyllene
α-humulene
thujone
camphor

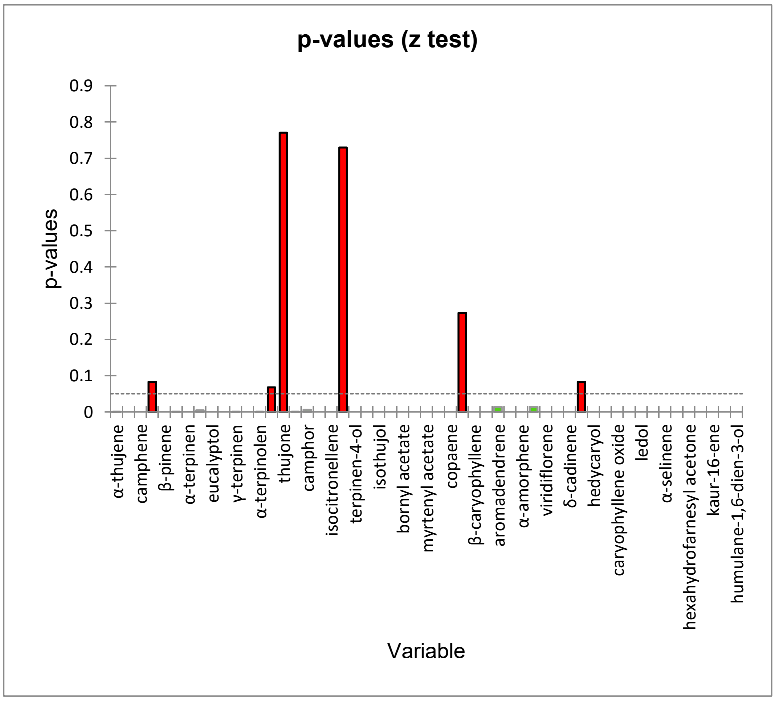

p-values (z test)
p-values
0.9
0.8
0.7
0.6
0.5
0.4
0.3
0.2
0.1
0
α-thujene
camphene
β-pinene
α-terpinen
eucalyptol
γ-terpinen
α-terpinolen
thujone
camphor
isocitronellene
terpinen-4-ol
isothujol
bornyl acetate
myrtenyl acetate
copaene
β-caryophyllene
aromadendrene
α-amorphene
viridiflorene
δ-cadinene
hedycaryol
caryophyllene oxide
ledol
α-selinene
hexahydrofarnesyl acetone
kaur-16-ene
humulane-1,6-dien-3-ol
Variable

Statistic	Comp1
Q^2 cum	0.9886309
R^2Y cum	0.9937754
R^2X cum	0.8571861

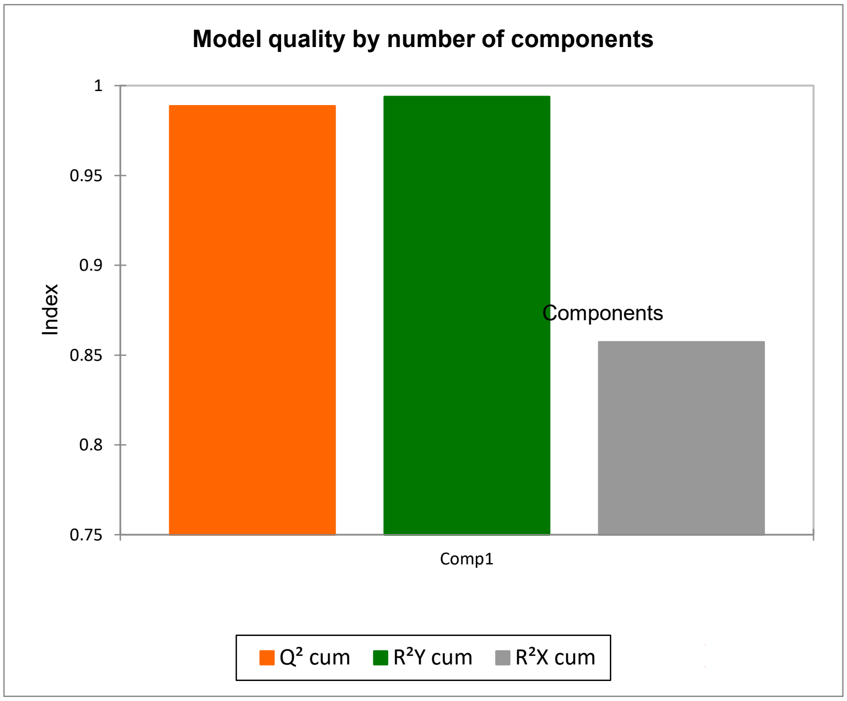

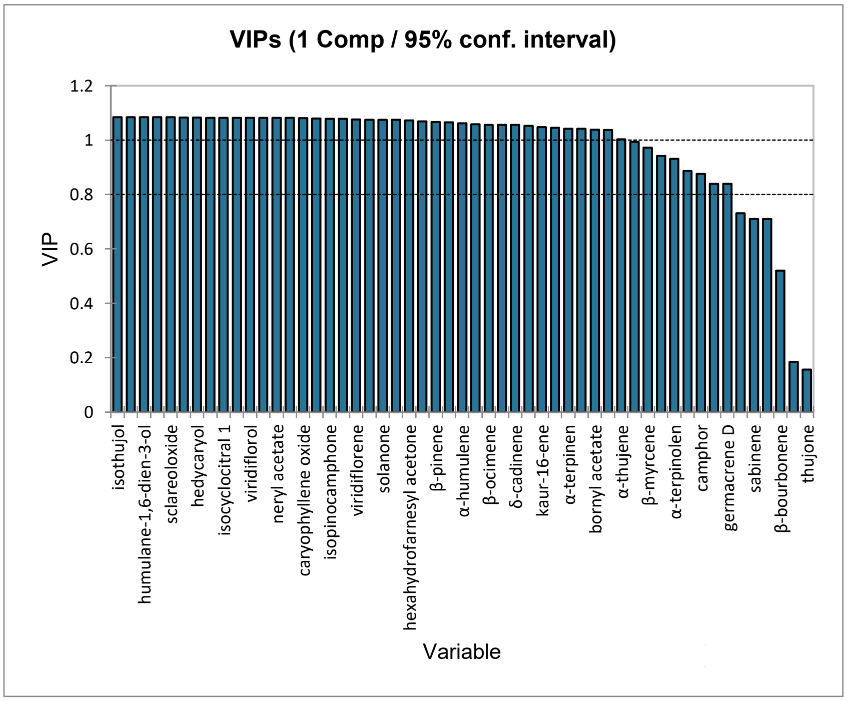

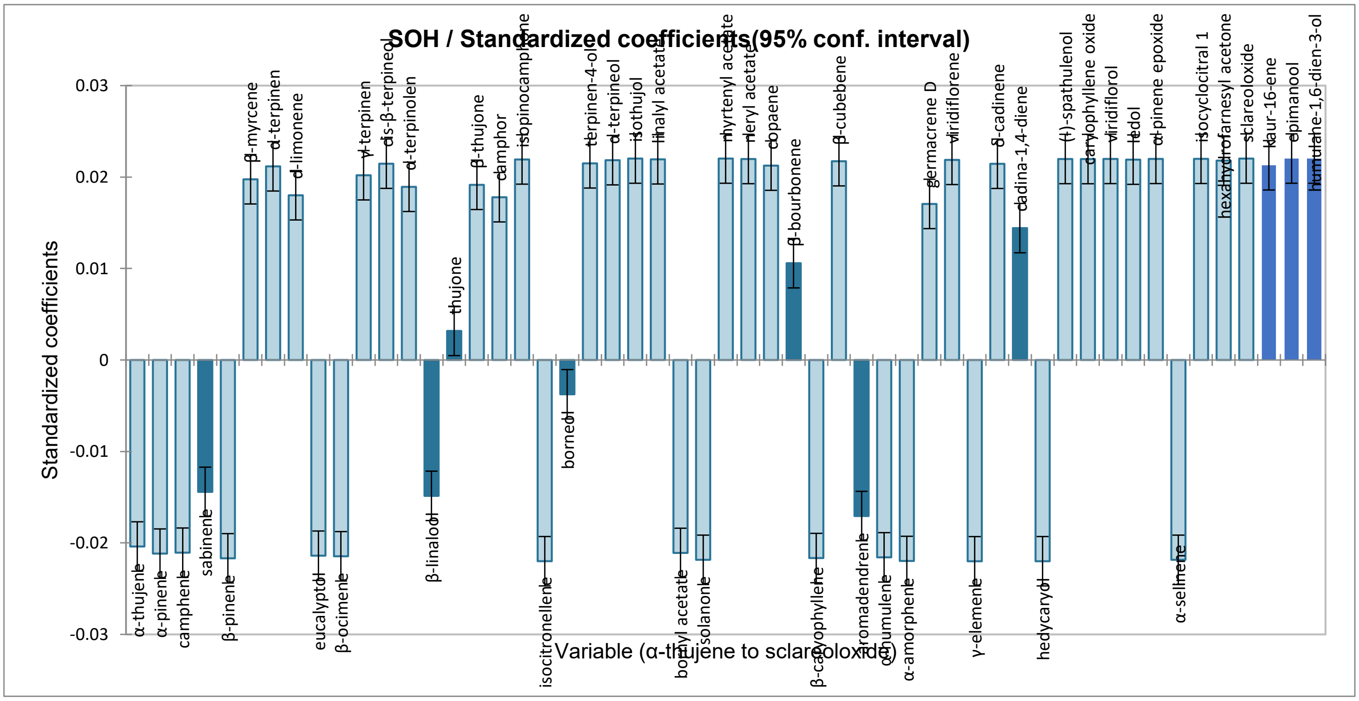

SOH / Standardized coefficients(95% conf. interval)
Standardized coefficients
0.03
0.02
0.01
0
-0.01
-0.02
-0.03
Variable (α-thujene to sclareoloxide)
α-thujene
α-pinene
camphene
sabinene
β-pinene
β-myrcene
α-terpinen
d-limonene
eucalyptol
β-ocimene
γ-terpinen
cis-β-terpineol
α-terpinolen
β-linalool
thujone
β-thujone
camphor
isopinocamphone
isocitronellene
borneol
terpinen-4-ol
α-terpineol
isothujol
linalyl acetate
bornyl acetate
solanone
myrtenyl acetate
neryl acetate
copaene
β-bourbonene
β-caryophyllene
β-cubebene
aromadendrene
α-humulene
α-amorphene
germacrene D
viridiflorene
γ-elemene
δ-cadinene
cadina-1,4-diene
hedycaryol
(+)-spathulenol
caryophyllene oxide
viridiflorol
ledol
α-pinene epoxide
α-selinene
isocyclocitral 1
hexahydrofarnesyl acetone
sclareoloxide
kaur-16-ene
epimanool
humulane-1,6-dien-3-ol
